# Inferring transmission trees to guide targeting of interventions against visceral leishmaniasis and post–kala-azar dermal leishmaniasis

**DOI:** 10.1073/pnas.2002731117

**Published:** 2020-09-24

**Authors:** Lloyd A. C. Chapman, Simon E. F. Spencer, Timothy M. Pollington, Chris P. Jewell, Dinesh Mondal, Jorge Alvar, T. Déirdre Hollingsworth, Mary M. Cameron, Caryn Bern, Graham F. Medley

**Affiliations:** ^a^Department of Global Health and Development, London School of Hygiene and Tropical Medicine, London WC1H 9SH, United Kingdom;; ^b^Centre for Mathematical Modelling of Infectious Disease, London School of Hygiene and Tropical Medicine, London WC1E 7HT, United Kingdom;; ^c^Zeeman Institute, University of Warwick, Coventry CV4 7AL, United Kingdom;; ^d^Big Data Institute, Li Ka Shing Centre for Health Information and Discovery, University of Oxford, Oxford OX3 7LF, United Kingdom;; ^e^Centre for Health Informatics, Computing and Statistics, Lancaster University, Lancaster LA1 4YW, United Kingdom;; ^f^Nutrition and Clinical Services Division, International Centre for Diarrhoeal Disease Research Bangladesh, Dhaka 1212, Bangladesh;; ^g^Research and Development, Drugs for Neglected Diseases Initiative, Geneva 1202, Switzerland;; ^h^Department of Disease Control, London School of Hygiene and Tropical Medicine, London WC1E 7HT, United Kingdom;; ^i^Department of Epidemiology and Biostatistics, University of California, San Francisco, CA 94158-2549

**Keywords:** visceral leishmaniasis, post–kala-azar dermal leishmaniasis, spatiotemporal transmission, transmission tree, Bayesian inference

## Abstract

Understanding of spatiotemporal transmission of infectious diseases has improved significantly in recent years. Advances in Bayesian inference methods for individual-level geo-located epidemiological data have enabled reconstruction of transmission trees and quantification of disease spread in space and time, while accounting for uncertainty in missing data. However, these methods have rarely been applied to endemic diseases or ones in which asymptomatic infection plays a role, for which additional estimation methods are required. Here, we develop such methods to analyze longitudinal incidence data on visceral leishmaniasis (VL) and its sequela, post–kala-azar dermal leishmaniasis (PKDL), in a highly endemic community in Bangladesh. Incorporating recent data on VL and PKDL infectiousness, we show that while VL cases drive transmission when incidence is high, the contribution of PKDL increases significantly as VL incidence declines (reaching 55% in this setting). Transmission is highly focal: 85% of mean distances from inferred infectors to their secondary VL cases were <300 m, and estimated average times from infector onset to secondary case infection were <4 mo for 88% of VL infectors, but up to 2.9 y for PKDL infectors. Estimated numbers of secondary cases per VL and PKDL case varied from 0 to 6 and were strongly correlated with the infector’s duration of symptoms. Counterfactual simulations suggest that prevention of PKDL could have reduced overall VL incidence by up to 25%. These results highlight the need for prompt detection and treatment of PKDL to achieve VL elimination in the Indian subcontinent and provide quantitative estimates to guide spatiotemporally targeted interventions against VL.

## Significance

Methods for analyzing individual-level geo-located disease data have existed for some time, but have rarely been used to analyze endemic human diseases. Here we apply such methods to nearly a decade’s worth of uniquely detailed epidemiological data on incidence of the deadly vector-borne disease visceral leishmaniasis (VL) and its secondary condition, post–kala-azar dermal leishmaniasis (PKDL), to quantify the spread of infection around cases in space and time by inferring who infected whom, and estimate the relative contribution of different infection states to transmission. Our findings highlight the key role long diagnosis delays and PKDL play in maintaining VL transmission. This detailed characterization of the spatiotemporal transmission of VL will help inform targeting of interventions around VL and PKDL cases.

Spatiotemporal heterogeneity in incidence is a hallmark of infectious diseases. Insight into this heterogeneity has increased considerably in recent years due to greater availability of geo-located individual-level epidemiological data and the development of sophisticated statistical inference methods for partially observed transmission processes (1–6). These methods have been developed for epidemics, in which the immune status of the population is known, and for diseases with a short time course that are relatively easily diagnosed, such as measles, influenza, and foot-and-mouth disease (3, 4, 7). Here, we extend these methods to a slowly progressing endemic disease of humans in which asymptomatic infection plays an important role.

We analyze detailed longitudinal individual-level data on incidence of visceral leishmaniasis (VL) and its sequela, post–kala-azar dermal leishmaniasis (PKDL), in a highly endemic community in Fulbaria, Bangladesh (8). VL, also known as kala-azar, is a lethal sandfly-borne parasitic disease targeted for elimination as a public health problem (<1 case per 10,000 people per year at subdistrict/district level depending on the country) in the Indian subcontinent (ISC) by 2020 (9). It has a disproportionate impact among the most vulnerable groups in the population in the ISC (10). PKDL is a nonlethal skin condition that occurs after treatment for VL in 5 to 20% of cases in the ISC and less frequently in individuals who report no history of prior VL (8, 11). It is characterized by skin lesions of differing severity and parasite load, ranging from macules and papules (least severe, lowest load) to nodules (most severe, highest load) (12). We estimate the relative contributions of different disease states (VL, PKDL, and asymptomatic infection) to transmission and quantify the rate of spread of infection around infected individuals in space and time by reconstructing transmission trees. Our analysis provides insight into the spatiotemporal spread of visceral leishmaniasis as well as quantitative estimates that can guide the targeting of interventions, such as active case detection and indoor residual spraying (IRS) of insecticide, around VL and PKDL cases.

PKDL cases are believed to play a role in transmission of VL as historical and recent xenodiagnosis studies have shown that all PKDL forms are infectious toward sandflies (12–14), and a 1992 study in West Bengal, India, suggested that PKDL cases are capable of initiating a VL outbreak in a susceptible community (15). Furthermore, PKDL cases typically have long durations of symptoms before treatment and often go undiagnosed as the disease is not systemic (16, 17). While VL incidence has declined considerably throughout the ISC since 2011 (by >85%, from ∼37,000 cases in 2011 to ∼4,700 in 2018) (18, 19), reported numbers of PKDL diagnoses increased from 590 in 2012 to 2,090 in 2017 before falling to 1,363 in 2018 (19, 20). PKDL has therefore been recognized as a major potential threat to the VL elimination program in the ISC (11), which has led to increased active PKDL case detection. Nevertheless, the contribution of PKDL to transmission in field settings still urgently needs to be quantified.

Although the incidence of asymptomatic infection is 4 to 17 times higher than that of symptomatic infection in the ISC (21), the extent to which asymptomatic individuals contribute to transmission is still unknown (22, 23). What is clear is that asymptomatic infection plays a role in transmission through generating herd immunity, since a significant proportion of asymptomatically infected individuals develop protective cell-mediated immunity against VL following infection, as measured by positivity on the leishmanin skin test (LST) (24–27). Several studies have shown that asymptomatic infection is spatiotemporally clustered (25, 28), and therefore immunity is also likely to be spatially clustered, but so far no transmission models have accounted for this (23). Since most surveillance data and data from epidemiological studies do not contain information about numbers of asymptomatically infected individuals over space and time (e.g., from longitudinal serological testing), accounting for the role of asymptomatic infection in transmission at the individual level represents a substantial missing data problem. The endemic nature of the disease and high asymptomatic infection potential mean that it is necessary to infer initial infection statuses for individuals without symptomatic disease, unlike for many epidemic diseases where individuals can be assumed to be susceptible or are known to have been vaccinated. Coupled with the long and variable incubation period of VL [lasting anywhere between weeks and years but typically 2 to 6 mo (29)] and lack of data on the flight range of the *Phlebotomus argentipes* sandfly vector, these factors make inference of spatiotemporal transmission of VL particularly challenging.

By combining data from a recent xenodiagnosis study in Bangladesh (12) with geo-located data on incidence and duration of symptoms of VL and different forms of PKDL from the community study in Bangladesh and fitting them to an individual-level spatiotemporal VL transmission model, this study provides detailed insight into the changing roles of VL, PKDL, asymptomatic infection, and immunity in transmission over the course of an epidemic and estimates of numbers of secondary cases and infections generated by individual VL and PKDL cases. The Bayesian data augmentation framework that we develop to fit the model accounts for the unobserved infection times of VL cases, the missing data on asymptomatic infections, individuals’ unobserved initial infection statuses, migration of individuals, and uncertainty in infection sources and could be readily adapted to analyze spatiotemporal transmission of other endemic diseases in which asymptomatic infection plays a hidden role.

## Study Data.

**Fig. 1.**
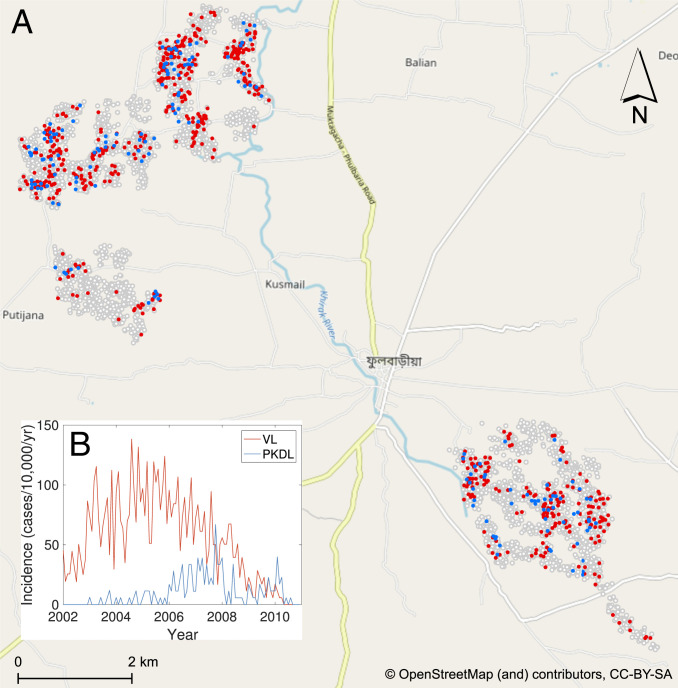
(*A*) Map of the study area showing the households that had VL cases (red), PKDL cases (blue), and no cases (white with gray outline) with onset between 2002 and 2010. Household locations are jittered slightly to preserve patient anonymity. (*B*) Observed incidence of VL and PKDL for the whole study area by month of onset, 2002 to 2010.

We analyze detailed demographic and disease data on 24,781 individuals living in 5,118 households in 19 paras (hamlets) situated in two large clusters in a 12 × 12-km area in Fulbaria Upazila, Mymensingh district, Bangladesh from 2002 to 2010 (Fig. 1*A*). The data from this study are fully described elsewhere (8, 30). Briefly, month of onset of symptoms, treatment, relapse, and relapse treatment were recorded for VL cases and PKDL cases with onset between 2002 and 2010 (retrospectively for cases with onset before 2007), and year of onset was recorded for VL cases with onset before 2002. There were 1,018 VL cases and 190 PKDL cases with onset between January 2002 and December 2010 in the study area and 413 VL cases with onset before January 2002.

Over the whole study area, VL incidence followed an epidemic wave, increasing from approximately 40 cases per 10,000/y in 2002 to ∼90 cases per 10,000/y in 2005 before declining to <5 cases per 10,000/y in 2010 (Fig. 1*B*). PKDL incidence followed a similar pattern but lagging VL incidence by roughly 2 y, peaking at 30 cases per 10,000/y in 2007. However, VL and PKDL incidence varied considerably across paras (average para-level incidences: VL, 18 to 124 cases per 10,000/y; PKDL, 0 to 31 cases per 10,000/y; *SI Appendix*, Table S6) and time (range of annual para-level incidences: VL, 0 to 414 cases per 10,000/y; PKDL, 0 to 120 cases per 10,000/y; *SI Appendix*, Fig. S18).

## Results

### Model Comparison.

Different versions of the spatiotemporal transmission model described in *Materials and Methods*, in which decrease in infection risk with distance from an infectious individual is characterized by an exponentially decaying spatial kernel function, were fitted to the data. These comprised models with and without extra within-household transmission (over and above that from being at zero distance from an infectious individual) and with different presymptomatic and asymptomatic relative infectiousness. Models with additional within-household transmission fitted the data significantly better than those without according to a version of the deviance information criterion (DIC) appropriate for missing-data models (*SI Appendix*) (31). The range of presymptomatic and asymptomatic relative infectiousness tested (0 to 2% of that of VL cases) was chosen based on the 95% confidence interval of the probability that asymptomatic individuals can transmit to sandflies (0,0.023) from a xenodiagnosis study in India in which 0 of 183 asymptomatic individuals infected sandflies (32). Apart from the spatial transmission rate constant, which decreased with increasing presymptomatic/asymptomatic infectiousness, estimates of the transmission parameters were highly consistent across this presymptomatic/asymptomatic infectiousness range (*SI Appendix*, Table S4). Here we present the results from the model with 2% presymptomatic/asymptomatic infectiousness, since there is evidence from outbreak investigations suggesting that asymptomatic individuals can, at least on occasion, infect sandflies (27, 33) and because it represents the most conservative assumption in terms of our aim of estimating the contribution of PKDL to transmission.

### Parameter Estimates.

We estimated the transmission model parameters and unobserved data using the Markov chain Monte Carlo (MCMC) algorithm described in *Materials and Methods* and *SI Appendix*. The posterior distributions obtained for the model parameters are shown in *SI Appendix*, Fig. S6 and the corresponding posterior modes and 95% credible intervals (CI) are given in Table 1.

**Table 1.** Transmission parameter estimates from the spatiotemporal model

Parameter	Mode	95% CI*
Risk of developing VL^†^ if living for 1 mo in the same household as a		
VL case	0.018	(0.014,0.026)
PKDL case		
Macular/papular	0.009	(0.007,0.014)
Plaque^‡^	0.017	(0.012,0.023)
Nodular	0.023	(0.017,0.033)
Asymptomatic individual	0.00037	(0.00027,0.00053)
Risk of asymptomatic infection^†^ if living for 1 mo in the same household as a		
VL case	0.099	(0.074,0.140)
PKDL case		
Macular/papular	0.053	(0.039,0.074)
Plaque^‡^	0.092	(0.067,0.127)
Nodular	0.125	(0.095,0.175)
Asymptomatic individual	0.0021	(0.0015,0.0030)
Risk of developing VL from background transmission each month	$6.6\times 10^{-5}$	($3.4\times 10^{-5}$, $10\times 10^{-5}$)
Risk of asymptomatic infection from background transmission each month	$3.7\times 10^{-4}$	($1.9\times 10^{-4}$, $5.9\times 10^{-4}$)
Decrease in risk of infection with distance from an infectious individual (per 100 m)^§^	57.7%	(52.0%,63.1%)
Hazard ratio for increase in infection risk from living in the same household	11.6	(7.3,16.6)
as an infectious individual compared with living just outside		

* CI, credible interval, calculated as the 95% highest posterior density interval.

^†^ Risk of subsequent VL/asymptomatic infection if susceptible.

^‡^ Based on assumed infectiousness.

^§^ In the absence of background transmission and relative to living directly outside the case household.

Based on the relative infectiousness of VL and the different types of PKDL from the xenodiagnostic data (*SI Appendix*, Table S1), in the absence of any other sources of transmission, the estimated probability of being infected and developing VL if living in the same household as a single symptomatic individual for 1 mo following his/her onset was 0.018 (95% CI: 0.014, 0.026) for VL and ranged from 0.009 to 0.023 (95% CIs: (0.007,0.014) to (0.017, 0.033)) for macular/papular PKDL to nodular PKDL. Living in the same household as a single asymptomatic individual, the monthly risk of VL was only 0.00037 (95% CI: 0.00027, 0.00053), if asymptomatic individuals are 2% as infectious as VL cases.

The risk of infection if living in the same household as an infectious individual was estimated to be more than 10 times higher than that if living directly outside the household of an infectious individual (hazard ratio = 11.6), with a 95% CI well above 1 (7.3, 16.6). The estimated spatial kernel (*SI Appendix*, Fig. S19) around each infectious individual shows a relatively rapid decay in infection risk with distance outside the individual’s household, the risk halving over a distance of 87 m (95% CI: 73, 101 m).

The inferred prevalences of the different infection states in the model illustrate the increasing level of immunity in the population over the course of the epidemic generated by asymptomatic infection (*SI Appendix*, Fig. S20).

### Contribution of PKDL and Asymptomatic Infection to Transmission.

**Fig. 2.**
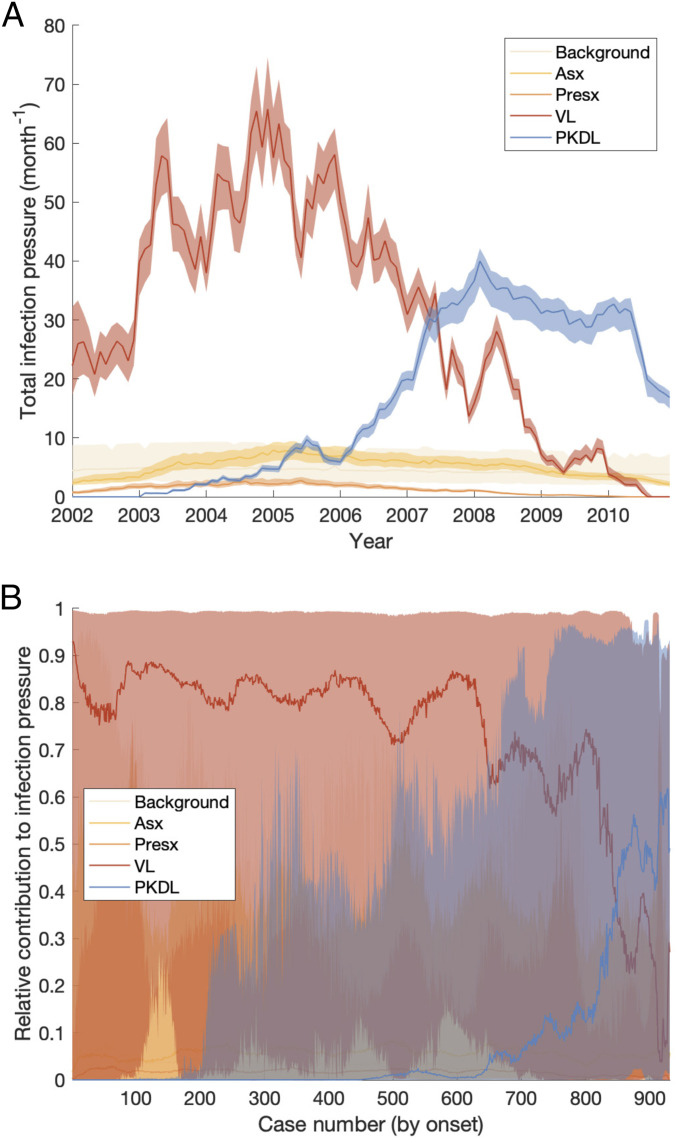
(*A* and *B*) Contributions of background transmission, asymptomatic (Asx) individuals, presymptomatic (Presx) individuals, VL cases, and PKDL cases to (*A*) the total infection pressure on susceptible individuals and (*B*) the individual infection pressures on VL cases at their infection times (in relative terms). Note that time is nonlinear in *B* since cases are ordered by their onset time. Solid lines show modes in *A* and medians in *B*; shaded regions show 95% CIs. The relative contribution of PKDL to the infection pressure on the seven VL cases with onset in 2010 in *B* is lower than to the infection pressure on susceptible individuals in 2010 in *A* since the 2010 VL cases all had onset before May and were therefore most likely infected in 2009 when the relative contribution of VL was higher.

We assess the contribution of different infectious groups to transmission in terms of their relative contribution to the transmission experienced by susceptible individuals (Fig. 2*A* and *SI Appendix*, Fig. S21). The contribution of VL cases was fairly stable at around 75% from 2002 to the end of 2004 before decreasing steadily to 0 at the end of the epidemic, while the contribution of PKDL cases increased from 0 in 2002 to ∼75% in 2010 (95% CI: 63, 81%). Only a small proportion of the total infection pressure on susceptible individuals, varying between 8 and 15% over the course of the epidemic, was estimated to have come from asymptomatic and presymptomatic individuals.

Fig. 2*B* shows the breakdown of the individual infection pressures on VL cases at their infection times and indicates that the contribution of PKDL to these infection pressures grew from 0% at the start of the epidemic to approximately 55% (95% CI: 2, 92%) for the cases with onset in 2010. Unsurprisingly, given the uncertainty in the infection times of the VL cases, the credible intervals for the relative contributions of each infection source to the infection pressures on the cases at their infection times are very broad.

### Reconstructing the Transmission Tree.

**Fig. 3.**
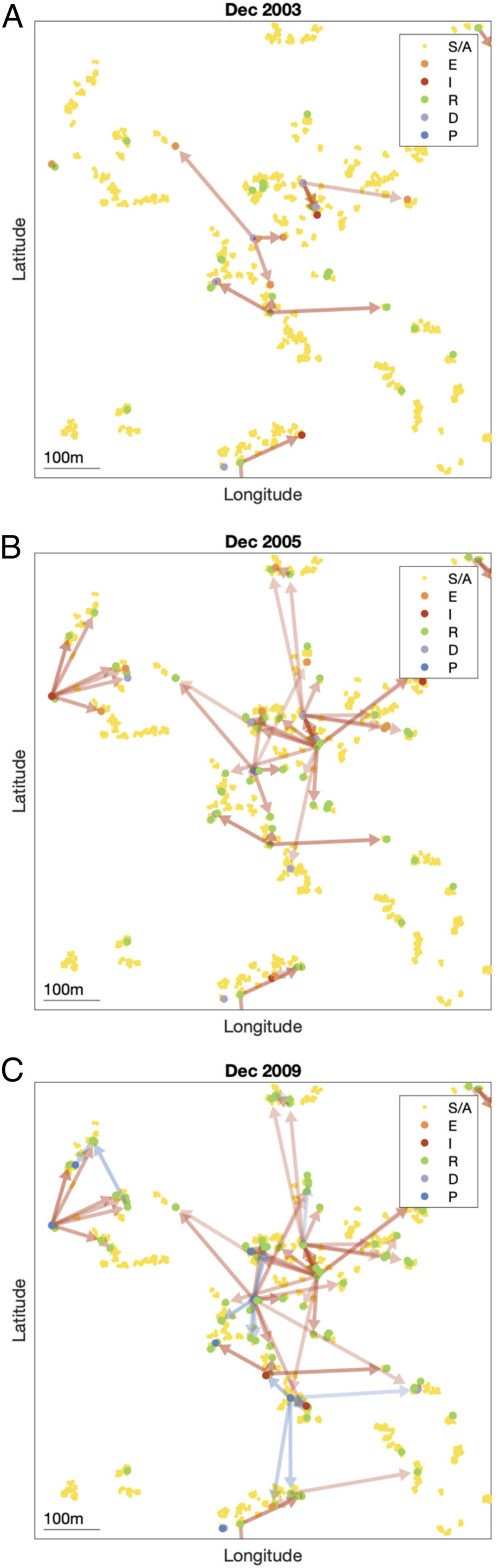
(*A*–*C*) Inferred transmission tree in part of the southeast cluster of villages at different stages of the epidemic: (*A*) December 2003, (*B*) December 2005, and (*C*) December 2009. Dots show individuals colored by their infection state (see key). Arrows show the most likely source of infection for each case infected up to that point in time over 1,000 sampled transmission trees and are colored by the type of infection source and shaded according to the proportion of trees in which that individual was the most likely infector (darker shading indicating a higher proportion). Asymptomatic infections are not shown for clarity. S/A, susceptible or asymptomatic; E, presymptomatic; I, VL; R, recovered; D, dormantly infected; P, PKDL (*SI Appendix*). GPS locations of individuals are jittered slightly so that individuals from the same household are more visible. An animated version showing all months is provided in Movie S1.

By sampling 1,000 transmission trees from the joint posterior distribution of the transmission parameters and the unobserved data (as described in *Materials and Methods*), we can build a picture of the most likely source of infection for each case and how infection spreads in space and time. Fig. 3 shows the transmission tree at different points in time in part of the southeast cluster of villages. Early in the epidemic and at its peak (Fig. 3 *A* and *B*), most new infections were due to VL cases. Toward the end of the epidemic, some infections were most likely due to PKDL cases and there was some saturation of infection around VL cases (Fig. 3*C*). The inferred patterns of transmission suggest that disease did not spread radially outward from index cases over time, but instead made a combination of short and long jumps around cases with long durations of symptoms and households with multiple cases.

### Transmission Distances and Times.

Having reconstructed a set of samples of the transmission tree as described above, we can use them to calculate the mean distance from each VL/PKDL infector to the VL-case infectees and the mean times between the onset and the infections of the VL infectees, to assess how far and how quickly interventions need to be performed around VL and PKDL cases.

**Fig. 4.**
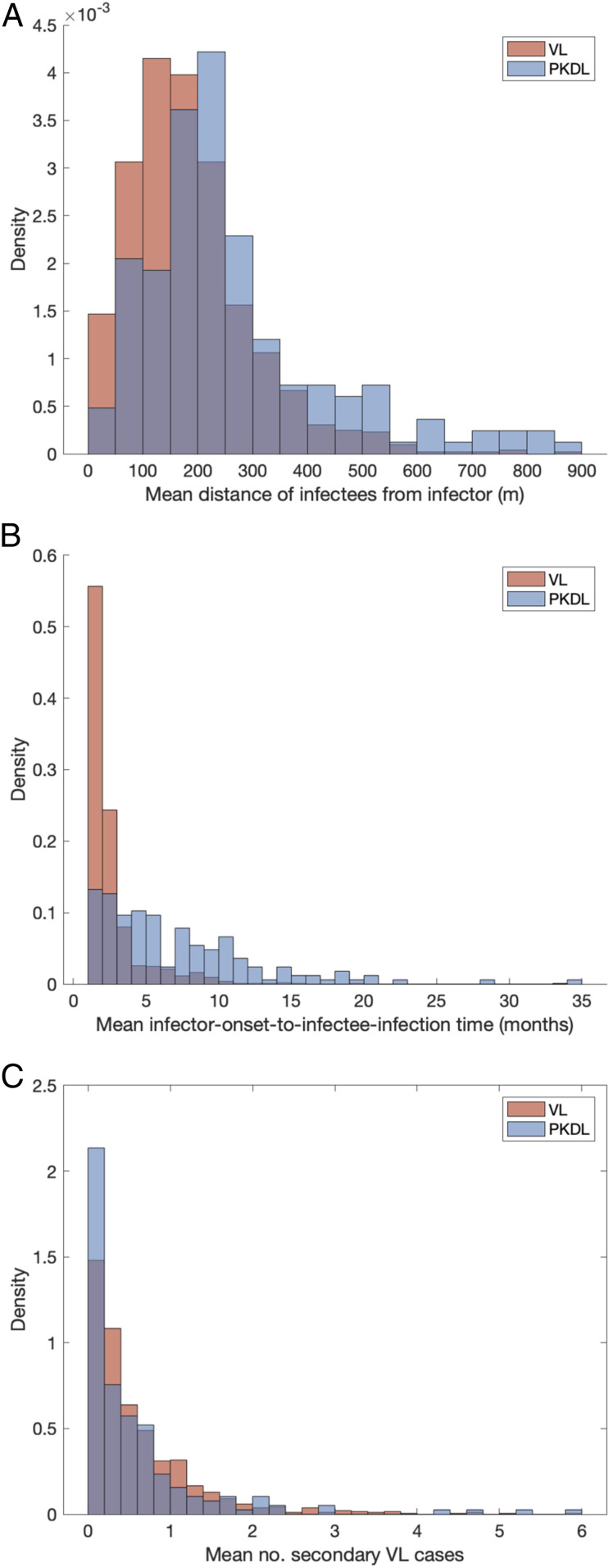
(*A*) Mean distances from VL and PKDL infectors to their VL infectees. (*B*) Mean times from symptom onset of VL and PKDL infectors to the infections of their VL infectees. (*C*) Distributions of mean numbers of secondary VL cases per VL case and PKDL case.

Fig. 4*A* shows that the mean distances to VL infectees for VL and PKDL cases are mostly within 500 m but tend to be greater for PKDL cases (median 221 m, interquartile range [IQR]: 163, 314 m) than VL cases (median 167 m, IQR: 106, 236 m), reflecting the fact that around PKDL cases there has typically already been considerable transmission from prior VL and therefore development of immunity in asymptomatically infected individuals. However, the mean times between infector onset and VL-infectee infections are much greater for PKDL cases (median 5.6 mo, IQR: 3.0, 9.7 mo) than for VL cases (median 1.9 mo, IQR: 1.4, 2.7 mo) (Fig. 4*B*). Thus, while a similar intervention radius around new VL/PKDL cases of ∼500 m may be sufficient to capture most secondary VL cases, the time window within which interventions need to be performed to prevent secondary cases is much narrower for VL cases than for PKDL cases.

### Numbers of Secondary Infections.

Since we infer the unobserved infection times of VL cases and asymptomatic individuals as part of the MCMC algorithm, we can calculate the probability that each individual was infected by another individual conditional on the individual’s estimated infection month. Using these probabilities, we can then estimate the numbers of secondary infections generated by each infectious individual.

**Fig. 5.**
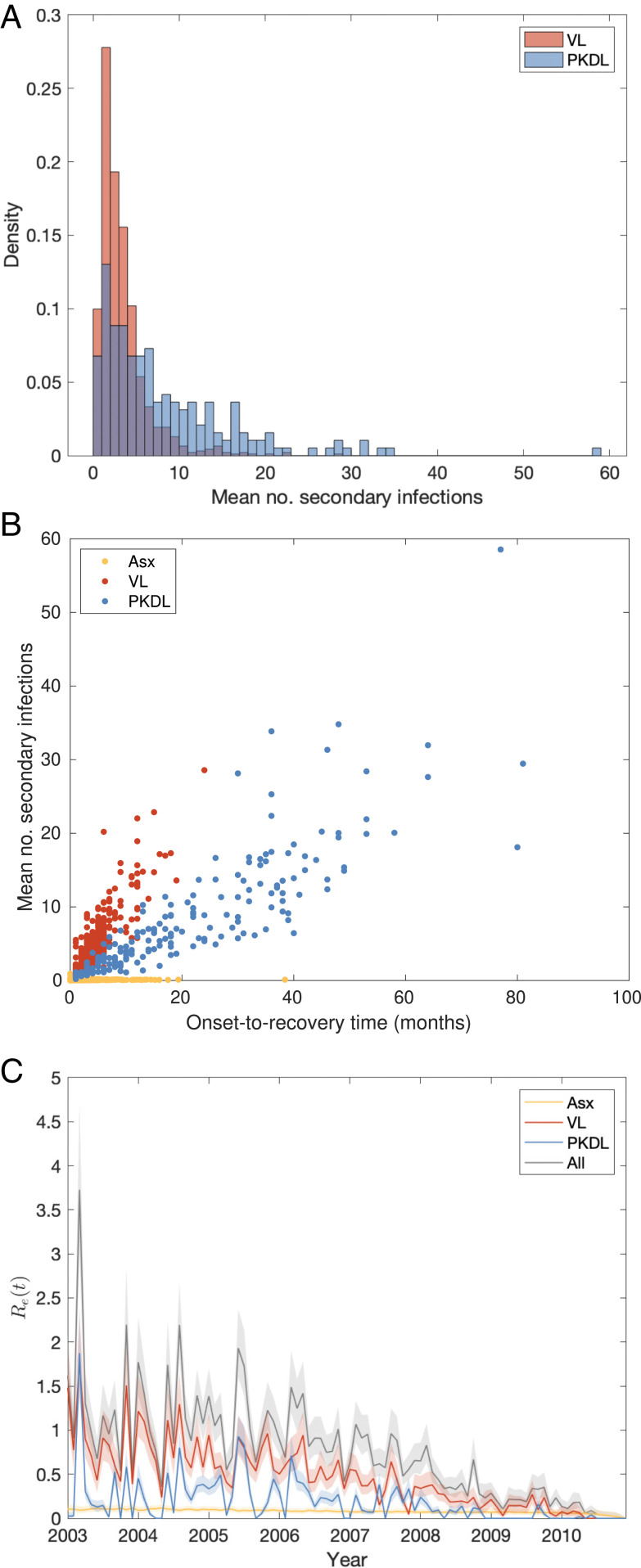
(*A*) Distributions of mean numbers of secondary infections per VL and PKDL case. (*B*) Relationship between mean number of secondary infections and onset-to-recovery time for VL and PKDL cases and infection-to-recovery time for asymptomatic individuals. (*C*) Effective reproduction number $R_{e}\left(t\right)$ with contributions from asymptomatic individuals, VL and PKDL cases. Solid lines show medians and shaded bands 95% CIs.

The mean numbers of secondary infections per VL case and per PKDL case (Fig. 5*A*) show large variation, ranging from 0.4 to 28.6 for VL and 0.2 to 58.5 for PKDL (see *SI Appendix*, Fig. S22 for the posterior distributions of the number of secondary infections generated by each VL and PKDL case), and are overdispersed, with shape parameters for fitted gamma distributions of 2.00 (95% confidence interval: 1.84, 2.17) and 1.21 (95% confidence interval: 1.01, 1.45), respectively. This indicates that some cases generate far more secondary infections than others, a phenomenon known as “superspreading,” which has been observed for a variety of diseases (34, 35) and hypothesized for VL (22, 36). The estimated mean numbers of secondary infections for asymptomatic individuals are much lower, ranging from 0 to 0.94. While the numbers of secondary infections for VL and PKDL may seem high, we note that they are the number of new presymptomatic and asymptomatic infections generated by each case and that only approximately one in seven new infections were estimated to have led to VL (29), so the estimated numbers of secondary VL cases per case are much lower (Fig. 4*C*).

As expected, the mean numbers of secondary infections generated by infectious individuals are strongly positively correlated with their durations of infectiousness (Fig. 5*B*). In particular, many PKDL cases had very long durations of symptoms (>1 y) and generated large numbers of secondary infections (>5).

The median effective reproduction number $R_{e}\left(t\right)$—the average number of secondary infections generated by individuals who became infectious in a given month t, which must remain above 1 for the disease to persist—appears to have decreased over the course of the epidemic (Fig. 5*C*), from being mostly above 1 (range: 0.4, 3.7) in 2003 to 2006 to below 1 in 2007 to 2010. We note though that in later years our estimate of $R_{e}\left(t\right)$ is subject to some downward bias due to right censoring of onsets of some VL cases infected toward the end of the study (*Discussion*).

### Impact of Preventing/Limiting PKDL.

To investigate the potential impact of stopping PKDL from occurring or reducing the duration of infectiousness of PKDL cases on incidence of VL, we created a simulation version of the transmission model and used the parameter estimates and inferred initial statuses of individuals obtained from the MCMC algorithm to run counterfactual simulations of the epidemic in the study area (see *Materials and Methods* and *SI Appendix* for further details). Based on these simulations, if there had been no PKDL, the total number of VL cases from 2002 to 2010 would have been 25% lower (95% CI: 5, 43%) (see *SI Appendix*, Fig. S23 and Table S7 for the para-level impact). This is the hypothetical maximum proportion of VL cases that could have been averted over the whole study period by preventing any PKDL, e.g., if a vaccine had been available that prevented progression to PKDL (37). However, even if the mean duration of infectiousness of PKDL had been only halved (from 18 to 9 mo)—which represents a more realistically achievable target in the near future through improved active case detection—the simulations suggest the total number of VL cases would have been 9% lower (95% CI: −15, 29%). If we consider only the last 4 y of the study—the period in which PKDL cases became the dominant source of transmission—the results suggest complete prevention of PKDL and halving the duration of infectiousness of PKDL cases would have reduced VL incidence by 46% (95% CI: 18, 70%) and 17% (95% CI: −21, 47%) respectively.

## Discussion

In this study we have estimated the contribution of PKDL to transmission of VL accounting for spatiotemporal clustering of VL and PKDL and unobserved asymptomatic infection. We have combined infectiousness data from xenodiagnostic studies with geo-located VL and PKDL incidence data to reconstruct transmission trees of the spread of VL through a community and estimate individual-level numbers of secondary infections.

Our results support the conclusion that PKDL poses a significant threat to the VL elimination program in the Indian subcontinent. While VL cases drive transmission when VL incidence is high during the peak years of an epidemic, the contribution of PKDL to transmission increases as VL prevalence decreases and PKDL prevalence increases in the downward phase of an epidemic (*SI Appendix*, Fig. S20*B*). This mirrors the current situation in Bangladesh and India, where VL incidence has been decreasing since 2011 (18, 19), but reported numbers of PKDL cases suggest PKDL prevalence is higher than VL prevalence in some areas (19).

In the study area in Bangladesh the contribution of PKDL (in terms of contribution to new symptomatic infections) grew from close to 0% in the upward phase of the epidemic in 2002 to 2005 to approximately 55% at the end of the epidemic in 2010. In light of the current low VL incidence and considerable numbers of PKDL cases being reported in much of the Indian subcontinent, this suggests that measures need to be taken to ensure all PKDL cases are detected and treated to maintain reduced transmission. This will require improvements in both active PKDL case detection, e.g., through comprehensive long-term follow-up of VL cases, and diagnostic tests and algorithms and treatment regimens for PKDL (11, 17).

There is considerable heterogeneity in the estimated contribution of individual VL cases and PKDL cases to transmission in terms of the numbers of secondary infections they generate, which is chiefly driven by variation in their onset-to-recovery times (Fig. 5*B*). As expected, individuals with long onset-to-recovery times contribute most to new infections, acting as superspreaders who generate many times more infections than the average case. These individuals play an important role in maintaining transmission of VL—keeping the effective reproduction number above 1—as the average number of secondary VL cases (the main drivers of transmission) generated by each VL/PKDL case is typically less than 1 (Fig. 4*C*). The times after onset of symptoms in the infector at which secondary VL cases become infected are typically longer for PKDL infectors than for VL infectors (Fig. 4*B*), due to their longer durations of infection and generally lower infectiousness, so there is greater opportunity to intervene to prevent onward transmission from PKDL cases. Model simulations suggest that incidence of VL could be reduced by faster detection and treatment of PKDL cases. Depending on the relative prevalence of VL and PKDL, the reduction could be anywhere in the range of 9 to 17% if the average duration of PKDL infectiousness were halved and 25 to 46% if PKDL were completely prevented.

The spatiotemporal patterns of transmission inferred from reconstructing the transmission tree suggest that infection makes both short and long jumps in space within each infection generation (*SI Appendix*, Fig. S15). This is consistent with findings from a spatial analysis of occurrence of VL cases around index cases in Muzaffarpur, Bihar, India (38), which found a combination of short and long distances (from tens to hundreds of meters) from the closest index case for secondary VL cases diagnosed close together in time. The inferred transmission distances are also consistent with limited available data on the flight range of the *P. argentipes* sandfly vector, which suggests a short-term (0.5 to 2.5 d) flight range of around 300 m (39), and with the flight range of fed females of a species in the same genus of a few hundred meters (up to a maximum of nearly 1 km) (40). Considering that index cases are often detected after a longer delay than subsequent cases and there will be some delay in mounting reactive interventions, such as active case detection and/or targeted IRS around the index case(s), interventions will need to be applied in a large radius (up to 500 m) around index cases to be confident of capturing all secondary cases and limiting transmission.

Our results demonstrate the importance of accounting for spatial clustering of infection and disease when modeling VL transmission. Previous VL transmission dynamic models (23, 41) have significantly overestimated the relative contribution of asymptomatic infection to transmission (as up to 80%), despite assuming asymptomatic individuals are only 1 to 3% as infectious as VL cases, by treating the population as homogeneously mixing, such that all asymptomatic individuals can infect all susceptible individuals via sandflies. In reality, asymptomatic individuals do not mix homogeneously with susceptible individuals as they are generally clustered together around or near VL cases (25, 28), who are much more infectious and therefore more likely to infect susceptible individuals around them, even if they are outnumbered by asymptomatic individuals. Asymptomatic infection also leads to immunity and therefore local depletion of susceptible individuals around infectious individuals. Hence, for the same relative infectiousness, the contribution of asymptomatic individuals to transmission is much lower when spatial heterogeneity is taken into account.

The spatiotemporal data on incidence and duration of symptomatic infection used in this study provided insufficient information to estimate the relative infectiousness of asymptomatically and presymptomatically infected individuals, so we tested the sensitivity of model parameter estimates to the uncertainty in their estimated infectiousness from a xenodiagnostic study in India (32) and found high consistency in all but the spatial transmission rate constant. Although the failure of asymptomatic individuals to infect sandflies in the Indian xenodiagnosis study seems to suggest that they are not infectious (32), historical (13, 42) and experimental (43) data show that provision of a second blood meal and optimal timing of sandfly examination are critical to maximizing xenodiagnostic sensitivity. These data suggest that recent xenodiagnosis studies (12, 32), in which dissection occurred within 5 d of a single blood meal, may underestimate the potential infectiousness of symptomatic and asymptomatic infected individuals. Occurrence of VL in isolated regions where there are asymptomatically infected individuals, but virtually no reported VL cases (27, 33), also seems to suggest that asymptomatic individuals may occasionally generate VL cases. However, it is also possible that some individuals who developed VL during the study went undiagnosed and untreated and that we have inferred transmissions from asymptomatic individuals in locations where cases were missed. The potential role of underreporting will be investigated in future work.

The analysis presented here is not without limitations. As can be seen from the model simulations (*SI Appendix*, Fig. S23), the model is not able to capture the full spatiotemporal heterogeneity in the observed VL incidence when fitted to the data from the whole study area, as it underestimates the number of cases in higher-incidence paras (e.g., paras 1, 4, and 12). There are various possible reasons why the incidence in these paras might have been higher, including higher sandfly density, lower initial levels of immunity, variation in infectiousness between cases and within individuals over time, dose dependence in transmission [whereby flies infected by VL cases are more likely to create VL cases than flies infected by asymptomatic individuals (22)], and variation in other unobserved risk factors (such as bed net use). It was not possible to include sandflies explicitly in the model due to an absence of data on sandfly abundance and gaps in understanding of *P. argentipes* bionomics (10). We were unable to incorporate variation in infectiousness between individuals in the same disease state and over time within disease states due to the relatively limited xenodiagnostic data available on infectiousness of VL and PKDL and lack of data on variation in infectiousness of individuals over time (e.g., from serial parasite load measurements or serial xenodiagnosis). We were also not able to consider the role of HIV-VL coinfected individuals in transmission as there were no data on HIV infection in the study population, but other data suggest they may contribute significantly with prevalences of HIV coinfection of up to 6% in India (44) and higher infectiousness toward sandflies (45). Further laboratory and field studies are needed to quantify these sources of heterogeneity to be able to parameterize variation in transmission intensity between locations.

Another limitation of our analysis is that it does not account for the fact that some VL cases infected before the end of the study may not have developed symptoms until after the study finished and therefore not been observed. Adapting our MCMC algorithm to infer infection times of such cases is nontrivial and would require incorporating reversible jumps for adding and removing “hidden” infections (4), so we defer this to future work. Our estimates of the effective reproduction number toward the end of the study and in particular the contribution of PKDL cases to transmission as the main drivers of transmission at the end of the epidemic are thus likely to be biased downward. However, our approach does account for unobserved asymptomatic infections up to the end of the study and these constitute the vast majority (∼85%) of infections, so the bias in the effective reproduction number is likely to be relatively small. There were also only seven VL cases with onset in 2010, all of whom had onset before May, despite intensive follow-up to the end of December 2010, suggesting that transmission had substantially declined by 2010 and that the number of right-censored VL onsets may have been small. The overall contribution of PKDL to transmission, and therefore the potential impact of PKDL control, may still be underestimated, however, as 51 PKDL cases (27%) were untreated and still had unresolved lesions in December 2010, so may have infected other individuals after the end of the study.

We cannot discount the possibility of inaccuracy in our estimates due to recall bias, given some data were collected retrospectively and complete house-to-house searches were conducted only annually. However, the villages in the study area were visited continuously on a roving schedule over the prospective part of the study and participants were encouraged to self-report lesions and febrile illness (8), which should have mitigated some of this bias.

Despite these limitations, our analysis provides unique insights into how visceral leishmaniasis spreads in space and time and the role played by PKDL and asymptomatic infection in this process. We have developed a MCMC data augmentation framework to account for the endemic nature of the disease and high proportion of asymptomatic infection and used it to generate quantitative estimates for guiding targeted interventions around VL and PKDL cases. In future work we will predict the impact of different spatiotemporally targeted interventions on VL incidence using the simulation model developed here.

## Materials and Methods

### Data Collection.

The data used in this study were collected in a highly VL-endemic community in Fulbaria Upazila, Mymensingh district, Bangladesh, through a combination of four household surveys conducted between July 2007 and December 2010 and continuous follow-up by fieldworkers (for full details of the study protocol and case definitions see refs. 8 and 30). Each survey consisted of a complete house-to-house search for new (and past, in the case of the baseline survey) VL and PKDL cases over the whole study area, along with a census update to record any births, deaths, and migration into/out of/within the study area. A total of 138 of the 190 PKDL cases identified during the study were examined by an experienced physician to determine lesion extent and severity (see *SI Appendix* for further details). The global positioning system (GPS) coordinates of all households were recorded using a Garmin 76 GPS receiver.

### Transmission Model.

We developed a discrete-time individual-level spatial kernel transmission model for VL by extending our previous individual-level model (46) to explicitly include asymptomatic infection and PKDL. In the model, the infection pressure on susceptible individual i in month t is given by the sum of the individual infection pressures on the individual from surrounding infectious individuals (j∈Inf(t)), which are a function of their distance $d_{ij}$ from i and their relative infectiousness (compared with VL cases) $h_{j}\left(t\right)$, plus a background transmission rate ϵ to account for unexplained infections,

$$\lambda_{i}\left(t\right)=\underset{j\in Inf\left(t\right)}{\sum }\left(\beta K\left(d_{ij}\right)+\delta 1_{ij}\right)h_{j}\left(t\right)+\epsilon ,$$ [1]

where $K\left(d\right)\propto e^{-d/\alpha }$ is the spatial kernel function that determines how transmission risk decreases with distance (with distance decay rate 1/α), β is the spatial transmission rate constant, δ is the extra within-household transmission rate, and $1_{ij}$ is an indicator function that is 1 if i and j share the same household and 0 otherwise. A proportion $p_{I}$ of infections lead to VL following a negative-binomially distributed NB(r,p) incubation period, while the remaining infections are asymptomatic with geometric $\text{Geom}\left(p_{2}\right)$ duration. We use $p_{I}=0.15$, r=3, and $p_{2}=1/5$ based on previous analyses (29, 46) and estimate p along with the transmission parameters.

We assume lifelong immunity for individuals who recover from infection, regardless of whether they have recovered from VL, PKDL, or asymptomatic infection. While there is some uncertainty about whether individuals can be reinfected, particularly asymptomatically infected individuals, available evidence suggests that repeat episodes of VL are relatively rare and are due to relapse and not reinfection (47) and that in highly endemic settings a high proportion of asymptomatically infected individuals develop long-term protective cell-mediated immunity following infection (24, 26). This assumption is therefore not unrealistic and makes it feasible to infer the model parameters and missing data, which would be considerably more challenging if it was necessary to account for the possibility of multiple infections for asymptomatic individuals.

We assume individuals’ relative infectiousnesses $h_{j}\left(t\right)$ remain constant within each infection state, and parameterize those of VL and PKDL cases using data from a recent xenodiagnosis study in Bangladesh (12), and those of asymptomatic and presymptomatic individuals based on an estimate from a xenodiagnosis study in India that the probability of an asymptomatic individual infecting a sandfly is at most 2.3% (32) and estimates from previous modeling studies (23, 41). Given the uncertainty in the infectiousness of asymptomatic and presymptomatic individuals and the absence of experimental data on their infectiousness relative to each other, we assume they are equally infectious and test the sensitivity of the model parameter estimates to values of their infectiousness (relative to VL cases) of 0 to 2% (*SI Appendix*). We also compare the fit of models without and with additional within-household transmission (δ=0 vs δ>0) using a version of the deviance information criterion designed for latent variable models (*SI Appendix*).

### Bayesian Data Augmentation.

We estimated the parameters in the transmission model, θ=(β,α,ϵ,δ,p), the unobserved infection times of VL cases and infection and recovery (seroreversion) times of asymptomatic individuals, and individuals’ unobserved initial statuses by sampling from the joint posterior distribution of θ and the missing data X given the observed data Y (months of birth, migration, and death; VL and PKDL onset and recovery times; etc.), P(θ,X|Y)∝L(θ;Y,X)P(θ), where L(θ;Y,X) denotes the complete data likelihood and P(θ) is the prior distribution for θ, using a Bayesian data augmentation framework (see *SI Appendix* for full details). MCMC methods were used to obtain the joint posterior distribution by iteratively sampling from the posterior distribution of the parameters given the observed data and current value of the missing data, P(θ|Y,X), and the posterior distribution of the missing data given the observed data and the current values of the parameters, P(X|Y,θ). Relatively uninformative gamma distributions were used for the priors for the transmission parameters (β, α, ϵ, and δ), and a relatively informative conjugate beta prior was used for the incubation period distribution parameter p based on a previous estimate of the mean incubation period and its uncertainty (29) (see *SI Appendix* for further details). To validate the inference procedure we simulated data for part of the study area using known parameter values and verified that the MCMC algorithm could recover the true parameter values and unobserved data (*SI Appendix*). Code is available online at https://github.com/LloydChapman/VLSpatiotemporalModelling.

Once the posterior distribution of the parameters and missing data was obtained from the MCMC, 1,000 samples were drawn from the posterior distribution and the posterior predictive distributions of infection sources for all infectees derived for each sample. These were used to draw an infector for each infectee to reconstruct the transmission tree. Thus we obtained a set of 1,000 possible transmission trees that accounted for uncertainty in the parameter values, infection times, infection sources, and individuals’ initial statuses. The mean distance from each infector to his/her infectees and time from his/her onset to the infections of his/her infectees were calculated for each tree and then averaged over all trees in which that individual was an infector to obtain distributions of mean distances and times to infectees across all infectors (Fig. 4 *A* and *B*). The posterior predictive distributions of infection sources were also used to estimate the number of secondary infections for each asymptomatic individual, VL case, and PKDL case (Fig. 5 *A* and *B* and *SI Appendix*, Fig. S22) and the time-dependent effective reproduction number (Fig. 5*C*).

### Model Simulations.

We implemented a simulation version of the transmission model (full details in *SI Appendix*) to assess the ability of the model to reproduce the observed data and to investigate the counterfactual impact of different hypothetical interventions against PKDL on VL incidence. One hundred samples of the parameters and individuals’ infection statuses in December 2002 were drawn from the posterior distribution obtained from the MCMC and 100 simulations of the model were run for each sample starting from January 2003 (at which point all but one of the paras had had at least one VL case since January 2002), to give 10,000 realizations of the epidemic under “normal” interventions. This process was then repeated with PKDL infectiousness set to zero (to simulate no development of PKDL) and then again with the mean duration of PKDL infectiousness halved (to simulate more rapid detection and treatment of PKDL), and the percentage difference in the total number of cases in each “alternative-intervention” simulation from that in each “normal-intervention” simulation was calculated.

### Ethical Approval.

This study was approved by the institutional review boards of the International Center for Diarrheal Disease Research, Bangladesh (protocol 2007-003) and the Centers for Disease Control and Prevention (protocol 5065), and informed consent was obtained from all participants or parents/guardians in the case of children.

## Supplementary Material

Supplementary File

Supplementary File

Supplementary File

## Data Availability

Code is available online at https://github.com/LloydChapman/VLSpatiotemporalModelling. The analyzed data contain personally identifiable information and cannot be made publicly available. Individuals who wish to access the analyzed data should contact caryn.bern2@ucsf.edu.
