# Supplementary material for: Inferring transmission trees to guide targeting of interventions against visceral leishmaniasis and post–kala-azar dermal leishmaniasis
Source: Proc Natl Acad Sci U S A. 2020 Sep 24;117(41):25742–50. doi: 10.1073/pnas.2002731117 (PMC7568327; doi:10.1073/pnas.2002731117)
Supplement: Supplementary File [file pnas.2002731117.sapp.pdf]

PNAS
www.pnas.org

Supplementary Information for

Inferring transmission trees to guide targeting of interventions against visceral leishmaniasis and post-kala-azar dermal leishmaniasis

Lloyd A. C. Chapman, Simon E. F. Spencer, Timothy M. Pollington, Chris P. Jewell, Dinesh Mondal, Jorge Alvar, T. Déirdre Hollingsworth, Mary M. Cameron, Caryn Bern, Graham F. Medley

Lloyd A. C. Chapman.
E-mail: lloyd.chapman@ucsf.edu

This PDF file includes:

Supplementary text
Figs. S1 to S23
Tables S1 to S7
Captions for Movies S1 to S2
References for SI reference citations

Other supplementary materials for this manuscript include the following:

Movies S1 to S2

Supporting Information Text

This file provides further information on the spatiotemporal transmission model and Bayesian inference framework described in the *Materials and Methods* section in the main text, including a full description of the model, the formula for the likelihood of the data given the model, details of, and additional output from, the Markov Chain Monte Carlo (MCMC) algorithm and model simulations, and validation of the MCMC algorithm.

1. Model Description

The model used in this paper (Fig. S1) is an extension of our previously published individual-level spatiotemporal SEIR model of visceral leishmaniasis (VL) transmission (1) to explicitly include asymptomatic infection and post-kala-azar dermal leishmaniasis (PKDL), and account for unobserved initial infection statuses and migration of individuals. We measure time in units of months, with $t = 1$ corresponding to the start of the study (January 2002) and $t = T = 108$ the end of the study (December 2010), and label individuals who developed VL symptoms between January 2002 and December 2010 by $i = 1, 2, \ldots, n_I$ ($n_I = 1018$) and the remainder of the population by $i = n_I + 1, n_I + 2, \ldots, n$ ($n = 25506$). Between January 2002 and December 2010, 725 individuals relocated between different households within the study area, which we account for by including a second observation for these individuals in their second household. These internal migrators are essentially treated as extra individuals for the purpose of calculating pairwise distances and transmission rates. However, their infection status is updated in such a way that it is consistent across the two observations (e.g. if they are asymptomatically infected in the period of the first observation and do not recover before relocating, their asymptomatic infection status is carried over to the second observation). We denote the vectors of event times for individuals $i = 1, \ldots, n$ as follows:

- birth: $\mathbf{B} = (B_i)$,
- immigration and emigration (either within, or from/to outside, the study area): $\mathbf{IM} = (IM_i)$ and $\mathbf{EM} = (EM_i)$,
- asymptomatic infection: $\mathbf{A} = (A_i)$,
- pre-symptomatic infection: $\mathbf{E} = (E_i)$,
- VL onset: $\mathbf{I} = (I_i)$,
- recovery, from VL through treatment or naturally from asymptomatic infection: $\mathbf{R} = (R_i)$,
- VL relapse: $\mathbf{I}_R = (I_{Ri})$,
- VL relapse treatment: $\mathbf{R}_R = (R_{Ri})$,
- temporary recovery to dormant infection prior to PKDL or VL relapse: $\mathbf{D} = (D_i)$,
- PKDL onset: $\mathbf{P} = (P_i)$,
- PKDL resolution: $\mathbf{R}_P = (R_{Pi})$,
- death: $\mathbf{M} = (M_i)$.

In any particular month t individuals can be in one of the 7 states shown in Fig. S1A:

- susceptible: $\mathcal{S}(t) := \{i : \max(B_i, IM_i) \leq t < \min(EM_i, A_i, E_i, M_i)\}$
- asymptomatically infected (and potentially infectious): $\mathcal{A}(t) := \{i : A_i \leq t < \min(EM_i, R_i, D_i, M_i)\}$
- pre-symptomatically infected (and potentially infectious): $\mathcal{E}(t) := \{i : E_i \leq t < I_i\}$
- symptomatic VL: $\mathcal{I}(t) := \{i : (\max(I_i, IM_i) \leq t < \min(EM_i, R_i, D_i, M_i)) \text{ or } (I_{Ri} \leq t < \min(R_{Ri}, D_i, M_i))\}$
- dormantly infected (i.e. treated for VL or recovered from asymptomatic infection but with subsequent VL relapse or PKDL): $\mathcal{D}(t) := \{i : D_i \leq t < \min(I_{Ri}, P_i)\}$
- symptomatic PKDL (i.e. visible skin lesions): $\mathcal{P}(t) := \{i : \max(P_i, IM_i) \leq t < \min(EM_i, R_i^P, M_i)\}$
- recovered (i.e. treated for primary VL, VL relapse or PKDL, or self-resolved from PKDL, or recovered from asymptomatic infection): $\mathcal{R}(t) = \{i : (R_i \leq t < \min(EM_i, M_i)) \text{ or } (R_{Ri} \leq t < \min(EM_i, M_i)) \text{ or } (R_{Pi} \leq t < \min(EM_i, M_i))\}$.

Upon infection, individuals either develop pre-symptomatic infection with probability p_I or asymptomatic infection with probability $1 - p_I$ (see Table S2 for values of fixed parameters used in the model). Pre-symptomatic individuals progress to symptomatic VL following a variable incubation period, and then upon treatment to recovery, or dormant infection if they later relapse or develop PKDL. VL cases that relapse either return to dormant infection or progress to recovery once re-treated. Asymptomatically infected individuals either recover naturally or very occasionally progress to dormant infection and subsequent PKDL. PKDL cases can either resolve following treatment or naturally, whereupon they enter the recovered class. We assume that recovered individuals do not return to being susceptible, i.e. cannot be reinfected, irrespective of whether they have recovered from VL, PKDL or asymptomatic infection. It is still uncertain whether individuals can become reinfected with *L. donovani* parasites, particularly asymptomatically infected individuals. Most previous modelling studies have assumed or concluded that individuals can be reinfected (2–4), but have not accounted for spatial variation in transmission and available evidence suggests that repeat episodes of VL are relatively rare and are due to relapse not reinfection (5–7), and that in

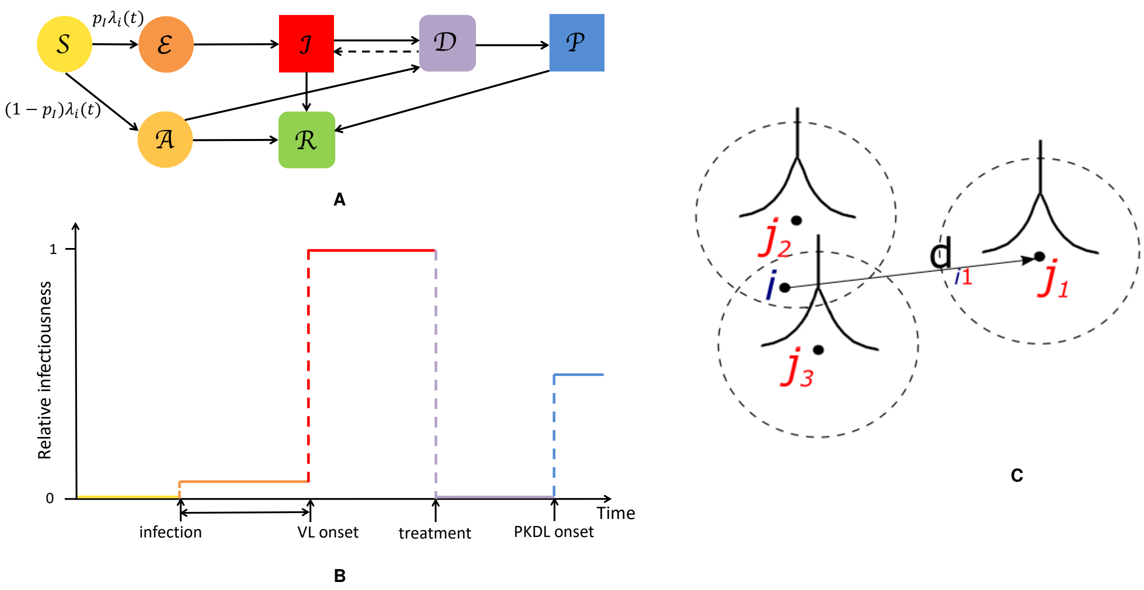

Fig. S1. Individual-level spatiotemporal transmission model. (*A*) States in the model ($\mathcal{S}$ = susceptible, $\mathcal{A}$ = asymptomatically infected, $\mathcal{E}$ = pre-symptomatically infected, $\mathcal{I}$ = symptomatic VL, $\mathcal{D}$ = dormantly infected, $\mathcal{P}$ = PKDL and $\mathcal{R}$ = recovered). Squares represent fully observed states, rounded-edge squares partially observed states, and circles unobserved states. Dashed arrow shows VL relapse from dormant infection. p_I = probability of symptomatic infection, $\lambda_i(t)$ = total infection pressure on susceptible individual i at time t. (*B*) Relative infectiousness over time of individual passing through top pathway (symptomatic infection) in (A). Diagram not to scale and shown assuming that pre-symptomatic individuals are infectious and VL case developed macular PKDL. (*C*) The total infection pressure $\lambda_i(t)$ on a susceptible individual i at time t is the sum of the individual infection pressures on them from infectious individuals around them (here j_1, j_2 and j_3), which are a function of how far away they are (depicted by the curves), the time since their infections and whether their infection is symptomatic or asymptomatic. d_{i1} = distance between i and j_1.

highly endemic settings a high proportion of asymptomatically infected individuals develop long-term protective cell-mediated immunity following infection (8, 9).

For each individual i in the study population, we define $V_i = \max(0, B_i, IM_i)$ and $W_i = \min(T+1, EM_i, M_i)$ as their times of entry into and exit from the study area respectively. All non-symptomatic individuals (individuals without any VL or PKDL symptoms before or during the study) who were born or entered the study area after January 2002 are assumed to have been susceptible upon entry.

A. Pairwise infection pressures. Susceptible individuals become infected either from pre-symptomatically infected individuals, from symptomatic VL cases, PKDL cases, asymptomatic individuals, or 'background' (unexplained) transmission. The 'infection pressure' exerted by an infected individual j on a susceptible individual i at time t is given by:

$$\lambda_{ij}(t) = (\beta K(d_{ij}) + \delta \mathbb{1}_{ij}) h_j(t), \quad i \in \mathcal{S}(t),\ j \in \mathcal{E}(t) \cup \mathcal{A}(t) \cup \mathcal{I}(t) \cup \mathcal{P}(t), \tag{S1}$$

where β is the rate constant for spatial transmission between infected and susceptible individuals; $K(d_{ij})$ is the spatial kernel function that scales the infection pressure by the distance d_{ij} between individuals i and j; δ (≥ 0) is a rate constant for additional within-household transmission; $\mathbb{1}_{ij}$ is an indicator function for individuals living in the same household, i.e.

$$\mathbb{1}_{ij} = \begin{cases} 1, & \text{if } i \text{ and } j \text{ are in the same household,} \\ 0, & \text{otherwise;} \end{cases}$$

and $h_j(t)$ is the infectiousness of j at time t. Individuals are assumed to be stationary in their households when transmission to and from sandflies occurs (since the vast majority of sandfly biting occurs at night when individuals are asleep in, or directly outside, their homes (10–14)), so that the distances between them d_{ij} are fixed when transmission takes place. However, the changes in the distances between individuals that occur when individuals migrate within the study area is incorporated into the model (by calculating the distances between migrators and all other individuals both when they are in their first household and when they are in their second household).

Since we found little difference in the goodness of fit of the model (based on the Deviance Information Criterion (DIC)) between an exponentially decaying spatial kernel and a Cauchy-type kernel in our previous study (1) (the exponential kernel gave a marginally better fit), we use the exponential kernel here:

$$K(d_{ij}) = K_0 e^{-d_{ij}/\alpha}, \quad \text{for } i, j = 1, \ldots, n. \tag{S2}$$

where $1/\alpha$ is the distance decay rate (per m) in transmission risk (so smaller values of α correspond to a faster decrease in risk with distance), and K_0 is a normalisation constant, defined such that

$$\sum_{i=1}^{n} \sum_{j=1}^{n} K(d_{ij}) = n,$$

which reduces correlation between α and β and thus improves the mixing of the MCMC algorithm (§2B.4). However, unlike in our previous study, we allow the possibility of transmission between individuals in different paras (hamlets). The reason for this is that surveying of neighbouring paras was more complete in the two clusters of paras in the present study, and thus some minimum inter-para household distances were less than intra-para household distances and within the estimated short-term flight range of *P. argentipes* sandflies (a few hundred metres (15–17)), so transmission between neighbouring surveyed paras may have occurred.

B. Infectiousness over time. There is relatively little data available on the infectiousness of individuals in different *L. donovani* infection states over time. We therefore assume that the infectiousness of individuals remains constant within each state and individuals in the same state have the same probability of passing on infection, such that an individual's infectiousness over time takes the form of a step function (Fig. S1B). The relative infectiousness of asymptomatic individuals compared to VL cases, h_4, is assumed to take a fixed value between 0 and 0.02 based on results from xenodiagnosis studies in which 0 of 183 asymptomatically infected individuals infected sandflies (95% confidence interval for transmission probability: 0–0.023) (18), and estimates from previous modelling studies (3, 4, 19) (Table S1). We test different values of h_4 to assess the sensitivity of the other parameter estimates to the assumed asymptomatic infectiousness. In the absence of data on the relative infectiousness of pre-symptomatic individuals and asymptomatic individuals, we take the relative infectiousness of pre-symptomatic individuals, h_0, to be the same as that of asymptomatic individuals (i.e. $h_0 = h_4$).

One-hundred and thirty-eight of the 190 PKDL cases underwent one or more examinations by a trained physician to determine the type and extent of their lesions (Table S1). Data from a recent xenodiagnosis study in Bangladesh (20) on 47 PKDL patients (21 nodular and 26 macular/papular) and 15 VL patients is used to assign infectiousness to these cases according to their lesion type (macular/papular, plaque, or nodular) (Table S1). The results of the xenodiagnosis study suggest that infectiousness of PKDL increases with lesion severity and macular/papular PKDL cases are less infectious towards sandflies than VL cases but nodular PKDL cases more so. As plaques were not treated as a separate lesion type in the study, but are intermediate in severity between macular/papular lesions and nodular lesions (21), a value halfway between the infectiousness of macular/papular PKDL cases and nodular PKDL cases is assigned to these individuals. The 52 PKDL cases that were not physically examined during the study are assigned an average of the infectiousnesses of the different lesion types (weighted by frequency amongst the examined cases). Although it is not known for certain whether treated VL cases who subsequently relapse or develop PKDL are uninfectious following treatment, we assume dormantly infected individuals are uninfectious, based on rapid decreases in parasitaemia observed in VL cases following the start of treatment (22, 23)). We assume that relapse cases are as infectious upon relapse as in their first clinical episode, as relapse appears to be associated with resurgence in parasitaemia to high parasite loads (23, 24). Over half (100/190=53%) of the PKDL cases did not receive treatment, and of these 49 self-resolved in a median time of 20 months (interquartile range (IQR) 14–32 months). These cases are assumed to have remained infectious until their lesions resolved, while treated PKDL cases are assumed to have stopped being infectious once their treatment commenced (25).

Table S1. Infectiousnesses of different infection states relative to visceral leishmaniasis (VL)

Infection state	Proportion in physical examinations	Proportion positive in xenodiagnosis experiments*	Relative infectiousness	Source
Pre-symptomatic	-	-	$h_0 = 0$–0.02	Assumed ($h_0 = h_4$)
VL (primary/relapse)	-	10/15 (67%)	1	Reference value
PKDL				
-macular/papular	101/138 (73%)	9/26 (35%)	$h_1 = (9/26)/(10/15) = 0.52$	(20, 21)
-plaque	31/138 (22%)	-	$h_2 = ((9/26 + 18/21)/2)/(10/15) = 0.90$	(21)
-nodular	6/138 (4%)	18/21 (86%)	$h_3 = (18/21)/(10/15) = 1.29$	(20, 21)
-unexamined	-	-	$h_u = (101/138 \times 0.52) + (31/138 \times 0.90) + (6/138 \times 1.29) = 0.64$	Assumed
Asymptomatic	-	-	$h_4 = 0$–0.02	(4, 18, 19)

*Proportion who infected flies based on positive microscopy and/or qPCR on fed flies.

Thus, individual j's infectiousness at time t is given by

$$h_j(t) = \begin{cases} h_0, & \text{if } j \in \mathcal{E}(t), \\ 1, & \text{if } j \in \mathcal{I}(t), \\ h_1, & \text{if } j \in \mathcal{P}(t) \text{ \& } j \text{ has macular/papular lesions,} \\ h_2, & \text{if } j \in \mathcal{P}(t) \text{ \& } j \text{ has plaques,} \\ h_3, & \text{if } j \in \mathcal{P}(t) \text{ \& } j \text{ has nodular lesions,} \\ h_u, & \text{if } j \in \mathcal{P}(t) \text{ \& } j \text{ has unknown PKDL type,} \\ h_4, & \text{if } j \in \mathcal{A}(t) \\ 0, & \text{if } j \in \mathcal{S}(t) \cup \mathcal{D}(t) \cup \mathcal{R}(t). \end{cases} \quad \text{[S3]}$$

C. Incubation period. Following previous work (1), we model the incubation period as negative binomially distributed $\text{NB}(r,p)$ with fixed shape parameter $r=3$ and 'success' probability parameter p, and support starting at 1 (such that the minimum incubation period is 1 month), i.e. via the following probability mass function (PMF):

$$f_E(x)=\mathbb{P}(IP_j=x)=\frac{\Gamma(r+x-1)}{(x-1)!\Gamma(r)}p^r(1-p)^{x-1},\quad x\in\{1,2,3,\dots\} \tag{S4}$$

We estimate p in the MCMC algorithm for inferring the model parameters and missing data (see §2).

D. VL onset-to-treatment time distribution. Several VL cases with onset before 2002 have missing symptom onset and/or treatment times (only their onset year is recorded), and may therefore have been infectious at the start of the study period. In order to be able to infer the onset-to-treatment times of these cases, $OT'_j=\min(R'_j,D'_j)-I'_j$ (where primed variables are the missing counterparts of the observed variables), in the MCMC algorithm (see §2) we model the onset-to-treatment time distribution as a negative binomial distribution $\text{NB}(r_1,p_1)$ (Table S2) and fit to the onset-to-treatment times of all VL cases for whom both onset and treatment times were recorded (Fig. S2A):

$$f_I(x)=\mathbb{P}(OT'_j=x)=\frac{\Gamma(r_1+x-1)}{(x-1)!\Gamma(r_1)}p_1^{r_1}(1-p_1)^{x-1},\quad x\in\{1,2,3,\dots\}, \tag{S5}$$

to obtain $r_1=1.34$ and $p_1=0.38$ (corresponding to a mean onset-to-treatment time of 3.2 months).

E. Asymptomatic infection duration. Neither the infection or recovery time of asymptomatically infected individuals is observed, so we need to specify a distribution for the asymptomatic infection duration in order to infer their recovery times. Based on a previous multi-state Markov model of the natural history of VL (26), in which it was assumed that the duration of asymptomatic infection is exponentially distributed and its mean was estimated as approximately 5 months, we assume the asymptomatic infection duration, $AIP_j=R_j-A_j$, follows a geometric distribution (the discrete analog of the exponential distribution) $\text{Geom}(p_2)$, with a minimum duration of 1 month and a mean of 5 months ($p_2=\frac{1}{5}$):

$$f_A(x)=\mathbb{P}(AIP_j=x)=(1-p_2)^{x-1}p_2,\quad x\in\{1,2,3,\dots\} \tag{S6}$$

The choice of the geometric distribution is partly motivated by the fact that it is a memoryless distribution, i.e. $\mathbb{P}(AIP_j>s+t|AIP_j>s)=\mathbb{P}(AIP_j>t)$. This simplifies the estimation of recovery times for initially asymptomatically infected individuals (see §1I) in the MCMC algorithm as it means that the probability that an initially asymptomatically infected individual remains infected for a further t months is the same as if they were infected in month 0 regardless of when they were infected.

F. Dormant infection duration. Sixteen (8.4%) of the 190 PKDL cases reported no prior history of VL, and are assumed to have been previously asymptomatically infected. We assume that their duration of dormant infection prior to PKDL and that of VL cases who develop PKDL follow the same negative binomial distribution, $\text{NB}(r_3,p_3)$, and estimate r_3 and p_3 by fitting to the observed VL-treatment-to-PKDL-onset times by maximum likelihood estimation (Fig. S2B). Unlike for the incubation period, we take 0 months to be the minimum duration of dormant infection, since two VL cases developed PKDL in the same month as they were treated for VL, and one had simultaneous VL and PKDL. Thus the PMF is:

$$f_D(x)=\frac{\Gamma(r_3+x)}{x!\Gamma(r_3)}p_3^{r_3}(1-p_3)^x,\quad x\in\{0,1,2,3,\dots\}, \tag{S7}$$

where $r_3=1.73$ and $p_3=0.064$ (corresponding to a mean duration of dormant infection of 25 months).

G. Time to relapse and relapse duration. Forty-five VL cases suffered treatment failure or relapse during the study. Of these, 16 were treated with miltefosine and 29 with sodium stibogluconate (SSG). Of those treated with miltefosine, 15 were treated with counterfeit drug in 2008 (27) and all but one of these cases reported no gap between the start of treatment and recurrence of symptoms (the other case reported a gap of 30 days). We assume that individuals without any gap between treatment and symptom recurrence suffered treatment failures, and that their infectiousness continued for 1 month following the start of treatment until they were treated with SSG. The gap between the start of treatment and new symptoms occurring was recorded for 7 of the cases originally treated with SSG or miltefosine from a clinical trial, and was non-zero in all cases. We assume that all cases not recorded as having immediate recurrence of symptoms suffered VL relapse and that the time to relapse follows a geometric distribution $\text{Geom}(p_4)$ with PMF:

$$f_R(x)=(1-p_4)^{x-1}p_4,\quad x\in\{1,2,3,\dots\}, \tag{S8}$$

where fitting to the recorded gaps gives $p_4=0.13$ (corresponding to a mean time to relapse of 7.9 months). Relapse cases are assumed to be uninfectious from their treatment month to their relapse time and their duration of symptoms upon relapse is assumed to follow the same distribution as the onset-to-treatment time for a first VL episode (Eq. (S5)). We assume all relapse cases were treated for relapse before the end of the study, since the latest treatment time for primary VL in a case that subsequently relapsed was April 2009.

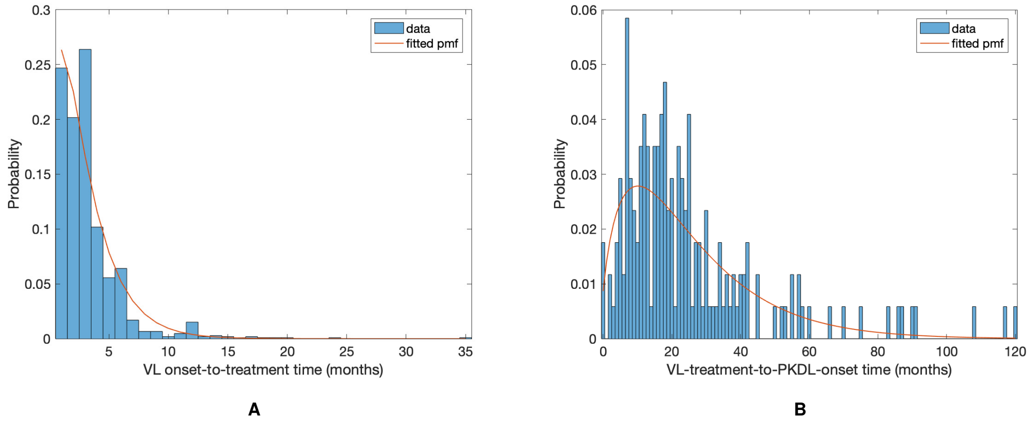

Fig. S2. Observed and fitted negative binomial distributions of (*A*) VL onset-to-treatment time and (*B*) VL-treatment-to-PKDL-onset time.

H. Infection pressure. The total infection pressure on susceptible individual i at time t is given by the sum of the infection pressures on them from all infectious individuals (VL cases, PKDL cases, pre-symptomatic individuals and asymptomatic individuals) at time t in Eq. (S1) (see Fig. S1C) plus a constant background transmission rate ϵ to account for unexplained infections due to non-explicitly modelled factors (e.g. due to short-term movement of individuals):

$$\lambda_i(t) = \sum_{j \in Inf(t)} \lambda_{ij}(t) + \epsilon = \sum_{j \in Inf(t)} \Big((\beta K(d_{ij}) + \delta \mathbb{1}_{ij}) h_j(t) \Big) + \epsilon. \quad [S9]$$

where $Inf(t) = \mathcal{E}(t) \cup \mathcal{A}(t) \cup \mathcal{I}(t) \cup \mathcal{P}(t)$ is the set of all individuals infectious at time t. The transmission process is thus described by a discrete-time approximation to an inhomogeneous Poisson process with rate $\lambda(t) = \sum_{i \in \mathcal{S}(t)} \lambda_i(t)$ (28). The probability of susceptible individual i remaining susceptible in any particular month t is:

$$q_i(t) = e^{-\lambda_i(t-1)}, \quad i \in \mathcal{S}(t), \quad [S10]$$

while the probabilities of pre-symptomatic or asymptomatic infection in month t given susceptibility up to month $t-1$ are, respectively:

$$p_i^I(t) = 1 - e^{-p_I \lambda_i(t-1)}, \quad i \in \mathcal{S}(t-1), \quad [S11]$$

$$p_i^A(t) = 1 - e^{-(1-p_I)\lambda_i(t-1)}, \quad i \in \mathcal{S}(t-1). \quad [S12]$$

I. Model for initial status of non-symptomatic individuals. As there was transmission and VL in the population before the start of the study, individuals with no record of VL prior to 2002 may have been asymptomatically infected before the start of the study. Thus, the initial statuses of non-symptomatic individuals are censored and need to be estimated. Although the data on VL pre-2002 is likely incomplete, we adopt the simplifying assumption that any individual that had VL prior to 2002 is at least recorded as having had previous VL (even if their onset and treatment times are missing, imprecise or inaccurate), such that anyone in the rest of the population who was infected prior to 2002 could only have had asymptomatic infection. We also average over historical and spatial variation in the transmission rate, by assuming that the asymptomatic infection rate prior to 2002, λ_0, was constant. We assume that all 16 individuals who developed PKDL during the study period without prior VL were asymptomatically infected during the study rather than before (i.e. we ignore the possibility that such individuals were initially dormantly infected). The latter assumption is not unreasonable despite the estimated long duration of dormant infection, as the earliest PKDL onset amongst these individuals was in November 2005 (47 months into the study), and it is unlikely to significantly affect the results given the small number of such cases. With these assumptions we arrive at the simplified model for the initial status of non-symptomatic individuals shown in Fig. S3.

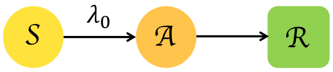

Fig. S3. Model for the initial statuses of non-symptomatic individuals, with a constant asymptomatic infection rate, λ_0.

The probabilities of each non-symptomatic individual initially present (i.e. with $V_j = 0$) being susceptible, asymptomatically infected, or recovered from asymptomatic infection at time $t = 0$ (p_{S_0}, p_{A_0}, p_{R_0}) can then be found by calculating the probability of avoiding asymptomatic infection in every month from their birth to the start of the study (Eq. (S13)), summing over the probabilities of being asymptomatically infected in one of the months between their birth and the start of the study

and recovering after the start of the study (Eq. (S14)), and summing over the probabilities of being asymptomatically infected in a month before the start of the study and recovering before the start of the study (Eq. (S15)), respectively:

$$p_{S_0}(a_j) := \mathbb{P}(A_j > 0, R_j > 0) = \prod_{t=-a_j+1}^{0} e^{-\lambda_0} = e^{-\lambda_0 a_j} \qquad \text{[S13]}$$

$$\begin{aligned} p_{A_0}(a_j) &:= \mathbb{P}(A_j \leq 0, R_j > 0) = \sum_{t=-a_j+1}^{0} e^{-\lambda_0(t+a_j-1)} \left(1-e^{-\lambda_0}\right) \mathbb{P}(R_j - A_j > -t) \\ &= \sum_{s=1}^{a_j} e^{-\lambda_0(s-1)} \left(1-e^{-\lambda_0}\right) (1-p_2)^{a_j-s} = \frac{\left(1-e^{-\lambda_0}\right)\left((1-p_2)^{a_j} - e^{-\lambda_0 a_j}\right)}{1-p_2-e^{-\lambda_0}} \end{aligned} \qquad \text{[S14]}$$

$$p_{R_0}(a_j) := \mathbb{P}(A_j < 0, R_j \leq 0) = 1 - p_{S_0}(a_j) - p_{A_0}(a_j) = 1 - e^{-\lambda_0 a_j} - \frac{\left(1-e^{-\lambda_0}\right)\left((1-p_2)^{a_j} - e^{-\lambda_0 a_j}\right)}{1-p_2-e^{-\lambda_0}} \qquad \text{[S15]}$$

where a_j is the age of individual j in months at $t = 0$. Since we assume that non-symptomatic individuals who are born, or who immigrate into the study area, after the start of the study (with $V_j > 0$) are susceptible, for notational convenience we define the probabilities for these individuals as $p_{S_0}(a_j) = 1$, $p_{A_0}(a_j) = p_{R_0}(a_j) = 0$.

We estimate the historical asymptomatic infection rate, λ_0, by fitting the model to age-prevalence data on leishmanin skin test (LST) positivity amongst non-symptomatic individuals from a cross-sectional survey of three of the study paras conducted in 2002 (8) (see Fig. S4). We assume that entering state $\mathcal{R}$ corresponds to becoming LST-positive, as LST positivity is a marker for durable, protective cell-mediated immunity against VL (8, 9), and estimate λ_0 by maximising the binomial likelihood:

$$L_{\mathrm{LST}}(\lambda_0) = \prod_{j=1}^{n_L} p_{R_0}(a_j) \prod_{j=n_L+1}^{n_{NS}} (1 - p_{R_0}(a_j)),$$

where $n_L = 479$ is the number of non-symptomatic individuals that were LST-positive out of $n_{NS} = 1399$ individuals tested. This gives $\lambda_0 = 0.0019\,\text{month}^{-1}$ (95% confidence interval 0.0017–0.0021 month^{-1}).

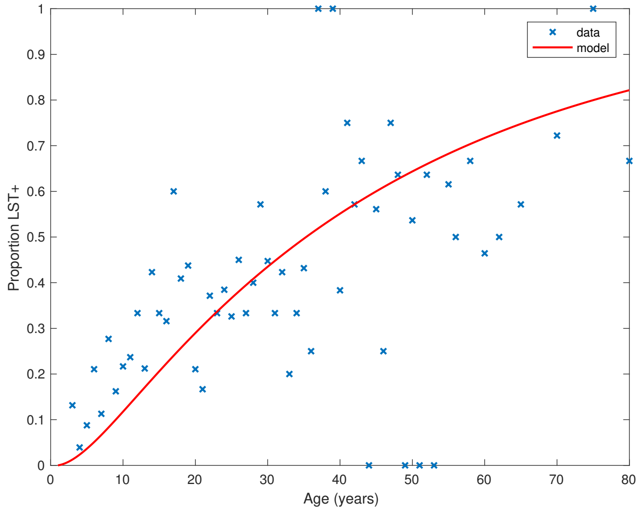

Fig. S4. Age-prevalence distribution of LST positivity among non-symptomatic individuals in three of the study paras in 2002 and fit of initial status model in Fig. S3.

J. Complete data likelihood. We assume that the end of the epidemic was observed from the point of view of VL and PKDL cases, i.e. every individual in the study population who eventually developed VL or PKDL did so within the observation period. Whilst it is likely that there was ongoing transmission beyond the end of the study in December 2010, this simplifying assumption should not introduce significant error based on the epidemic curves in Fig. 1 in the main text and the very low numbers of VL and PKDL cases with onset in the final months of the study.

As noted in the main text, there is a considerable amount of missing data, including the infection times of VL cases ($\mathbf{E}$) and the infection and recovery times of asymptomatic individuals ($\mathbf{A}$ and $\mathbf{R}_A$). So that we can specify the complete data likelihood — the likelihood of all events if all variables had been observed — and perform likelihood-based inference, we define the sets of all observed data and missing data as $\mathbf{Y} = (\mathbf{B}, \mathbf{IM}, \mathbf{EM}, \tilde{\mathbf{I}}, \tilde{\mathbf{R}}_I, \tilde{\mathbf{I}}_R, \mathbf{D}_I, \mathbf{P}, \mathbf{R}_P, \mathbf{M})$ and $\mathbf{X} = (\mathbf{A}, \mathbf{E}, \mathbf{R}_A, \mathbf{I}', \mathbf{R}_I', \mathbf{D}_A, \mathbf{I}_R', \mathbf{R}_R)$ respectively, where $\tilde{\mathbf{I}}$, $\tilde{\mathbf{R}}_I$ and $\tilde{\mathbf{I}}_R$ are the observed onset and recovery times of VL cases and relapse times of VL relapse cases; $\mathbf{I}'$, $\mathbf{R}_I'$ and $\mathbf{I}_R'$ are their missing counterparts; and $\mathbf{D}_I$ and $\mathbf{D}_A$ are the start times of dormant infection for VL cases and asymptomatic individuals.

Table S2. Values of fixed parameters used in the model

Parameter	Description	Value	Source
p_I	Proportion of infected individuals who develop VL	0.15	(26)
r	Shape parameter of negative binomial incubation period distribution	3	(1)
r_1	Shape parameter of negative binomial onset-to-treatment time distribution	1.34	Fitting to observed data
p_1	'Success' probability parameter of negative binomial onset-to-treatment time distribution	0.38	Fitting to observed data
p_2	Asymptomatic infection duration distribution parameter	0.2	(26)
r_3	Shape parameter for negative binomial dormant infection duration distribution	1.73	Fitting to observed data
p_3	'Success' probability parameter of negative binomial dormant infection duration distribution	0.064	Fitting to observed data
p_4	Treatment-to-relapse distribution parameter	0.13	Fitting to observed data
λ_0	Historical asymptomatic infection rate (month^{-1})	0.0019	Fitting to data on LST-positive prevalence in study area (8)
r_5	Shape parameter of negative binomial PKDL-onset-to-recovery time distribution*	1.18	Fitting to observed data
p_5	'Success' probability parameter of negative binomial PKDL-onset-to-recovery time distribution*	0.066	Fitting to observed data
p_P	Proportion of VL cases who develop PKDL*	0.17	Survival analysis in (21)
p_D	Proportion of asymptomatic individuals who develop PKDL*	0.0028	Estimated from observed incidence of PKDL amongst asymptomatic individuals

*Parameters only used in the simulations in §6 as they do not appear in the likelihood in Eq. (S16).

With these definitions, the complete data likelihood for the augmented data $\mathbf{Z} = (\mathbf{Y}, \mathbf{X})$ given the model parameters $\boldsymbol{\theta} = (\beta, \alpha, \epsilon, \delta, p)$ is composed of the products of the probabilities of all the different individual-level events over all months:

$$L(\boldsymbol{\theta};\mathbf{Z}) \propto \underbrace{\prod_{t=1}^{T}\prod_{j\in\mathcal{S}(t)} q_j(t)}_{L_1} \underbrace{\prod_{t=1}^{T}\prod_{j\in\{E_j=t,I_j>12\}} p_j^I(t)}_{L_2} \underbrace{\prod_{j=1}^{n_I} f_E(I_j-E_j)}_{L_3} \underbrace{\prod_{t=1}^{T}\prod_{j\in\{A_j=t\}} p_j^A(t)}_{L_4}$$
$$\times \underbrace{\prod_{j\in\{B_j\leq 0,E_j>12\}} p_{S_0}(a_j) \prod_{j\in\{B_j\leq 0,A_j>0,R_j>0\}} p_{S_0}(a_j) \prod_{j\in\{A_j=0,R_j>0\}} p_{A_0}(a_j) \prod_{j\in\{A_j=R_j=0\}} p_{R_0}(a_j)}_{L_5}$$
$$\times \prod_{j\in\{0\leq A_j\leq T,R_j\leq T\}} f_A(R_j-A_j) \prod_{j\in\{0\leq A_j\leq T,R_j>T\}} \big(1-F_A(T-A_j)\big) \prod_{j\in\{1\leq A_j\leq T,P_j\leq T\}} f_A(D_j-A_j)f_D(P_j-D_j)$$
$$\times \prod_{j\in\{I_j<T,R'_j\leq T\}} f_I(R'_j-I_j) \prod_{j\in\{I'_j<T,R'_j\leq T\}} f_I(R'_j-I'_j) \prod_{j\in\{1\leq I'_{Rj}\leq T\}} f_R(I'_{Rj}-R_j)f_I(R_{Rj}-I'_{Rj}). \quad \text{[S16]}$$

Here, L_1 is the overall probability of individuals avoiding infection (from Eq. (S10)) while subject to infection pressure from infectious individuals around them (Eq. (S9)), L_2 is the probability of all pre-symptomatic infections which occur (from Eq. (S11)), L_3 is the probability of the unobserved incubation periods of VL cases (from Eq. (S4)), L_4 is the probability of all unobserved asymptomatic infections which occur (from Eq. (S12)), L_5 is the probability of individuals' unobserved initial infection statuses according to the historical transmission rate (from Eqs (S13)–(S15)), and $F_A(x) = 1-(1-p_2)^x$ $(x \geq 1)$ is the cumulative distribution function of the asymptomatic infection period distribution (Eq. (S6)).

Given the length and variability of the incubation period, some VL cases with onset early in the study (i.e. in 2002) may have been infected before 2002. However, unlike in our previous work (1), we cannot assume that these cases arose due to background transmission, as there was not an extended period without VL cases directly before 2002. In fact, 413 individuals in the study area were recorded as having VL with onset prior to 2002, 141 of these in 2000 or 2001. Since the data on VL occurrence, and onset and treatment times before 2002 is less complete and probably less reliable than from 2002 onwards, there may be missing information on potential sources of infection of cases with onset during 2002. We therefore exclude these cases from the infection probability part of the likelihood (L_2 in Eq. (S16)), but still impute their infection times in the MCMC algorithm (§2B.4) by drawing new infection times for them using the incubation period distribution, and accepting/rejecting these based on the effect they have on the likelihood of the infection times of other individuals.

2. Parameter Estimation

A. Bayesian inference. Since the infection times of VL cases ($\mathbf{E}$) and infection and recovery times of asymptomatic individuals ($\mathbf{A}$ and $\mathbf{R}_A$) were unobserved, and some recovery, relapse and relapse treatment times of VL cases ($\mathbf{R}'_I$, $\mathbf{I}'_R$, $\mathbf{R}_R$) were not recorded, and it is not computationally feasible to sum over all possible configurations of this missing data to calculate the complete data likelihood (Eq. (S16)), we treat the missing times as extra parameters and use a data augmentation approach to sample from the joint posterior distribution of the model parameters $\boldsymbol{\theta} = (\beta, \alpha, \epsilon, \delta, p)$ and the missing data $\mathbf{X}$ given the observed data $\mathbf{Y}$

$$\mathbb{P}(\boldsymbol{\theta}, \mathbf{X}|\mathbf{Y}) = \frac{\mathbb{P}(\mathbf{Y}, \mathbf{X}|\boldsymbol{\theta})\mathbb{P}(\boldsymbol{\theta})}{\mathbb{P}(\mathbf{Y})} \propto L(\mathbf{Z}; \boldsymbol{\theta})\mathbb{P}(\boldsymbol{\theta}). \tag{S17}$$

We do this using a Metropolis-within-Gibbs MCMC data augmentation algorithm in which we iterate between sampling from the conditional posterior distribution of the parameters given the observed data and the current value of the missing data, $\mathbb{P}(\boldsymbol{\theta}|\mathbf{Y}, \mathbf{X})$, and the conditional posterior distribution of the missing data given the current parameter values and the observed data, $\mathbb{P}(\mathbf{X}|\boldsymbol{\theta}, \mathbf{Y})$ (1, 29).

B. MCMC data augmentation scheme.

B.1. Asymptomatic infection times. To account for the uncertainty in the parameter estimates due to the missing asymptomatic infection and recovery times we need to estimate which individuals were asymptomatically infected during the study and when they were infected, as part of the MCMC algorithm. To do this, we assign every non-symptomatic individual j in the population an asymptomatic infection time and recovery time pair, $(A_j, R_j) \in \{V_j, V_j+1, \ldots, W_j-1, T+1\} \times \{A_j + 1, A_j+2, \ldots, W_j-1, T+1\}$, where asymptomatic infection and recovery before the study is labelled as $(A_j, R_j) = (0, 0)$, asymptomatic infection before and recovery during/after the study is labelled as $(A_j, R_j) = (0, s)$, $(s \in \{1, \ldots, T+1\})$, and no asymptomatic infection before or during the study is labelled as $(A_j, R_j) = (T+1, T+1)$. Posterior distributions for the asymptomatic infection and recovery time pairs are then estimated in the MCMC algorithm by proposing moves for these pairs and accepting/rejecting them based on the Metropolis-Hastings acceptance probability. The posterior distribution for which non-symptomatic individuals were asymptomatically infected during the study can be estimated from the proportion of time in the MCMC chain each individual spends in states other than $(0, 0)$, $(0, s)$ and $(T+1, T+1)$.

Since every non-symptomatic individual has an infection and recovery time pair, with a finite set of possible values, the dimension of the model remains fixed and reversible jump MCMC (RJMCMC) is not required. We note, however, that the algorithm described below for updating the asymptomatic infection and recovery time pairs is equivalent to a classical RJMCMC algorithm in which only individuals that are asymptomatically infected before or during the study have asymptomatic infection and recovery times and the number of asymptomatic infections varies as asymptomatic infection times are added or removed in the steps of the algorithm (30–33). As in the RJMCMC algorithm, the likelihood can be higher in the initial iterations of the chain than the value it ultimately converges to in our approach since the number of asymptomatic infections in the likelihood changes as the number of pairs corresponding to asymptomatic infection before or during the study changes, despite the fixed dimension of the parameter space.

In order to sample effectively from the posterior distribution of the asymptomatic infection and recovery times, we need to use a proposal distribution that allows us to efficiently navigate the very large space of possible asymptomatic infection and recovery time pairs. We therefore use the running estimate of the total infection pressure on each individual, averaged over the history of the MCMC chain, to propose new asymptomatic infection times for non-symptomatic individuals. The proposal distribution for individual j at the kth iteration of the MCMC chain thus consists of the probabilities of them being asymptomatically infected at each time point according to the mean infection pressure on them at time t from the previous samples in the chain, $\overline{\lambda}_j^{(k)}(t) = \frac{1}{k}\sum_{m=0}^{k-1} \lambda_j^{(m)}(t)$:

$$q_j^{(k)}(A_j) = \begin{cases} (1 - p_{S_0}(a_j))/C_j^{(k)} & \text{for } A_j = V_j, \\ p_{S_0}(a_j) e^{-\sum_{s=V_j+1}^{A_j-1} \overline{\lambda}_j^{(k)}(s-1)} \left(1 - e^{-(1-p_I)\overline{\lambda}_j^{(k)}(A_j-1)}\right)/C_j^{(k)} & \text{for } V_j < A_j \leq W_j - 1, \\ p_{S_0}(a_j) e^{-\sum_{s=V_j+1}^{W_j-1} \overline{\lambda}_j^{(k)}(s-1)}/C_j^{(k)} & \text{for } A_j = T+1, \end{cases} \tag{S18}$$

where $C_j = \sum_{t=V_j}^{W_j-1} q_j(t) + q_j(T+1)$ is a normalising constant to account for the fact that we know that j was not pre-symptomatically infected during the study. Although the infection pressure with a newly proposed asymptomatic infection and recovery time pair (A'_j, R'_j) will be different from that with the current pair (A_j, R_j), and the probability of the reverse move $(A'_j, R'_j) \to (A_j, R_j)$ will therefore not be equal to the probability of the forward move $(A_j, R_j) \to (A'_j, R'_j)$, the difference in the infection pressure between consecutive iterations will be $\mathcal{O}(\frac{1}{k})$. Hence, as the number of iterations becomes large, the adaptation will diminish (34) and $\overline{\lambda}_j^k(t)$ will tend towards a constant, such that the proposal probabilities for the forward and backward moves may be treated as equal.

B.2. Prior distributions. We use relatively weak gamma distributions ($\text{Gamma}(k, \theta)$ with shape parameter k and scale parameter θ, i.e. probability density function $f(x) = \frac{1}{\Gamma(k)\theta^k} x^{k-1} e^{-x/\theta}$), for the prior distributions for the transmission parameters β, α, ϵ and δ (which are non-negative), since there is little information available with which to construct informative priors (Table S3).

The mean of the prior distribution for α is chosen as 100m based on previous findings (1). A beta distribution, $\mathrm{Beta}(a, b)$, is chosen as a conjugate prior for the incubation period parameter p, since it is a probability ($p \in (0, 1]$). The parameters of the beta distribution are chosen to match the mean of the prior distribution for the incubation period ($\mathrm{NB}(r, p)$, $p \sim \mathrm{Beta}(a, b)$) with the estimated mean incubation period (∼5 months) taken from previous analysis of diagnostic data from a subset of the study population (26). With this choice of prior, the conditional posterior distribution of p is a beta distribution:

$$(p|\boldsymbol{\beta}, \mathbf{Y}, \mathbf{X}) \sim \mathrm{Beta}\left(a + rn_I, b + \sum_{j=1}^{n_I} IP_j - n_I\right), \quad \text{[S19]}$$

where $\boldsymbol{\beta} = (\beta, \alpha, \epsilon, \delta)$, so p can be updated efficiently in the MCMC by drawing from this full conditional distribution rather than using a random walk Metropolis-Hastings update.

Table S3. Definitions and prior distributions of estimated parameters.

Parameter (units)	Definition	Prior distribution
β (month^{-1})	Rate constant for spatial transmission	$\mathrm{Gamma}(1, 1)$
α (m)	Inverse distance decay rate of spatial transmission kernel	$\mathrm{Gamma}(1, 100)$
ϵ (month^{-1})	Background transmission rate	$\mathrm{Gamma}(1, 1)$
δ	Additional within-household transmission rate	$\mathrm{Gamma}(1, 1)$
p	Incubation period distribution parameter	$\mathrm{Beta}(22, 28)$

B.3. Initial parameter values and missing data values. Informed guesses based on previous results (1) and preliminary runs of the MCMC are used for the starting values for the model parameters to reduce the burn-in time, $\boldsymbol{\theta}_0 = (\beta_0, \alpha_0, \epsilon_0, \delta_0, p_0) = (3, 100, 0.001, 0.001, 3/7)$. The initialisation of the missing data, $\mathbf{X}$, is more involved as it requires picking starting values for the asymptomatic infection and recovery times of all non-symptomatic individuals in the population (which determine their initial statuses and whether they were infected or not by the end of the study), along with the infection times of all VL cases, and proceeds as follows:

- For each VL case j missing a treatment time, draw a treatment time using Eq. (S5), i.e.

$$R'_{j,0} = I_{j,0} + OT'_{j,0}, \quad OT'_{j,0} \sim \mathrm{NB}(r_1, p_1) \quad \text{[S20]}$$

- For each VL case j missing a treatment time who may have had active VL at the start of the study (assumed to be only those who had onset after January 2001 since those with onset before January 2001 were likely to have been treated by January 2002 (Fig. S2A)):
  - if their onset time $I_{j,0}$ is missing, draw it from $U(-11, 0)$ (i.e. uniformly from January 2001–December 2001)
  - draw their treatment time $R_{j,0}$ as in Eq. (S20)
- For each VL case j that suffered relapse:
  - if their relapse time $I_{Rj,0}$ is missing, draw it using Eq. (S8)
  - draw their relapse treatment time $R_{Rj,0}$ using Eq. (S5), i.e.

$$R_{Rj,0} = I_{Rj,0} + OT_{j,0}, \quad OT_{j,0} \sim \mathrm{NB}(r_1, p_1)$$

- For each VL case j, draw an infection time:

$$E_{j,0} = I_j - IP_{j,0}, \quad IP_{j,0} \sim \mathrm{NB}(r, p_0)$$

- For each non-symptomatic individual j:
  - draw their initial status (susceptible, currently asymptomatically infected, or previously asymptomatically infected and recovered) according to Eqs (S13)–(S15)
  - if they are initially recovered from previous asymptomatic infection, set $(A_{j,0}, R_{j,0}) = (0, 0)$
  - if they are initially actively asymptomatically infected, draw their recovery time $R_{j,0}$ from $\{1, \ldots, W_j - 1, T + 1\}$ with probability

$$\begin{cases} f_A(R_{j,0}) & \text{for } 1 \le R_{j,0} \le W_j - 1, \\ 1 - F_A(W_j - 1) & \text{for } R_{j,0} = T + 1 \end{cases}$$

  - for the subset that are initially susceptible:

- * calculate the infection pressure on them (Eq. (S9)) from VL cases and PKDL cases with prior VL
  * use this to calculate the probability of asymptomatic infection at each time point as in Eq. (S18), i.e. with $\overline{\lambda}_j(t)$ replaced by the infection pressure from VL and PKDL cases
  * draw $\frac{1-p_I}{p_I}n_I - n_{AP}$ individuals, where n_{AP} is the number of asymptomatic individuals who develop PKDL, to be asymptomatically infected according to their cumulative probability of asymptomatic infection before the end of the study, $\frac{\sum_{t=V_j}^{W_j-1} q_j(t)}{\sum_{i\in\mathcal{S}(0)}\sum_{t=V_j}^{W_j-1} q_i(t)}$
  * for each asymptomatic individual draw an infection time $A_{j,0} \in \{V_j+1,\ldots,W_j-1\}$ with probability

$$\frac{q_j(A_{j,0})}{\sum_{t=V_j+1}^{W_j-1} q_j(t)},$$

    and a recovery time $R_{j,0} \in \{A_j+1,\ldots,W_j-1,T+1\}$ with probability

$$\begin{cases} f_A(R_{j,0}-A_{j,0}) & \text{for } A_{j,0} < R_{j,0} \leq W_j - 1, \\ 1 - F_A(W_j-1-A_{j,0}) & \text{for } R_{j,0} = T+1. \end{cases}$$

  * for all individuals not infected before the end of the study set $(A_{j,0}, R_{j,0}) = (T+1, T+1)$

- For each PKDL case j without prior VL, set a dormant infection start time, $D_{j,0}$, by drawing a dormant infection duration and subtracting it from their PKDL onset time,

$$D_{j,0} = P_j - DIP_{j,0}, \quad DIP_{j,0} \sim \text{NB}(r_3, p_3),$$

  and then draw an asymptomatic infection time, $A_{j,0}$, conditional on $A_{j,0} > V_j$,

$$A_{j,0} = D_{j,0} - AIP_{j,0}, \quad AIP_{j,0} \sim \text{Geom}(p_2).$$

The MCMC algorithm was run from a range of initial parameter values and asymptomatic infection and recovery time configurations (with different numbers of individuals asymptomatically infected before the study and during the study etc.) to test convergence, and in all cases converged to the same posterior distributions.

B.4. MCMC algorithm. With the initial parameter values, missing data and priors chosen as described, the MCMC algorithm proceeds by repeating the steps below. Note that throughout the following we suppress notation of conditional dependencies in the likelihood terms where they are obvious to maintain legibility. The algorithm also accounts for the fact that some individuals were born or migrated or died during the study when updating the unknown pre-symptomatic infection times and asymptomatic infection and recovery times (using the birth/migration/death times as bounds on the proposed unobserved times), but we omit these details from the following description for simplicity.

We update 20% of infection times of VL cases and 20% of infection and recovery times of asymptomatic individuals in each iteration of the algorithm to strike a balance between efficient mixing of the MCMC chain and computational speed. The algorithm is run for $N = 10^5$ iterations, including a burn-in of $20,000$ iterations which is discarded.

Terms in the full likelihood (Eq. (S16)) for the asymptomatic infection duration, dormant infection duration, VL symptom duration of cases with missing onset and/or treatment time, and relapse duration do not appear in the acceptance probabilities for the updates for asymptomatic infection and recovery times, dormant infection times, missing onset and treatment times, and missing relapse and relapse treatment times (steps 4–10 below), as they cancel with the proposal probabilities for the newly proposed times, and are unaffected by the other steps in the algorithm.

1. **Update transmission parameters:**
   Update the transmission parameters $\boldsymbol{\beta} = (\beta, \alpha, \epsilon, \delta)$ conditional on the current incubation period distribution parameter p and missing data values $\mathbf{X}$ and observed data $\mathbf{Y}$, using an adaptive random walk Metropolis-Hastings step:
   (a) Propose new values $\boldsymbol{\beta}'$ as described in §2B.5.
   (b) Accept $\boldsymbol{\beta}'$ with probability

$$\min\left(1, \frac{L_1(\boldsymbol{\beta}')L_2(\boldsymbol{\beta}')L_3(\boldsymbol{\beta}')L_4(\boldsymbol{\beta}')}{L_1(\boldsymbol{\beta})L_2(\boldsymbol{\beta})L_3(\boldsymbol{\beta})L_4(\boldsymbol{\beta})}\frac{\mathbb{P}(\boldsymbol{\beta}')}{\mathbb{P}(\boldsymbol{\beta})}\right).$$

2. **Update incubation period distribution parameter:**
   Update p by drawing from its conditional posterior distribution $\mathbb{P}(p|\boldsymbol{\beta}, \mathbf{Y}, \mathbf{X})$ (Eq. (S19)).

3. **Move pre-symptomatic infection times:**
   Update one-at-a-time the pre-symptomatic infection times of a random 20% of VL cases for whom both the onset and treatment times were observed, and the infection times of all cases missing their onset and/or treatment time:

(a) For each case j, propose a new infection time conditional on the current values of the other infection times and the model parameters, $E'_j|((\mathbf{X} \setminus \{E_j\}), \mathbf{Y}, \boldsymbol{\theta})$, using an independence sampler, i.e. propose a new incubation period $IP'_j \sim \mathrm{NB}(r,p)$ and subtract this from the onset time:

$$E'_j = I_j - IP'_j.$$

(b) Accept the infection time move with probability

$$\min\left(1, \frac{L_1(E'_j|\mathbf{E}_{-j})L_2(E'_j|\mathbf{E}_{-j})L_3(E'_j|\mathbf{E}_{-j})L_4(E'_j|\mathbf{E}_{-j})}{L_1(E_j|\mathbf{E}_{-j})L_2(E_j|\mathbf{E}_{-j})L_3(E_j|\mathbf{E}_{-j})L_4(E_j|\mathbf{E}_{-j})}\frac{f_E(IP_j)}{f_E(IP'_j)}\right).$$

where $\mathbf{E}_{-j} = (E_1, \ldots, E_{j-1}, E_{j+1}, \ldots, E_{n_I})$ is the set of pre-symptomatic infection times with j's infection time E_j removed.

4. **Update asymptomatic infection and recovery times:**
Update one-at-a-time the asymptomatic infection and recovery times of 20% of non-symptomatic individuals as follows. This step may appear complicated, but essentially consists of proposing a new asymptomatic infection time and recovery time pair (A'_j, R'_j) from anywhere in an approximately triangular grid of possibilities in discrete (A,R)-space (since $R_j \geq A_j$ with equality only when $A_j = 0$ or $A_j = T+1$), recalculating the parts of the likelihood that change with (A'_j, R'_j) and accepting/rejecting according to the product of the likelihood ratio and the proposal ratio for the move $(A_j, R_j) \to (A'_j, R'_j)$.

(a) Choose a non-symptomatic individual j with probability proportional to their cumulative probability of being asymptomatically infected before the end of the study $\sum_{t=V_j}^{W_j-1} q_j^{(k)}(t)$.

(b) Draw a new asymptomatic infection time $A'_j \in \{V_j, \ldots, W_j-1, T+1\}$ from the asymptomatic infection time proposal distribution $q_j^{(k)}(\cdot)$ (Eq. (S18)).

(c) i. If $A_j = 0$:

A. If $A'_j = 0$, propose a new $R'_j \in \{0, 1, \ldots, W_j-1, T+1\}$ with probability:

$$\begin{cases} \frac{p_{R_0}(a_j)}{(p_{A_0}(a_j)+p_{R_0}(a_j))} & \text{for } R'_j = 0, \\ \frac{p_{A_0}(a_j)}{p_{A_0}(a_j)+p_{R_0}(a_j)} f_A(R'_j) & \text{for } 1 \leq R'_j \leq W_j - 1, \\ \frac{p_{A_0}(a_j)}{p_{A_0}(a_j)+p_{R_0}(a_j)} \big(1 - F_A(W_j-1)\big) & \text{for } R'_j = T+1. \end{cases}$$

Accept (A'_j, R'_j) with probability

$$\min\left(1, \frac{L_1(A'_j,R'_j|\mathbf{A}_{-j},\mathbf{R}_{-j})L_2(A'_j,R'_j|\mathbf{A}_{-j},\mathbf{R}_{-j})L_4(A'_j,R'_j|\mathbf{A}_{-j},\mathbf{R}_{-j})L_5(A'_j,R'_j|\mathbf{A}_{-j},\mathbf{R}_{-j})}{L_1(A_j,R_j|\mathbf{A}_{-j},\mathbf{R}_{-j})L_2(A_j,R_j|\mathbf{A}_{-j},\mathbf{R}_{-j})L_4(A_j,R_j|\mathbf{A}_{-j},\mathbf{R}_{-j})L_5(A_j,R_j|\mathbf{A}_{-j},\mathbf{R}_{-j})}Q\right), \quad \text{[S21]}$$

where

$$Q = \begin{cases} 1 & \text{if } R_j = 0, R'_j = 0, \\ \frac{p_{A_0}(a_j)}{p_{R_0}(a_j)} & \text{if } R_j > 0, R'_j = 0, \\ \frac{p_{R_0}(a_j)}{p_{A_0}(a_j)} & \text{if } R_j = 0, R'_j > 0, \\ 1 & \text{if } R_j > 0, R'_j > 0, \end{cases}$$

and $\mathbf{A}_{-j} = \mathbf{A} \setminus \{A_j\}$ and $\mathbf{R}_{-j} = \mathbf{R} \setminus \{R_j\}$.

B. If $A'_j = T+1$, set $R'_j = T+1$ and accept (A'_j, R'_j) with probability in Eq. (S21), but where Q is now

$$Q = \begin{cases} \frac{p_{R_0}(a_j)}{p_{A_0}(a_j)+p_{R_0}(a_j)} \frac{q_j^{(k)}(0)}{q_j^{(k)}(T+1)} & \text{if } R_j = 0, \\ \frac{p_{A_0}(a_j)}{p_{A_0}(a_j)+p_{R_0}(a_j)} \frac{q_j^{(k)}(0)}{q_j^{(k)}(T+1)} & \text{if } R_j > 0. \end{cases}$$

C. If $A'_j \in [1, T]$, propose $R'_j \in \{A'_j+1, A'_j+2, \ldots, W_j-1, T+1\}$ with probability:

$$\begin{cases} f_A(R'_j - A'_j) & \text{for } A'_j < R'_j \leq W_j - 1, \\ 1 - F_A(W_j - 1 - A'_j) & \text{for } R'_j = T+1, \end{cases}$$

and accept (A'_j, R'_j) with probability in Eq. (S21) but with

$$Q = \begin{cases} \frac{p_{R_0}(a_j)}{p_{A_0}(a_j)+p_{R_0}(a_j)} \frac{q_j^{(k)}(0)}{q_j^{(k)}(A'_j)} & \text{if } R_j = 0, \\ \frac{p_{A_0}(a_j)}{p_{A_0}(a_j)+p_{R_0}(a_j)} \frac{q_j^{(k)}(0)}{q_j^{(k)}(A'_j)} & \text{if } R_j > 0. \end{cases}$$

ii. If $A_j = T+1$:

A. If $A'_j = T+1$, then $R'_j = T+1$, so accept immediately as the likelihood does not change.

B. If $A'_j = 0$, follow Step 4(c)iA, except with

$$Q = \begin{cases} \frac{1}{p_{R_0}(a_j)/\left(p_{A_0}(a_j)+p_{R_0}(a_j)\right)} \frac{q_j^{(k)}(T+1)}{q_j^{(k)}(0)} & \text{if } R'_j = 0, \\ \frac{1}{p_{A_0}(a_j)/\left(p_{A_0}(a_j)+p_{R_0}(a_j)\right)} \frac{q_j^{(k)}(T+1)}{q_j^{(k)}(0)} & \text{if } R'_j > 0. \end{cases}$$

C. If $A'_j \in [1, T]$, follow Step 4(c)iC, except with

$$Q = \frac{q_j^{(k)}(T+1)}{q_j^{(k)}(A'_j)}.$$

iii. If $A_j \in [1, T]$:

A. If $A'_j = 0$, follow Step 4(c)iA, but with Q replaced by

$$Q = \begin{cases} \frac{1}{p_{R_0}(a_j)/\left(p_{A_0}(a_j)+p_{R_0}(a_j)\right)} \frac{q_j^{(k)}(A_j)}{q_j^{(k)}(0)} & \text{if } R'_j = 0, \\ \frac{1}{p_{A_0}(a_j)/\left(p_{A_0}(a_j)+p_{R_0}(a_j)\right)} \frac{q_j^{(k)}(A_j)}{q_j^{(k)}(0)} & \text{if } R'_j > 0. \end{cases}$$

B. If $A'_j = T+1$, follow Step 4(c)iB except with

$$Q = \frac{q_j^{(k)}(A_j)}{q_j^{(k)}(T+1)}.$$

C. If $A_j \in [1, T]$, follow Step 4(c)iC but with

$$Q = \frac{q_j^{(k)}(A_j)}{q_j^{(k)}(A'_j)}.$$

5. **Update asymptomatic infection times and dormant infection times of PKDL cases without prior VL:** Update the asymptomatic infection and dormant infection times for each PKDL case j without prior VL, conditional on the asymptomatic infection time being after the start of the study or j's birth (whichever is later):

   (a) Propose a new time, D'_j, for recovery to dormant infection from asymptomatic infection using an independence sampler:
   $$D'_j = P_i - DIP_j, \quad DIP_j \sim \text{NB}(r_3, p_3).$$

   (b) Propose a new asymptomatic infection time, A'_j:
   $$A'_j = D'_j - AIP_j, \quad AIP_j \sim \text{Geom}(p_2).$$

   (c) If $A'_j < V_j + 1$, reject immediately. Otherwise accept (A'_j, D'_j) with probability
   $$\min\left(1, \frac{L_1(A'_j, D'_j|\mathbf{A}_{-j}, \mathbf{D}_{-j})L_2(A'_j, D'_j|\mathbf{A}_{-j}, \mathbf{D}_{-j})L_4(A'_j, D'_j|\mathbf{A}_{-j}, \mathbf{D}_{-j})}{L_1(A_j, D_j|\mathbf{A}_{-j}, \mathbf{D}_{-j})L_2(A_j, D_j|\mathbf{A}_{-j}, \mathbf{D}_{-j})L_4(A_j, D_j|\mathbf{A}_{-j}, \mathbf{D}_{-j})}\right),$$
   where $\mathbf{D}_{-j} = \mathbf{D} \setminus \{D_j\}$.

6. **Update missing treatment times of VL cases during the study:** Update the treatment time of each VL case j with a missing treatment time but known onset time, conditional on the treatment time being before their PKDL onset:

   (a) Propose a new treatment time:
   $$D'_j = I_j + OT'_j, \quad OT'_j \sim \text{NB}(r_1, p_1).$$

   (b) If $D'_j > P_j - 1$, reject immediately. Otherwise accept D'_j with probability
   $$\min\left(1, \frac{L_1(D'_j|\mathbf{D}_{-j})L_2(D'_j|\mathbf{D}_{-j})L_4(D'_j|\mathbf{D}_{-j})}{L_1(D_j|\mathbf{D}_{-j})L_2(D_j|\mathbf{D}_{-j})L_4(D_j|\mathbf{D}_{-j})}\right).$$

7. **Update whole onset-to-treatment period of potentially initially active VL cases:**
Update the onset and treatment times of all cases who potentially had active VL at the start of the study ($t = 0$) who are missing both onset and treatment times, one case at a time. For each case j:

   (a) Propose new onset and treatment times, I'_j and R'_j:

$$I'_j = I_j + M, \quad M \sim \text{round}(\text{Normal}(0,4)) \setminus \{0\}$$
$$R'_j = I'_j + OT'_j, \quad OT'_j \sim \text{NB}(r_1, p_1).$$

   (b) If I'_j is not in j's onset year or $R'_j > \min(P_j - 1, M_j, T)$, reject immediately. Otherwise, accept the new times with probability

$$\min\left(1, \frac{L_1(I'_j, R'_j|\mathbf{I}_{-j}, \mathbf{R}_{-j})L_2(I'_j, R'_j|\mathbf{I}_{-j}, \mathbf{R}_{-j})L_4(I'_j, R'_j|\mathbf{I}_{-j}, \mathbf{R}_{-j})}{L_1(I_j, R_j|\mathbf{I}_{-j}, \mathbf{R}_{-j})L_2(I_j, R_j|\mathbf{I}_{-j}, \mathbf{R}_{-j})L_4(I_j, R_j|\mathbf{I}_{-j}, \mathbf{R}_{-j})}\right),$$

   where $\mathbf{I}_{-j} = \mathbf{I} \setminus \{I_j\}$.

8. **Update missing treatment times of potentially initially active VL cases:**
Update the treatment times of cases who potentially had active VL at the start of the study whose treatment times were not recorded but whose onset times are known, one by one. For each case j:

   (a) Propose a new treatment time, R'_j:

$$R'_j = I_j + OT'_j, \quad OT'_j \sim \text{NB}(r_1, p_1).$$

   (b) If $R'_j > \min(P_j - 1, M_j, T)$, reject immediately. Otherwise, accept R'_j with probability

$$\min\left(1, \frac{L_1(R'_j|\mathbf{R}_{-j})L_2(R'_j|\mathbf{R}_{-j})L_4(R'_j|\mathbf{R}_{-j})}{L_1(R_j|\mathbf{R}_{-j})L_2(R_j|\mathbf{R}_{-j})L_4(R_j|\mathbf{R}_{-j})}\right).$$

9. **Update whole relapse period of cases missing both relapse and relapse treatment times:**
Update the relapse and relapse treatment times of all VL cases who suffered relapse during the study who are missing both times, one case at a time. For each case j:

   (a) Propose new relapse and relapse treatment times, I'_{Rj} and R'_{Rj}

$$I'_{Rj} = R_j + TR_j, \quad TR_j \sim \text{Geom}(p_4),$$
$$R'_{Rj} = I'_{Rj} + OT'_j, \quad OT'_j \sim \text{NB}(r_1, p_1).$$

   (b) If $I'_{Rj} > \min(P_j - 2, W_j - 2)$ or $R'_{Rj} > \min(P_j - 1, W_j - 1)$, reject immediately. Otherwise, accept I'_{Rj} and R'_{Rj} with probability

$$\min\left(1, \frac{L_1(I'_{Rj}, R'_{Rj}|\mathbf{I}_{R,-j}, \mathbf{R}_{R,-j})L_2(I'_{Rj}, R'_{Rj}|\mathbf{I}_{R,-j}, \mathbf{R}_{R,-j})L_4(I'_{Rj}, R'_{Rj}|\mathbf{I}_{R,-j}, \mathbf{R}_{R,-j})}{L_1(I_{Rj}, R_{Rj}|\mathbf{I}_{R,-j}, \mathbf{R}_{R,-j})L_2(I_{Rj}, R_{Rj}|\mathbf{I}_{R,-j}, \mathbf{R}_{R,-j})L_4(I_{Rj}, R_{Rj}|\mathbf{I}_{R,-j}, \mathbf{R}_{R,-j})}\right),$$

   where $\mathbf{I}_{R,-j} = \mathbf{I}_R \setminus \{I_{Rj}\}$ and $\mathbf{R}_{R,-j} = \mathbf{R}_R \setminus \{R_{Rj}\}$.

10. **Update missing relapse treatment times of relapse VL cases:**
Update the missing relapse treatment times of VL cases with known relapse times:

   (a) Propose a new relapse treatment time, R'_{Rj}

$$R'_{Rj} = I_{Rj} + OT'_j, \quad OT'_j \sim \text{NB}(r_1, p_1).$$

   (b) If $R'_{Rj} > \min(P_j - 1, W_j - 1)$, reject immediately. Otherwise, accept R'_{Rj} with probability

$$\min\left(1, \frac{L_1(R'_{Rj}|\mathbf{R}_{R,-j})L_2(R'_{Rj}|\mathbf{R}_{R,-j})L_4(R'_{Rj}|\mathbf{R}_{R,-j})}{L_1(R_{Rj}|\mathbf{R}_{R,-j})L_2(R_j|\mathbf{R}_{R,-j})L_4(R_{Rj}|\mathbf{R}_{R,-j})}\right).$$

B.5. Accelerated adaptive random walk Metropolis algorithm. We block update the transmission parameters in step 1 of the algorithm above using a combination of an 'Accelerated Shaping' algorithm and an 'Accelerated Scaling' algorithm. The Accelerated Shaping algorithm is a generalised version of the adaptive random walk Metropolis algorithm of Haario et al. (35), which uses the running estimate of the covariance matrix of the posterior distribution of the parameters from the MCMC chain in the multivariate normal proposal distribution to automatically tune the proposal covariance matrix to make efficient proposals. The Haario algorithm uses a fixed initial guess for the covariance matrix for a certain number of iterations at the start of the chain and then the estimate of the covariance matrix from the whole history of the chain after that, including the initial values and the burn-in, which can lead to slow convergence of the chain due to the sensitivity of the covariance estimate to outliers. The Accelerated Shaping algorithm instead begins adapting straight away and removes early iterations of the chain from the running estimate of the covariance matrix at a rate slower than it includes new iterations. This helps to ensure that the chain converges to the posterior mode quickly even if the initial guesses for the parameter values and covariance matrix are relatively poor.

If $\boldsymbol{\beta}_k$ is the vector of the values of the transmission parameters at the kth iteration, then the proposal for $\boldsymbol{\beta}$ at the $(k+1)$th iteration is given by

$$\boldsymbol{\beta}'_{k+1} = N(\boldsymbol{\beta}_k, 2.38^2 c_k^2 \boldsymbol{\Sigma}_{k+1}/n_{\boldsymbol{\beta}}) \quad \text{[S22]}$$

where $\boldsymbol{\Sigma}_{k+1}$ and $n_{\boldsymbol{\beta}}$ are the running estimate of the covariance matrix and dimension of the posterior density for $\boldsymbol{\beta}$, and c_k is a scaling constant that is tuned to achieve efficient mixing via the Accelerated Scaling algorithm (see below). To discard early iterations at a slower rate than new ones are included, a non-decreasing sequence of integers is used, $f(k) = \lfloor \frac{k}{2} \rfloor$, such that $f(0) = 0$ and $f(k+1) = f(k)$ or $f(k+1) = f(k) + 1$ for all k, and $\boldsymbol{\Sigma}_{k+1}$ for $k > 0$ is defined as

$$\boldsymbol{\Sigma}_{k+1} = \left(\frac{k - f(k)}{k - f(k) + k_0}\right) \mathrm{cov}(\boldsymbol{\beta}_{f(k)}, \ldots, \boldsymbol{\beta}_k) + \left(\frac{k_0}{k - f(k) + k_0}\right) \boldsymbol{\Sigma}_0, \quad \text{[S23]}$$

where

$$\mathrm{cov}(\boldsymbol{\beta}_{f(k)}, \ldots, \boldsymbol{\beta}_k) = \frac{1}{k - f(k)} \sum_{i=f(k)}^{k} \left(\boldsymbol{\beta}_i \boldsymbol{\beta}_i^T - \overline{\boldsymbol{\beta}}_{i+1} \overline{\boldsymbol{\beta}}_{i+1}^T\right)$$

is the empirical covariance of the last $k - f(k) + 1$ samples of $\boldsymbol{\beta}$ from the chain,

$$\overline{\boldsymbol{\beta}}_{k+1} = \frac{1}{k - f(k) + 1} \sum_{i=f(k)}^{k} \overline{\boldsymbol{\beta}}_i$$

is the mean of the last $k - f(k) + 1$ samples, $\boldsymbol{\Sigma}_0$ is the initial guess for the covariance matrix, and k_0 determines the rate at which the influence of $\boldsymbol{\Sigma}_0$ on $\boldsymbol{\Sigma}_{k+1}$ decreases (the weight of $\boldsymbol{\Sigma}_0$ halves after the first $2k_0$ iterations). We use $k_0 = 1000$ here.

If $f(k) = f(k-1)$ (i.e. if k is odd with $f(k)$ chosen as above), an additional observation is added to the estimate of the covariance matrix:

$$\boldsymbol{\Sigma}_{k+1} = \frac{1}{k - f(k) + k_0} \left((k - 1 - f(k) + k_0)\boldsymbol{\Sigma}_k + \boldsymbol{\beta}_k \boldsymbol{\beta}_k^T + (k - f(k))\overline{\boldsymbol{\beta}}_k \overline{\boldsymbol{\beta}}_k^T - (k - f(k) + 1)\overline{\boldsymbol{\beta}}_{k+1} \overline{\boldsymbol{\beta}}_{k+1}^T\right),$$

where

$$\overline{\boldsymbol{\beta}}_{k+1} = \frac{k - f(k)}{k - f(k) + 1} \overline{\boldsymbol{\beta}}_k + \frac{1}{k - f(k) + 1} \boldsymbol{\beta}_k,$$

and if $f(k) = f(k-1) + 1$ (i.e. if k is even), the new observation replaces the oldest:

$$\boldsymbol{\Sigma}_{k+1} = \boldsymbol{\Sigma}_k + \frac{1}{k - f(k) + k_0} \left(\boldsymbol{\beta}_k \boldsymbol{\beta}_k^T - \boldsymbol{\beta}_{f(k)-1} \boldsymbol{\beta}_{f(k)-1}^T + (k - f(k) + 1) \left(\overline{\boldsymbol{\beta}}_k \overline{\boldsymbol{\beta}}_k^T - \overline{\boldsymbol{\beta}}_{k+1} \overline{\boldsymbol{\beta}}_{k+1}^T\right)\right)$$

where

$$\overline{\boldsymbol{\beta}}_{k+1} = \overline{\boldsymbol{\beta}}_k + \frac{1}{k - f(k) + 1} (\boldsymbol{\beta}_k - \boldsymbol{\beta}_{f(k)-1}).$$

It has been shown that $N(\boldsymbol{\beta}_k, 2.38^2 \boldsymbol{\Sigma}/n_{\boldsymbol{\beta}})$, where $\boldsymbol{\Sigma}$ is the covariance matrix of the posterior distribution, is the optimal proposal distribution for rapid convergence and efficient mixing of the MCMC chain for symmetric product-form posterior distributions as $n_{\boldsymbol{\beta}} \to \infty$, and leads to an acceptance rate of 23.4% (36, 37). This corresponds to a scaling of $c_k = 1$ in Eq. (S23). However, we are in a context with a large amount of missing data, which is strongly correlated with some of the transmission parameters (see §8B), so the posterior distribution is not symmetric, and this scaling is not optimal. We therefore scale c_k adaptively as the algorithm progresses to target an acceptance rate of approximately 23.4% for updates to $\boldsymbol{\beta}$ (Accelerated Scaling). We do this by rescaling c_k by a factor of $x_k > 1$ every time an acceptance occurs and by a factor of $x_k^{\nu/(\nu-1)} < 1$ every time a rejection occurs such that the acceptance rate ν approaches 23.4% in the long run,

$$c_{k+1} = \begin{cases} c_k x_k & \text{if proposal is accepted} \\ c_k x_k^{\nu/(\nu-1)} & \text{if proposal is rejected.} \end{cases}$$

In order to satisfy the 'Diminishing Adaptation' condition (38), which is necessary to ensure the Markov chain is ergodic and converges to the correct posterior distribution, it is required that c_k tends to a constant as $k \to \infty$. So that the adaptation diminishes as k increases, we use the sequence

$$x_k = 1 + \frac{m_0}{m_0 + k},$$

where m_0 is the number of iterations over which the scaling factor x_k decreases from 2 to 1.5. Here we use $m_0 = 100$.

3. Model Comparison

We compare the goodness of fit of models with and without additional within-household transmission using DIC (39). DIC measures the trade-off between model fit and complexity, and lower values indicate better fit. Since some variables were not observed, we use a version of DIC appropriate for missing data from (40), which is based on the complete data likelihood $L(\boldsymbol{\theta}; \mathbf{Z}) = \mathbb{P}(\mathbf{Y}, \mathbf{X}|\boldsymbol{\theta})$. This is equivalent to the standard version of DIC for fully observed data except that it is averaged over the missing data:

$$\begin{aligned}\text{DIC} &= D(\tilde{\boldsymbol{\theta}}) + 2p_D \\ &= \overline{D(\boldsymbol{\theta})} + p_D \\ &= 2\overline{D(\boldsymbol{\theta})} - D(\tilde{\boldsymbol{\theta}}) \\ &= -4\mathbb{E}_{\boldsymbol{\theta},\mathbf{X}}[\log L(\boldsymbol{\theta}; \mathbf{Y}, \mathbf{X})] + 2\mathbb{E}_{\mathbf{X}}[\log L(\tilde{\boldsymbol{\theta}}; \mathbf{Y}, \mathbf{X})], \end{aligned} \quad \text{[S24]}$$

where $D(\boldsymbol{\theta})$ is the deviance (the measure of model fit), given (up to an additive constant dependent only on the data) by

$$D(\boldsymbol{\theta}) = -2\log(L(\boldsymbol{\theta}; \mathbf{Z}));$$

p_D is the effective number of parameters in the model (the measure of model complexity), defined by

$$p_D = \overline{D(\boldsymbol{\theta})} - D(\tilde{\boldsymbol{\theta}}),$$

and $\tilde{\boldsymbol{\theta}}$ is an appropriate summary statistic for $\boldsymbol{\theta}$ from its posterior distribution (here we use the posterior mode).

The first term in Eq. (S24) can be straightforwardly estimated as the mean of the values of the log-likelihood over the MCMC samples:

$$\mathbb{E}_{\boldsymbol{\theta},\mathbf{X}}[\log L(\boldsymbol{\theta}; \mathbf{Y}, \mathbf{X})] \approx \frac{1}{N}\sum_{i=1}^{N} \log L(\boldsymbol{\theta}_i; \mathbf{Y}, \mathbf{X}_i).$$

The second term in Eq. (S24) is calculated by storing the values of the missing data while running the MCMC, re-running the full log-likelihood computation for each iteration using the stored values and the posterior mode, and averaging these values

$$\mathbb{E}_{\mathbf{X}}[\log L(\tilde{\boldsymbol{\theta}}; \mathbf{Y}, \mathbf{X})] \approx \frac{1}{N}\sum_{i=1}^{N} \log L(\tilde{\boldsymbol{\theta}}; \mathbf{Y}, \mathbf{X}_i).$$

Since there can be issues with using DIC for model comparison (41), we also compared the posterior distributions of the deviances of the models to assess differences in quality of fit and validated the DIC metric on simulated data (see §10).

4. Calculating the Contribution of Different Infection States to Transmission

We calculate the contribution of each infection state to the total infection pressure on all susceptible individuals $\lambda(t)$ (Fig. 2A in the main text), which determines the rate of new infections at time t, by summing the infection pressures on susceptible individuals from each state:

$$\lambda_{\mathcal{X}}(t) = \sum_{i \in \mathcal{S}(t)} \sum_{j \in \mathcal{X}(t)} \lambda_{ij}(t), \quad \text{[S25]}$$

where $\mathcal{X} \in \{\mathcal{A}, \mathcal{E}, \mathcal{I}, \mathcal{P}\}$ denotes the infection state. The relative contribution to the infection pressure on susceptible individuals (Fig. S21) is then given by

$$\frac{\lambda_{\mathcal{X}}(t)}{\sum_{\mathcal{Y} \in \{\mathcal{A},\mathcal{E},\mathcal{I},\mathcal{P}\}} \lambda_{\mathcal{Y}}(t) + \mathcal{S}(t)\epsilon} = \frac{\lambda_{\mathcal{X}}(t)}{\lambda(t)}. \quad \text{[S26]}$$

The relative contribution of state $\mathcal{X}$ to the infection pressure on the ith VL case at their infection time, i.e. the probability that i's infection source is $\mathcal{X}$ (Fig. 2B in the main text), is:

$$\frac{\sum_{j \in \mathcal{X}(E_i-1)} \lambda_{ij}(E_i - 1)}{\sum_{\mathcal{Y} \in \{\mathcal{A},\mathcal{E},\mathcal{I},\mathcal{P}\}} \left(\sum_{j \in \mathcal{Y}(E_i-1)} \lambda_{ij}(E_i - 1)\right) + \epsilon} = \frac{\sum_{j \in \mathcal{X}(E_i-1)} \lambda_{ij}(E_i - 1)}{\lambda_i(E_i - 1)}, \quad \mathcal{X} \in \{\mathcal{A}, \mathcal{E}, \mathcal{I}, \mathcal{P}\}. \quad \text{[S27]}$$

The probability that the ith VL case is infected from the background transmission is $\frac{\epsilon}{\lambda_i(E_i - 1)}$.

5. Reconstructing the Epidemic

A. Reconstructing the transmission tree. We reconstruct the transmission tree following the 'sequential approach' described in (42). We draw N samples $(\boldsymbol{\theta}_k, \mathbf{X}_k)$ $(k = 1, \ldots, N)$ from the joint posterior distribution from the MCMC, calculate the probability that infectee i was infected by individual j conditional on their infection time E_i

$$p_{ij|E_i} = \frac{\lambda_{ij}(E_i - 1)}{\sum_{l \in Inf(E_i-1)} \lambda_{il}(E_i - 1) + \epsilon} \quad \text{[S28]}$$

for all infectees and possible infectors for each sample, and draw an infector for each infectee from the posterior distribution of possible infectors $\{p_{ij|E_i}(\boldsymbol{\theta}_k, \mathbf{X}_k)\}$ from each sample. This yields a sample of N transmission trees drawn from their predictive distribution, and thus accounts for uncertainty in the infection source (given fixed values of the parameters and missing data), and uncertainty in the parameter values and missing data (over the posterior distribution). We use $N = 1000$ here.

We reconstruct the transmission chains for each infected individual by searching backwards through each sampled transmission tree for their infector, and the infector of their infector, and so on, until the "index" case (in practice the earliest observed case during the study period) in the chain is reached. This gives the transmission chain for each infectee in each transmission tree. We then find the set of all "index" cases (the roots of all the transmission chains) and calculate the mean time from their onset to the infections of their infectees, and mean distance to their infectees, in each infection generation, and average these quantities over all sampled transmission trees.

B. Calculating transmission distances and times. The mean infector-to-VL-infectee distance and mean infector-onset-to-VL-infectee-infection time for each VL and PKDL infector (Figures 4A and 4B in the main text) are calculated from the sample of N transmission trees by averaging the distances and times from each infector to their VL infectees within each tree, and then averaging these quantities over all the trees in which that VL/PKDL case is an infector:

$$\overline{d_j} = \frac{1}{|\mathcal{F}_j|} \sum_{k \in \mathcal{F}_j} \left(\frac{1}{|\mathcal{G}_{j,k}|} \sum_{i \in \mathcal{G}_{j,k}} d_{ij} \right) \quad \text{[S29]}$$

$$\overline{\tau_j} = \frac{1}{|\mathcal{F}_j|} \sum_{k \in \mathcal{F}_j} \left(\frac{1}{|\mathcal{G}_{j,k}|} \sum_{i \in \mathcal{G}_{j,k}} \tau_{ij}^{(k)} \right) \quad \text{[S30]}$$

where $\tau_{ij}^{(k)}$ is the time between j's onset and i's infection in the kth tree, $\mathcal{F}_j$ is the set of all trees in which j is an infector, $\mathcal{G}_{j,k}$ is the set of all infectees of j in the kth tree, and $|\cdot|$ denotes set size.

C. Calculating reproduction numbers. The individual-level reproduction number for infectious individual j is obtained by summing the probabilities in Eq. (S28) over all individuals i that j could have infected (43, 44):

$$\mathcal{R}_j = \sum_{i=1}^{n} p_{ij|E_i}. \quad \text{[S31]}$$

To account for uncertainty in infected individuals' unobserved infection times these reproduction numbers are averaged over the joint posterior distribution of the transmission parameters and missing data:

$$\overline{\mathcal{R}_j} = \sum_{\mathbf{X} \in \boldsymbol{\Omega}} \int \mathcal{R}_j(\boldsymbol{\theta}, \mathbf{X}) \mathbb{P}(\boldsymbol{\theta}, \mathbf{X}) \, \mathrm{d}\boldsymbol{\theta} \approx \frac{1}{N} \sum_{k=1}^{N} \mathcal{R}_j^{(k)}, \quad \text{[S32]}$$

where $\boldsymbol{\Omega}$ is the space of all possible values of the missing data $\mathbf{X}$, $\mathcal{R}_j^{(k)}$ is individual j's reproduction number from the kth iteration of the MCMC chain (with the burn-in removed), and N is a suitably large number of samples from the posterior distribution (we use $N = 1000$). Since we are interested in comparing the numbers of new infections generated by VL and PKDL cases, in the main text we separate the expected numbers of secondary infections coming from the VL episodes and PKDL episodes of individuals who had both VL and PKDL (Fig. 5A in the main text) (i.e. we effectively treat them as coming from separate individuals), and hence no longer refer to the numbers of secondary infections per VL case and per PKDL case as reproduction numbers.

The time-dependent overall effective reproduction number (Fig. 5C in the main text) can also be calculated from the transmission probabilities as the mean of the individual reproduction numbers for all VL cases and asymptomatic individuals with onset and infection, respectively, at time t (43):

$$R_e(t) = \frac{\sum_{j \in (\mathcal{A}(t) \cup \mathcal{I}(t)) \setminus (\mathcal{A}(t-1) \cup \mathcal{I}(t-1))} \mathcal{R}_j}{\left| \big(\mathcal{A}(t) \cup \mathcal{I}(t)\big) \setminus \big(\mathcal{A}(t-1) \cup \mathcal{I}(t-1)\big) \right|}. \quad \text{[S33]}$$

The absolute contribution of each infectious state to the effective reproduction number at time t is:

$$\frac{\sum_{j \in \mathcal{X}(t) \setminus \mathcal{X}(t-1)} \mathcal{R}_j}{\left| \left(\mathcal{A}(t) \cup \mathcal{I}(t) \right) \setminus \left(\mathcal{A}(t-1) \cup \mathcal{I}(t-1) \right) \right|} \tag{S34}$$

where $\mathcal{X} \in \{\mathcal{A}, \mathcal{I}\}$ denotes the infectious state, and, as described above, in the main text we split the numbers of secondary infections ($\mathcal{R}_j$) arising from VL and PKDL for cases that had both.

6. Model Simulations

To assess the fit of the model and simulate hypothetical interventions against PKDL, we create a stochastic simulation version of the individual-level spatiotemporal transmission model described above. We follow standard stochastic simulation methodology for discrete-time individual-level transmission models (45), converting infection event rates into probabilities in order to simulate who gets infected in each month. We assume that an individual's progression through different infection states following infection occurs independently of the rest of the epidemic (i.e. is either governed by internal biological processes or random external processes of detection), which enables the simulation of an individual's full infection history from the point of infection.

So that we can simulate durations of PKDL infectiousness, we fit a negative binomial distribution $\mathrm{NB}(r_5, p_5)$ to the observed PKDL onset-to-treatment times and onset-to-resolution times for self-resolving PKDL cases in the data:

$$f_P(x) = \mathbb{P}(R_{Pj} - D_j = x) = \frac{\Gamma(r_5 + x - 1)}{(x-1)!\Gamma(r_5)} p_5^{r_5} (1 - p_5)^{x-1}, \quad x \in \{1, 2, 3, \dots\}, \tag{S35}$$

by maximum likelihood estimation, which yields $r_5 = 1.18$, $p_5 = 0.066$ (corresponding to a mean duration of PKDL infectiousness of 18 months).

We simulate epidemics for the study population starting from January 2003 (at which point all but one of the paras had had at least 1 VL case since January 2002) using the individual-level location and demographic information (months of birth, death and migration) from the observed data, and posterior samples of the parameter values and individuals' infection statuses in December 2002 obtained from the MCMC algorithm.

Given these pieces of information, the simulation algorithm proceeds as follows:

1. Starting at $t = 12$, draw the times of the next events for all individuals that have already been infected, e.g. VL onset times for individuals already pre-symptomatically infected, using the size-biased negative binomial distributions corresponding to Eq. (S4), Eq. (S5), Eq. (S7), and Eq. (S35), and Eq. (S6) for the asymptomatic infection durations. The size-biased negative binomial distribution accounts for the fact that individuals with left-censored infection times who are observed to be actively infected at a particular point in time are likely to have had long durations of infection if the infection duration is negative binomially distributed (46). The PMF of a size-biased negative binomial random variable X^* corresponding to $X \sim \mathrm{NB}$ is:

$$\mathbb{P}(X^* = x^*) = \frac{\mathbb{P}(X \geq x^*)}{\mathbb{E}[X]}$$

2. Simulate the times of the subsequent events for these individuals by drawing from Eq. (S4)–Eq. (S7) and Eq. (S35). Assign each PKDL case an infectiousness by drawing from $\mathrm{Cat}(\{h_1, h_2, h_3, h_u\}, \mathbf{p})$, where $\mathrm{Cat}(\cdot)$ is the categorical distribution and $\mathbf{p} = (101/190, 31/190, 6/190, 52/190)$ are the probabilities of the different lesion types according to their observed frequencies in the data (Table S1).

3. For each susceptible individual i from the set of individuals who have been born/immigrated and have not yet died/emigrated, determine whether they become infected:

   (a) Infection occurs with probability $1 - e^{-\lambda_i(t)}$, where $\lambda_i(t)$ is given by Eq. (S9).

   (b) Else they remain susceptible.

4. Set $t = t + 1$.

5. For each infection, decide if it is a pre-symptomatic infection or an asymptomatic infection:

   (a) Pre-symptomatic infection occurs with probability p_I. In which case, draw an onset time:

   $$I_i = t + IP_i, \quad IP_i \sim \mathrm{NB}(r, p),$$

   and decide whether the individual subsequently develops PKDL or not:

   i. PKDL occurs with probability p_P. In which case draw times for progression to dormant infection, PKDL onset and recovery:

   $$\begin{aligned} D_i &= I_i + OT_i, \quad OT_i \sim \mathrm{NB}(r_1, p_1), \\ P_i &= D_i + DIP_i, \quad DIP_i \sim \mathrm{NB}(r_3, p_3), \\ R_i &= P_i + PR_i, \quad PR_i \sim \mathrm{NB}(r_5, p_5), \end{aligned}$$

   and assign a PKDL infectiousness to the individual by drawing from $\mathrm{Cat}(\{h_1, h_2, h_3, h_u\}, \mathbf{p})$.

ii. Else the individual recovers upon treatment, so draw a treatment time:

$$R_i = I_i + OT_i, \quad OT_i \sim \text{NB}(r_1, p_1).$$

(b) Else it is an asymptomatic infection. In which case, decide whether the individual progresses to PKDL or not:

i. Progression to PKDL occurs with probability p_D. In which case, draw times for progression to dormant infection, PKDL onset and recovery:

$$\begin{aligned} D_i &= I_i + AIP_i, \quad AIP_i \sim \text{Geom}(p_2), \\ P_i &= D_i + DIP_i, \quad DIP_i \sim \text{NB}(r_3, p_3), \\ R_i &= P_i + PR_i, \quad PR_i \sim \text{NB}(r_5, p_5), \end{aligned}$$

and assign a PKDL infectiousness by drawing from $\text{Cat}(\{h_1, h_2, h_3, h_u\}, \mathbf{p})$.

ii. Else the individual recovers without developing PKDL, so draw a recovery time:

$$R_i = A_i + AIP_i, \quad AIP_i \sim \text{Geom}(p_2).$$

6. Repeat steps 3–5 until $t = T$ (as the last iteration).

We run 10,000 simulations of the model for each PKDL intervention scenario (normal interventions, complete PKDL prevention ($h_1 = h_2 = h_3 = h_u = 0$) and halving the mean duration of infectiousness ($r_5 = 1.18$, $p_5 = 0.13$)), consisting of 100 simulations (to account for stochastic uncertainty) of each of 100 posterior samples for the model parameters and individuals' infection statuses in December 2002 (to account for uncertainty in their values given the observed data).

7. Code

Code for running the MCMC algorithm and processing the MCMC output was written in MATLAB R2017b (47) and the simulation code was written in Julia 1.0.5 (48, 49). All code is freely available from https://github.com/LloydChapman/VLSpatiotemporalModelling.

8. MCMC Output

A. Model selection. The posterior deviance distributions and DIC values for the different models tested are presented in Fig. S5 and Table S4 respectively. Based on the DIC values, models with additional within-household transmission fit better than those without. We focus on the output of the model with the highest level of relative asymptomatic and pre-symptomatic infectiousness (both 2% as infectious as VL) below and in the main text, as there is some evidence to suggest that asymptomatic individuals occasionally generate VL cases (50, 51) and this is the most conservative assumption in terms of estimating the contribution of PKDL to transmission.

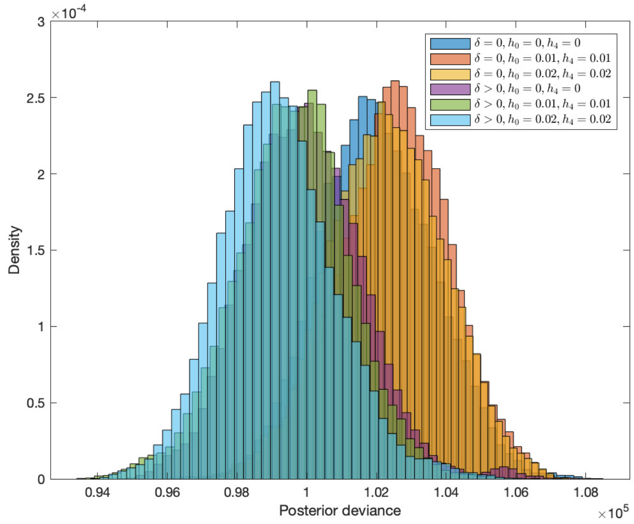

Fig. S5. Posterior deviance distributions for different models tested

B. Parameter estimates. The modes and 95% highest posterior density intervals (HPDIs) of the transmission parameters (β, α, ϵ and δ) and incubation period distribution parameter p for the different models are shown in Table S4. The parameter estimates are very similar across the different models and vary in the way expected – the spatial transmission rate constant β and background transmission rate ϵ are lower for models with additional within-household transmission ($\delta > 0$) and decrease with increasing relative asymptomatic infectiousness h_4, and the mode for α is slightly larger for models with $\delta > 0$ (since a flatter kernel shape compensates for the extra within-household transmission). The posterior distributions for the incubation period distribution parameter p correspond to a mean incubation period of 5.4–6.7 months (95% HPDIs (4.6,6.2)–(5.7,7.8) months).

The log-likelihood trace and posterior distributions for the parameters for the model with additional within-household transmission and 2% relative asymptomatic and pre-symptomatic infectiousness are shown in Fig. S6. The parameters are clearly well defined by the data, as the posterior distributions differ significantly from the weak prior distributions.

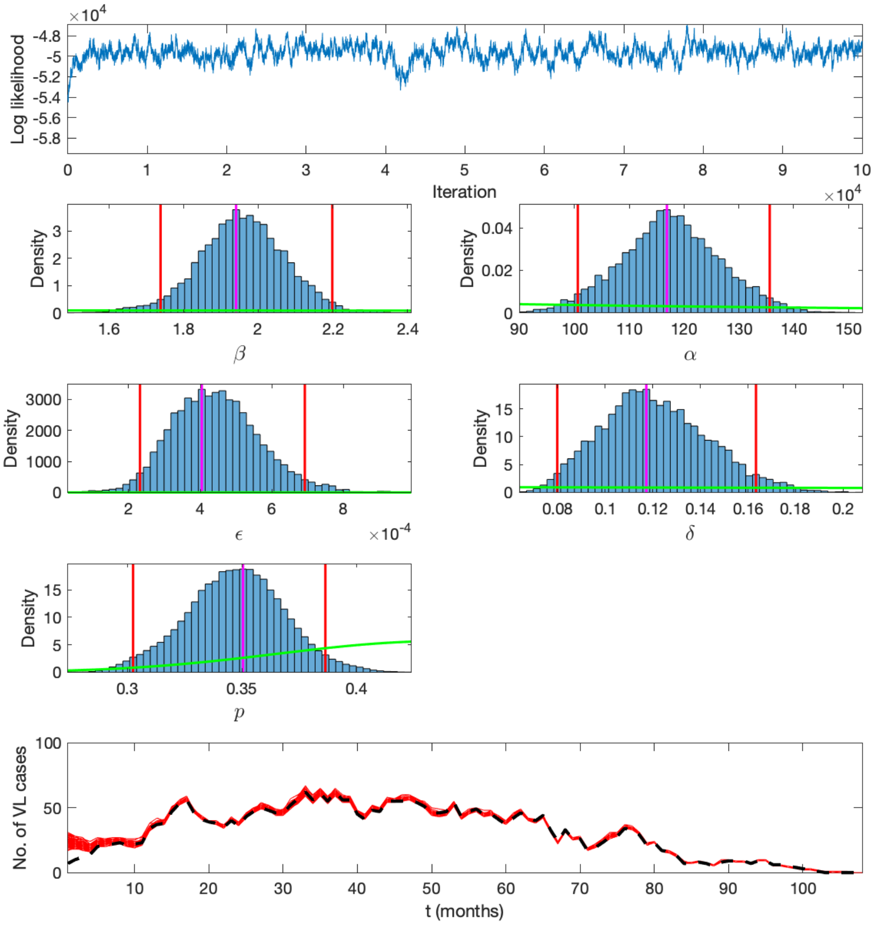

Fig. S6. Output of the MCMC algorithm for the model with additional within-household transmission and 2% asymptomatic and pre-symptomatic infectiousness relative to VL cases. Top: Log-likelihood trace plot. 2nd-4th row: Posterior distributions for the spatial transmission rate constant β (month^{-1}), risk decay distance α (m), background transmission rate ϵ (month^{-1}), additional within-household transmission rate δ (month^{-1}), and incubation period distribution parameter p, with prior distributions (green lines), posterior modes (magenta lines) and 95% highest posterior density intervals (red lines). Bottom: Number of active VL cases over time in 100 iterations of the MCMC algorithm (red lines) with inferred missing onset and recovery times, and number of active cases excluding individuals with missing onset and/or recovery times (black dashed line).

The corresponding autocorrelation plots are shown in Fig. S7. The high degree of autocorrelation evident for all the parameters is due to strong correlation between the transmission parameters and the missing data, in particular between the spatial transmission rate constant β and the asymptomatic infection times. Fig. S8 shows that β is strongly negatively correlated with the mean asymptomatic infection time $\bar{A}$. This is expected since a higher overall transmission rate leads to individuals being infected earlier. With a continuous-time model we could tackle this correlation using a 'non-centred' MCMC algorithm, i.e. by re-parameterising the model to reduce the *a priori* dependence between β and the asymptomatic infection times (29, 32), but with a discrete-time model the re-parameterisation is much more difficult, since the asymptomatic infection times do not vary continuously with β. We therefore ran the MCMC algorithm for a large number of iterations ($N = 10^5$) to obtain a sufficient number of independent samples.

Table S4. Parameter estimates (modes and 95% highest posterior density intervals) for models with different relative pre-symptomatic and asymptomatic infectiousness, h_0 and h_4, and without and with additional within-household transmission, $\delta = 0$ vs $\delta > 0$

Parameter	No additional within-household transmission, $\delta = 0$			Additional within-household transmission, $\delta > 0$		
	$h_0, h_4 = 0$	$h_0, h_4 = 0.01$	$h_0, h_4 = 0.02$	$h_0, h_4 = 0$	$h_0, h_4 = 0.01$	$h_0, h_4 = 0.02$
β (month^{-1})	2.65 (2.32,2.99)	2.51 (2.25,2.82)	2.44 (2.19,2.70)	2.16 (1.95,2.48)	2.08 (1.80,2.31)	1.94 (1.74,2.20)
α (m)	104 (88,120)	100 (88,118)	100 (88,114)	119 (101,140)	115 (103,140)	117 (101,136)
ϵ (10^{-4} month^{-1})	6.7 (3.6,9.5)	6.0 (3.8,9.5)	5.1 (3.3,8.0)	6.1 (3.6,9.0)	5.0 (3.0,7.8)	4.0 (2.3,6.9)
δ (month^{-1})	0	0	0	0.09 (0.07,0.13)	0.10 (0.08,0.15)	0.12 (0.08,0.16)
p	0.41 (0.36,0.45)	0.40 (0.36,0.46)	0.41 (0.36,0.45)	0.37 (0.34,0.41)	0.36 (0.32, 0.39)	0.35 (0.30,0.39)
ΔDIC	2950	3182	3261	955	460	0

ΔDIC is the difference in DIC from the model with $\delta > 0$ and $h_0, h_4 = 0.02$

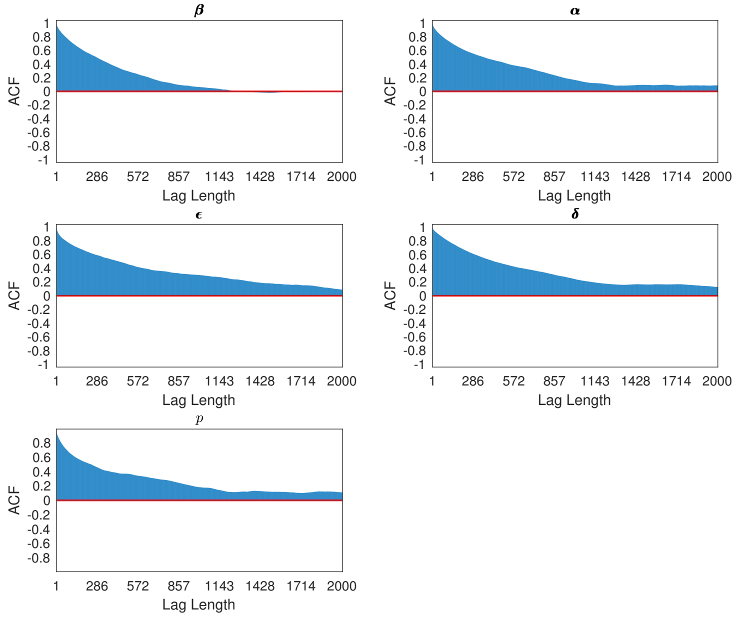

Fig. S7. Autocorrelation plots for the model parameters $\boldsymbol{\theta} = (\beta, \alpha, \epsilon, \delta, p)$ for the model with additional within-household transmission and 2% relative asymptomatic and pre-symptomatic infectiousness, showing the correlation between samples in the MCMC chain at different lags.

Correlations between the model parameters were explored by plotting the pairwise marginal posterior densities (Fig. S9) and calculating the correlation coefficient matrix of the MCMC samples, $\boldsymbol{\rho}(\boldsymbol{\theta}, \boldsymbol{\theta}) = (\mathrm{corr}(\theta_i, \theta_j))_{i,j=1,\ldots,n_{\boldsymbol{\theta}}}$, where $\mathrm{corr}(\mathbf{x}, \mathbf{y}) = \mathrm{cov}(\mathbf{x}, \mathbf{y}) / \sqrt{\mathrm{var}(\mathbf{x})\,\mathrm{var}(\mathbf{y})}$ and $\mathrm{var}(\mathbf{x})$ is the sample variance of $\mathbf{x}$ and $n_{\boldsymbol{\theta}}$ is the dimension of $\boldsymbol{\theta}$. Fig. S9 and the correlation coefficient matrix,

$$\boldsymbol{\rho}(\boldsymbol{\theta}, \boldsymbol{\theta}) = \begin{pmatrix} 1 & 0.02 & -0.45 & -0.09 & -0.05 \\ 0.02 & 1 & -0.40 & -0.18 & 0.31 \\ -0.45 & -0.40 & 1 & 0.08 & -0.11 \\ -0.09 & -0.18 & 0.08 & 1 & -0.55 \\ -0.05 & 0.31 & -0.11 & -0.55 & 1 \end{pmatrix}$$

show that there is some negative correlation between β and ϵ, α and ϵ, and δ and p. These correlations are not surprising: the more transmission that is explained by proximity to infectious individuals (the higher β), the less needs to be explained by the background transmission (the lower ϵ); the flatter the spatial kernel (the larger α), the fewer infections need to be explained by the background transmission; and the more infections are accounted for by transmission within the same household (the higher δ), the longer the incubation period (the lower p) needs to be (due to long times between onsets of cases in the same household).

The acceptance rate for the transmission parameter updates (Step 1 in the MCMC algorithm) was 23.2–23.4% for all models, as expected from tuning the proposal variance (see §2B.5). The acceptance rates for the updates for the pre-symptomatic infection times; missing VL onset and treatment times; and unobserved asymptomatic infection and recovery times were between 56% and 97% for every model, indicating that the algorithm efficiently explores the space of unobserved data.

C. Unobserved pre-symptomatic infection times, and asymptomatic infection and recovery times. Here we present plots of various quantities derived from the posterior distributions of the pre-symptomatic infection times and asymptomatic infection and recovery times to demonstrate what can be inferred about the missing data using the data augmentation algorithm. Fig. S10 shows the incidence curve of VL and PKDL cases for the whole study area and the inferred incidence curve of asymptomatic

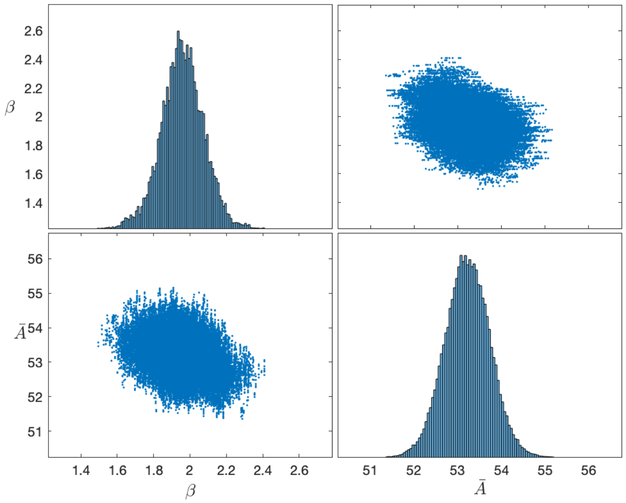

Fig. S8. Correlation between the spatial transmission rate constant β and the mean asymptomatic infection time $\bar{A} = \frac{1}{n_A}\sum_{j=1}^{n_A} A_j$, where $n_A = |\{j : 1 \leq A_j \leq T\}|$ is the number of individuals asymptomatically infected during the study.

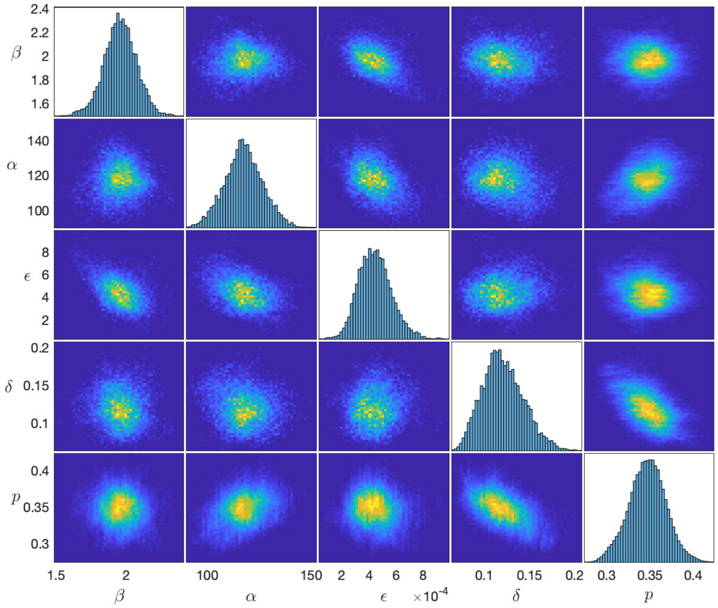

Fig. S9. Posterior distributions and pairwise correlations of the transmission parameters for the model with additional within-household transmission and 2% relative asymptomatic and pre-symptomatic infectiousness. Main diagonal: Marginal posterior distributions of the transmission parameters. Off diagonals: Heatmap plots of pairwise marginal posterior densities showing the correlations between parameters. Lighter yellower colours indicate areas of higher posterior density.

infections (averaged over the MCMC chain). The number of asymptomatic infections increases and decreases with the number of VL cases as expected given the assumption of a fixed incidence ratio of asymptomatic to symptomatic infection.

The posterior probabilities that individuals were asymptomatically infected during the study (shown in Fig. S11, with individuals arranged in order of para and household number) show clustering of asymptomatic infection in space and that this is associated with proximity to VL cases (the probabilities of asymptomatic infection are higher where the VL case density is higher). This is as expected given the estimates of the transmission parameters — the rapid decrease in the risk of infection with distance from an infectious individual (Fig. S19) — and the relative infectiousness of VL cases and asymptomatic individuals.

The examples shown in Fig. S12 demonstrate that the data does provide some information about the asymptomatic "infection" times of non-symptomatic individuals, since the posterior distributions are non-uniform over the range of possible asymptomatic infection times for each individual. They also show that the asymptomatic infection time posterior distributions

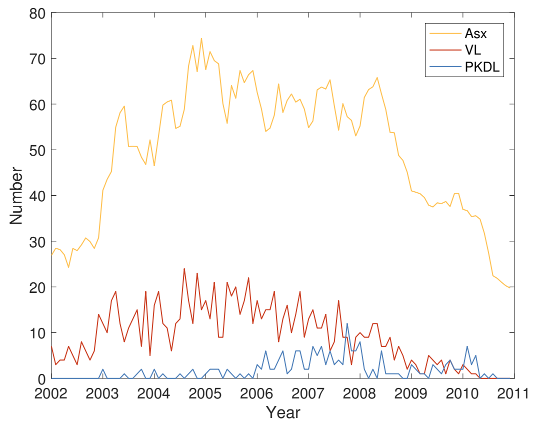

Fig. S10. Numbers of new VL and PKDL cases over time in the study area, with inferred numbers of new asymptomatic infections.

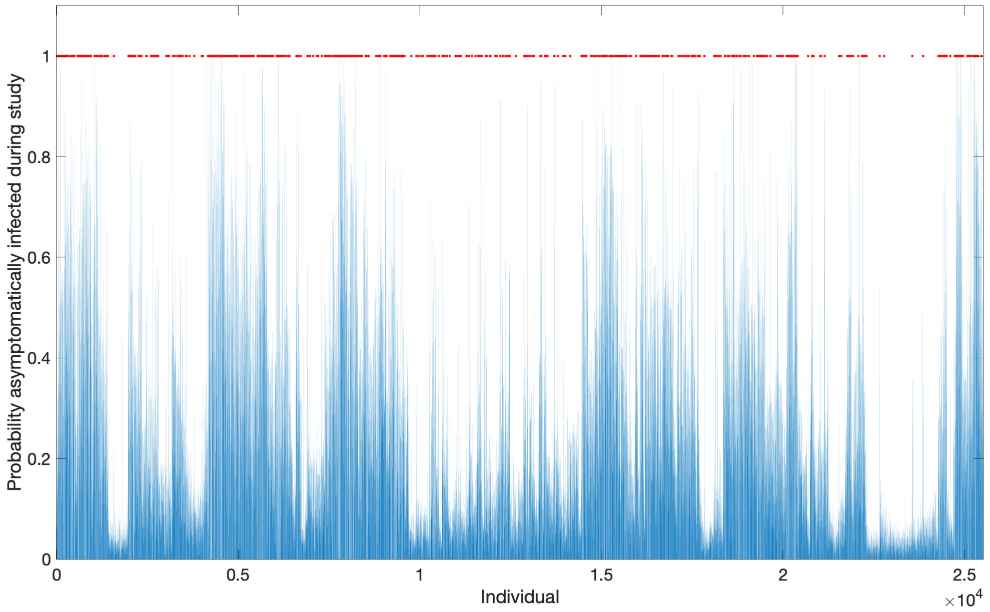

Fig. S11. Posterior probabilities individuals were asymptomatically infected during the study (blue lines). Individuals who had VL onset during the study are marked by red dots.

and proposal distributions (probabilities of asymptomatic infection according to the empirical average of the infection pressure on the individual over the history of the chain) converged with each other. This is as expected since the model parameters converge to the high posterior density part of the parameter space as the chain progresses, such that the running average of the infection pressure on each individual at each time point approaches a constant and the asymptomatic infection times are effectively proposed from their posterior distributions in the long run.

Fig. S13 shows the posterior distributions of the unobserved infection times of a selection of VL cases, and indicates that the data does contain sufficient information to constrain the probable infection times of some cases, as the posterior distributions differ significantly from the prior distributions (i.e. from the prior for the incubation period distribution) for some cases.

D. Reconstructed transmission trees. Snapshots of one inferred transmission tree in the part of the south-east cluster of paras shown in Fig. 3 in the main text at different time points are shown in Fig. S14. These show that while asymptomatic infection (and therefore subsequent immunity) was initially mostly clustered around VL cases, by December 2005 it was widespread and by December 2009 had saturated in this part of the study population. The relatively high uncertainty in infection sources can be seen in the different pattern of infectors and longer inferred transmission distances in this single tree (from one sample from the joint posterior distribution of model parameters and missing data) than the consensus tree in Fig. 3 in the main text.

Fig. S15 shows, for all inferred transmission chains, the relationship between the mean time after the onset of the index case in the chain at which infectees in each infection generation were infected and the mean distance from the index case to infectees in each infection generation (averaged over 1000 sampled transmission trees). It suggests that infection spread over

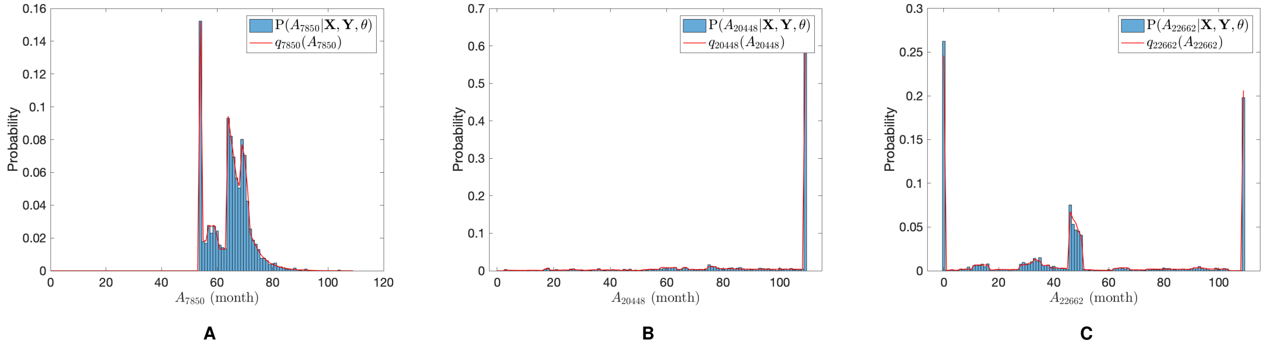

Fig. S12. Posterior distributions of the unobserved asymptomatic infection times of a selection of individuals (blue histograms) and their final asymptomatic infection time proposal distributions (red lines). Note that asymptomatic "infection" in months 0 and $T+1=109$, represent asymptomatic infection before the study and no asymptomatic infection up to the end of the study, respectively. (*A*) Individual who migrated into a house with an active VL case from outside the study area in month 53 and therefore had a high initial probability of asymptomatic infection, followed by further peaks in asymptomatic infection risk in months 64 and 69 with the PKDL and VL onsets of two other household members in months 63 and 68 respectively. (*B*) Individual born in month 2 with a high probability of having avoided asymptomatic infection for the duration of the study. (*C*) Individual who was 23-years-old at the start of the study with a moderately high risk of having been asymptomatically infected before the study and a small peak in asymptomatic infection risk when a fellow household member had VL onset in month 45.

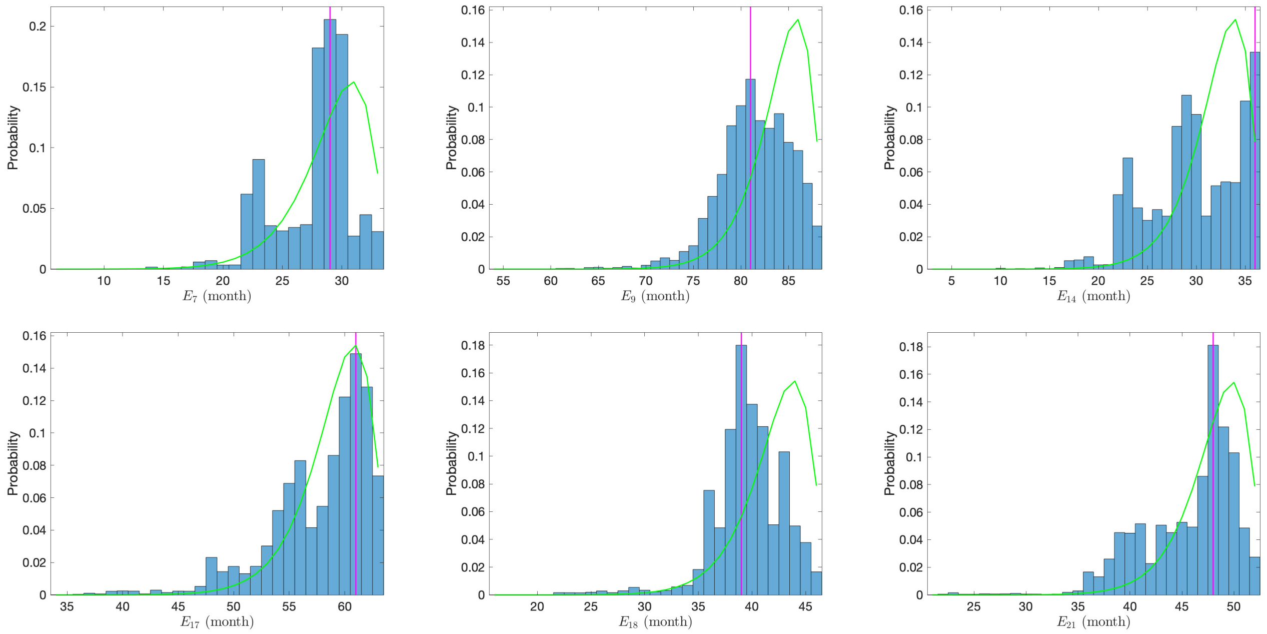

Fig. S13. Posterior distributions of the unobserved infection times of a selection of VL cases with known onset times (blue histograms), with prior distributions (green lines) and modes (magenta lines).

large distances from index cases (up to ∼500m) even within early infection generations, since the infection generations show considerable horizontal spread on the plot.

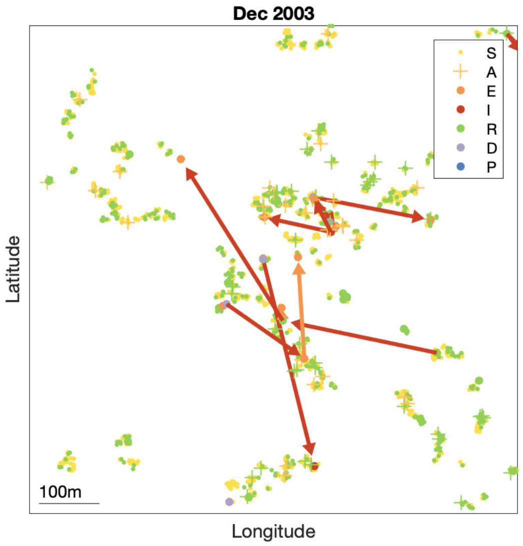

A

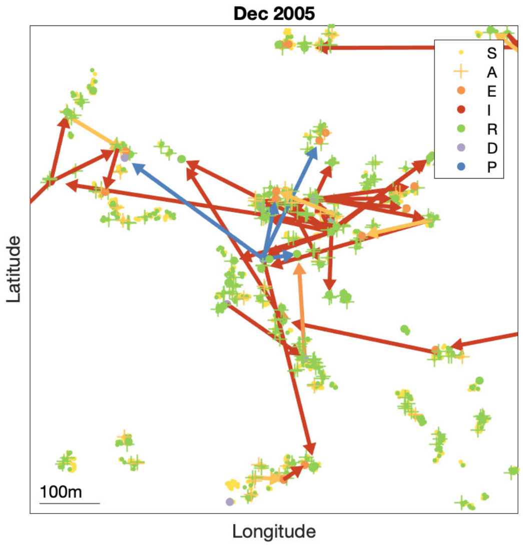

B

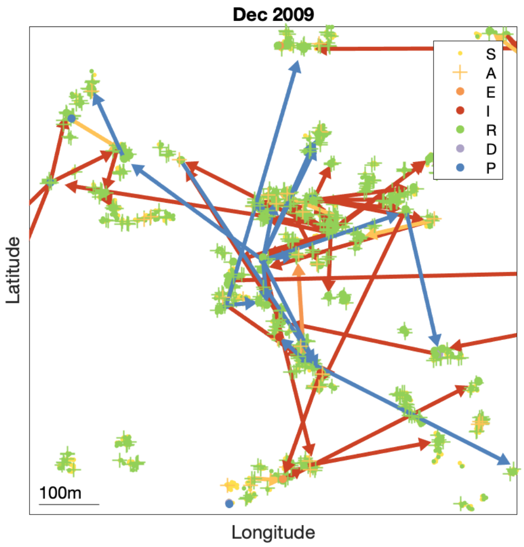

C

Fig. S14. Inferred transmission tree in part of the south-east cluster of villages at different stages of the epidemic — (*A*) December 2003, (*B*) December 2005, and (*C*) December 2009 — from a single sample from the joint posterior distribution of model parameters and missing data. Model parameters chosen close to the posterior mode ($\beta = 1.93\,\text{month}^{-1}$, $\alpha = 120\,\text{m}$, $\epsilon = 3.9 \times 10^{-4}\,\text{month}^{-1}$, $\delta = 0.11\,\text{month}^{-1}$, $p = 0.34$). Dots show susceptible individuals/symptomatic cases and crosses asymptomatic individuals, and are coloured by infection state (see key). Arrows show the source of infection for each VL case infected up to that point in time and are coloured by type of infection source. GPS locations of individuals are jittered slightly so that individuals from the same household are more visible. An animated version showing all months is provided in Movie S2.

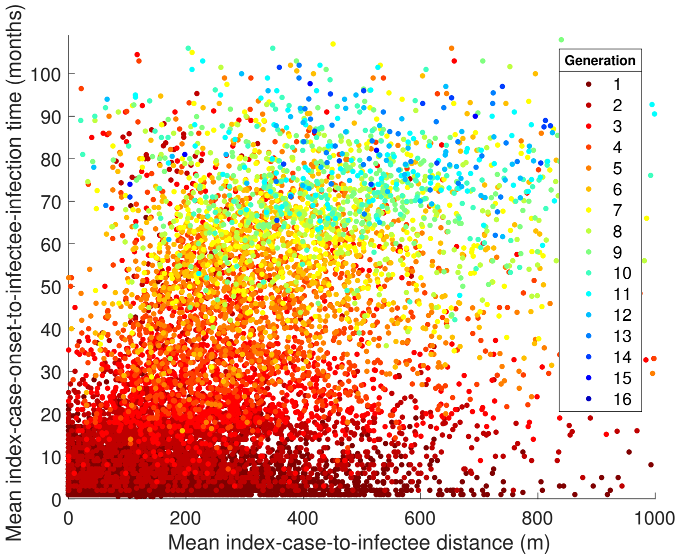

Fig. S15. Relationship between mean index-case-onset-to-infectee-infection time and mean index-case-to-infectee distance by infection generation (see key) for transmission chains from 1000 sampled transmission trees. Some index-case-to-infectee distances are large even within early generations of transmission chains.

9. MCMC Validation

The MCMC algorithm was run on simulated data to verify that it can accurately infer the parameter values and missing data. Since a high proportion of infections are asymptomatic, there is a large amount of missing data in the form of the missing asymptomatic infection and recovery times. To test the ability of the algorithm to infer this missing data and simultaneously recover the true parameter values, we simulated transmission in paras 1–4 (comprising 6231 individuals) from January 2002 to December 2010 with three different proportions of symptomatic infection, $p_I \in \{0.1, 0.15, 0.2\}$, and ran the inference procedure on each of the simulated datasets with the asymptomatic infection data removed, assuming the symptomatic proportion was known. The values of p_I were chosen to cover a plausible range based on the highest cumulative VL incidence in any of the paras over the study period (∼10%, which equates to a maximum possible proportion of asymptomatic infection of ∼90% if the whole population was infected) and estimates from previous studies (in which the highest estimated symptomatic proportion was 20%) (52). To ensure relatively consistent VL incidence across the simulated datasets and to account for potential correlation between p_I and other parameter values, we ran the MCMC algorithm on the observed data for paras 1–4 for each value of p_I and re-simulated the data with the simulation model using parameter values close to the posterior modes obtained. There were 242, 303 and 331 VL cases in the simulations with $p_I = 0.1$, 0.15 and 0.2 respectively.

Fig. S16 shows the posterior distributions for the parameters obtained from running the MCMC algorithm on the simulated datasets. All of the posterior distributions overlap the true parameter values, indicating that the algorithm is able to recover the true parameter values, even for proportions of asymptomatic infection as high as 90%. The algorithm can also accurately estimate the number of new asymptomatic infections each month across the range of symptomatic proportions tested, as shown by the inferred epidemic curves of asymptomatic infections in Fig. S17.

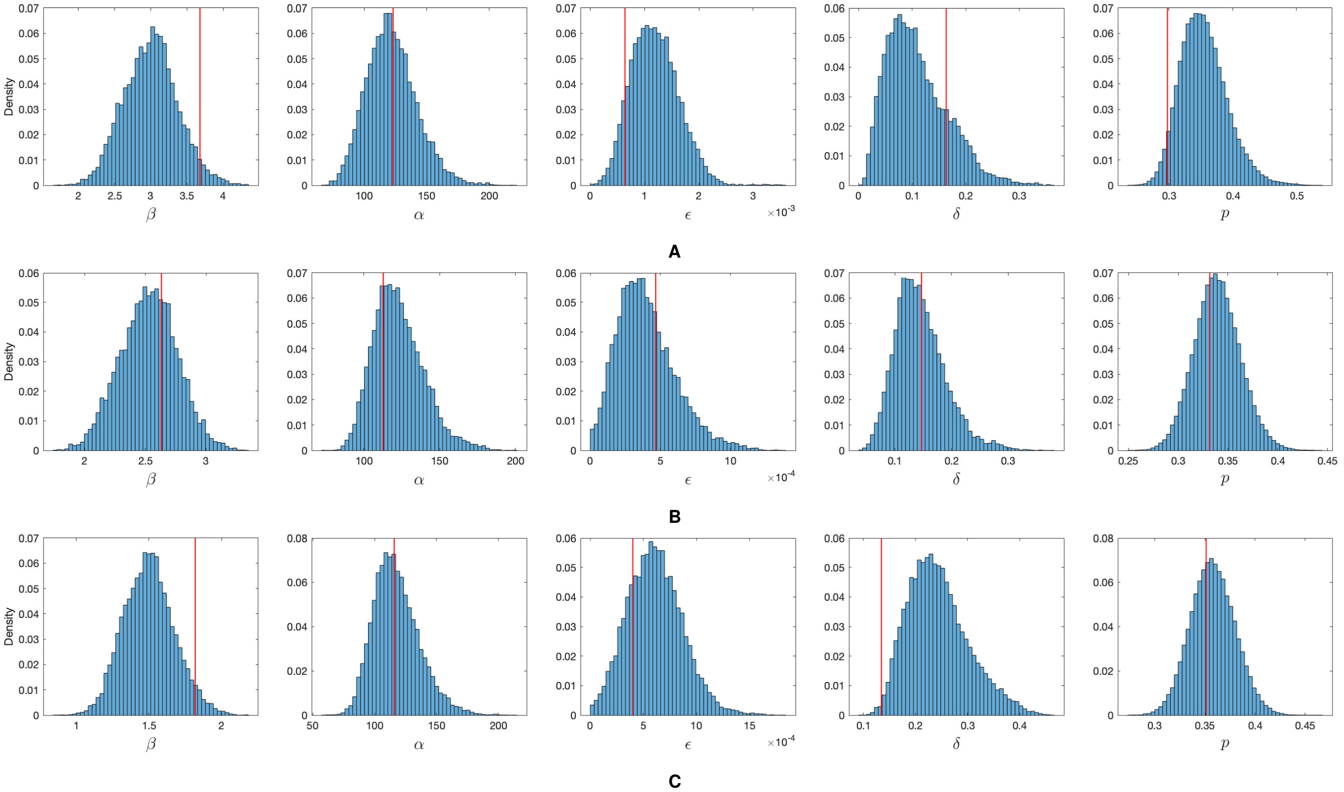

Fig. S16. Validation of the MCMC algorithm. Posterior distributions of the model parameters obtained from running the inference procedure for 10^5 iterations on simulated datasets for paras 1–4 with different symptomatic proportions: (*A*) $p_I = 0.1$, (*B*) $p_I = 0.15$, (*C*) $p_I = 0.2$. True parameter values (shown by the red lines) chosen as values close to the posterior modes estimated from fitting to the observed data: (A) $\beta = 3.68\,\text{month}^{-1}$, $\alpha = 123\,\text{m}$, $\epsilon = 6.4 \times 10^{-4}\,\text{month}^{-1}$, $\delta = 0.16\,\text{month}^{-1}$, $p = 0.30$, (B) $\beta = 2.63\,\text{month}^{-1}$, $\alpha = 113\,\text{m}$, $\epsilon = 4.7 \times 10^{-4}\,\text{month}^{-1}$, $\delta = 0.15\,\text{month}^{-1}$, $p = 0.33$, (C) $\beta = 1.82\,\text{month}^{-1}$, $\alpha = 116\,\text{m}$, $\epsilon = 4.0 \times 10^{-4}\,\text{month}^{-1}$, $\delta = 0.13\,\text{month}^{-1}$, $p = 0.35$.

10. Validation of DIC for Model Comparison

As it is known that there can be issues with using DIC to compare models, particularly in the context of missing data (40, 41, 53), we verified that the version of DIC for latent variable models that we use (Eq. (S24)) can discriminate between models with/without additional within-household transmission using simulated data. We simulated transmission in paras 1 to 4 from January 2002 to December 2010 without and with additional within-household transmission $\delta = 0\,\text{month}^{-1}$ and $\delta = 0.15\,\text{month}^{-1}$ (and $h_0, h_4 = 0.02$), and then ran the MCMC algorithm and DIC calculation on the simulated datasets with

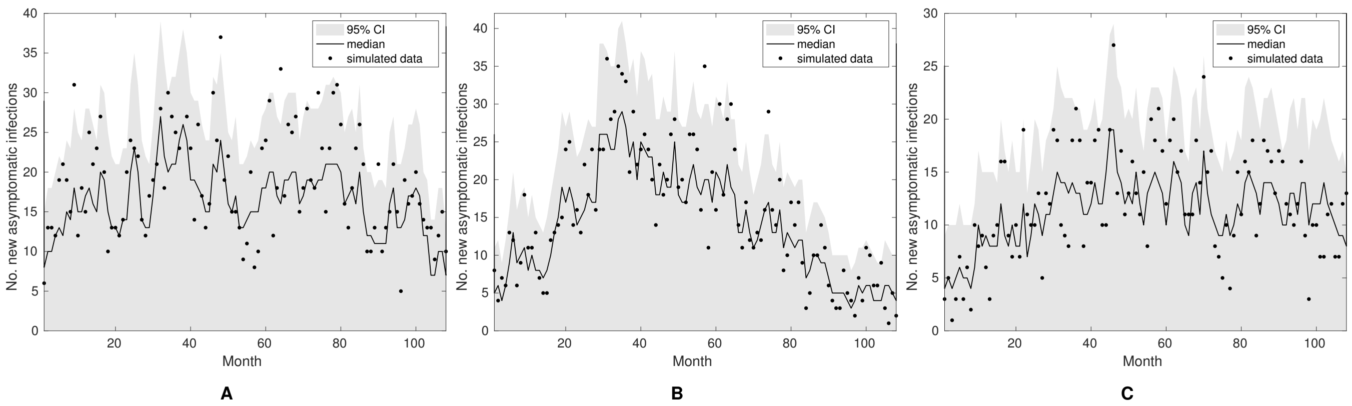

Fig. S17. Simulated (black dots) and inferred (black line = median, grey shading = 95% CI) epidemic curves of asymptomatic infections for paras 1–4 with different symptomatic proportions (*A*) $p_I = 0.1$, (*B*) $p_I = 0.15$, (*C*) $p_I = 0.2$. Parameter values as in Fig. S16.

$\delta = 0\,\text{month}^{-1}$ and $\delta = 0.15\,\text{month}^{-1}$. In both cases the DIC measure correctly identified the true underlying model (Table S5). These results agree with the findings of Gamado *et al.* (53) that the DIC defined in Eq. (S24) can be used to distinguish different forms of spatial kernel.

Table S5. Validation of use of DIC for identifying whether there is additional within-household transmission, $\delta = 0$ vs $\delta > 0$. Values shown are differences in DIC from the best-fitting model for each true value of δ, which is shown in bold.

Assumed	True	
	$\delta = 0$	$\delta = 0.15$
$\delta = 0$	**0**	390
$\delta = 0.15$	59	**0**

Table S6. Para-level populations and VL and PKDL incidence 2002–2010

Para	Cluster	Total no. individuals	Mean population	Total VL cases	Total PKDL cases	Mean VL incidence (cases/10,000/yr)	Mean PKDL incidence (cases/10,000/yr)
1	NW	1398	1219	120	11	109	10
2	NW	1650	1376	46	3	37	2
3	NW	998	830	18	3	24	4
4	NW	2185	1768	197	35	124	22
5	NW	934	774	15	2	22	3
6	NW	1640	1363	94	22	77	18
7	SE	604	493	42	7	95	16
8	SE	585	496	8	0	18	0
9	SE	969	809	33	6	45	8
10	SE	701	595	17	3	32	6
11	SE	1300	1080	28	9	29	9
12	SE	2807	2388	102	25	47	12
13	SE	933	816	36	7	49	10
14	SE	446	391	23	3	65	9
15	SE	660	574	15	3	29	6
16	NW	905	762	26	9	38	13
17	NW	2080	1764	75	13	47	8
18	NW	3212	2653	61	11	26	5
19	NW	774	647	62	18	107	31
Total		24781	20798	1018	190	54	10

NW = north-west, SE = south-east.

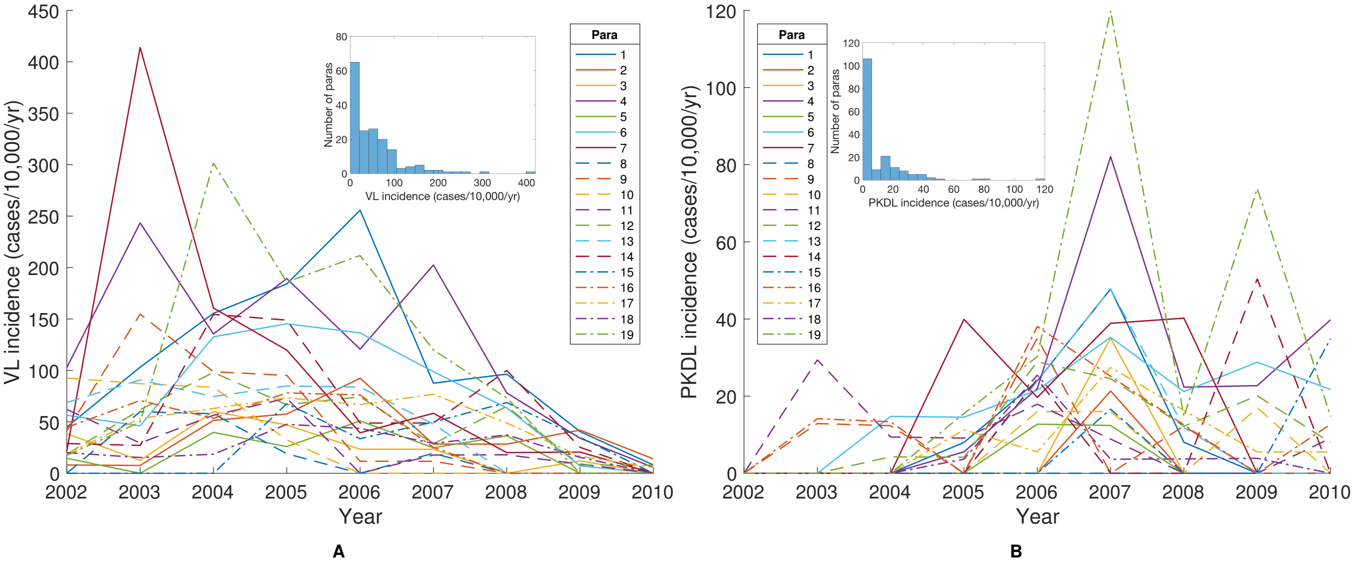

Fig. S18. Para-level annual incidence of (*A*) VL and (*B*) PKDL. Insets: distributions of annual incidences.

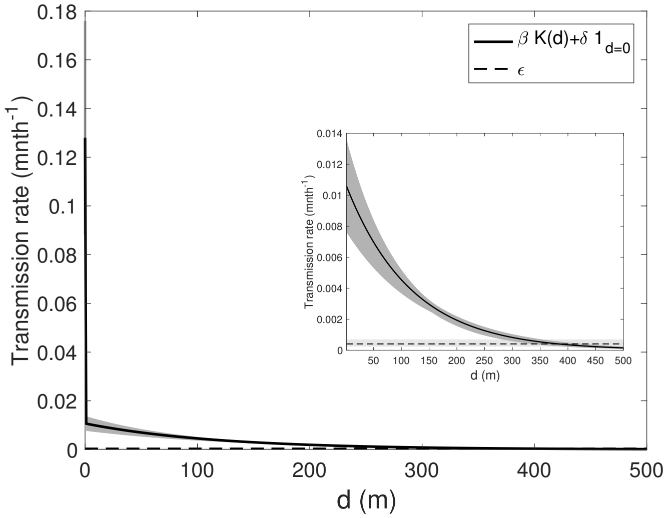

Fig. S19. Estimated spatial transmission kernel showing decrease in infection pressure with distance from an infectious individual. Transmission rate per month, $\beta K(d)$, shown as a function of distance d away from a VL case (solid black line = mode, dark grey shaded region = 95% CI). Background transmission rate, ϵ, for unexplained infections also shown for comparison (dashed black line = mode, light grey shaded region = 95% CI). Inset: enlarged view of the part of the transmission kernel outside the household of the VL case.

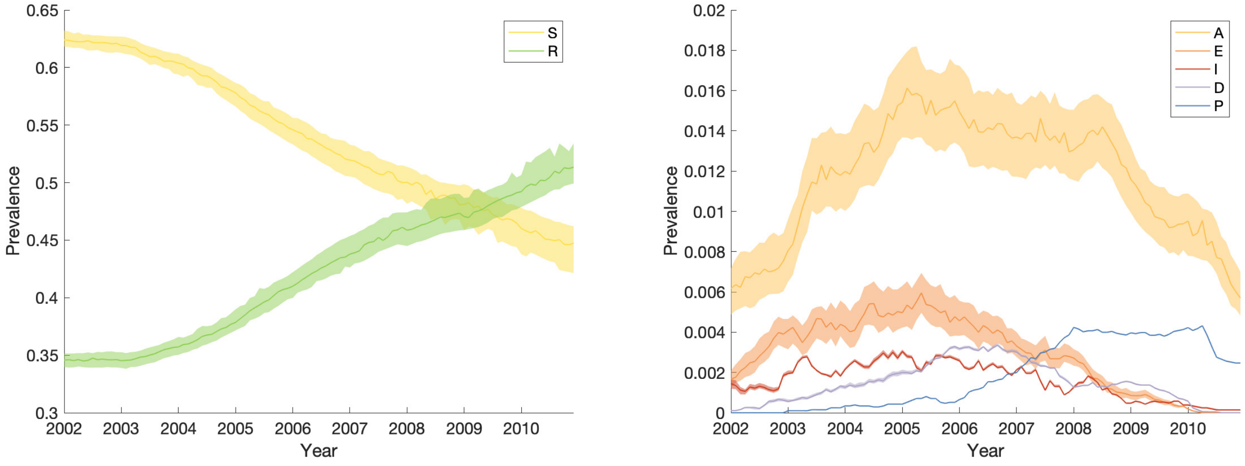

A **B**

Fig. S20. Inferred prevalences of the different infection states in the model over time: (*A*) susceptible (S) and recovered (R), (*B*) asymptomatically infected (A), pre-symptomatically infected (E), symptomatic VL (I), dormant infection (D), symptomatic PKDL (P). Solid lines show modes, shaded bands show 95% CIs. Note the difference in vertical axis scales.

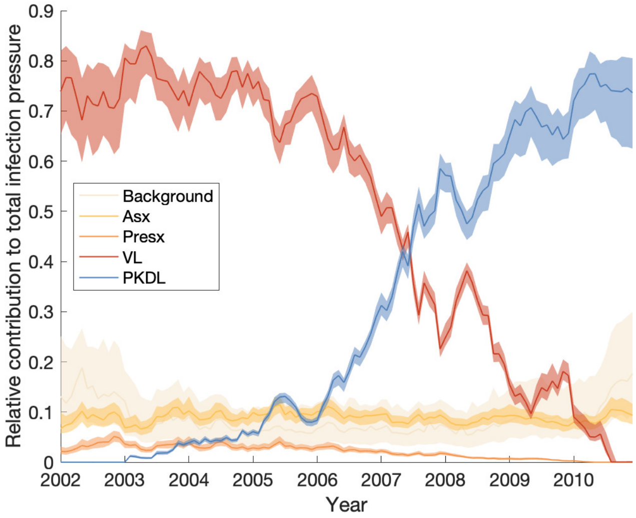

Fig. S21. Relative contributions of background transmission, asymptomatic (Asx) individuals, pre-symptomatic (Presx) individuals, VL cases and PKDL cases to the total infection pressure on susceptible individuals. Solid lines show modes, shaded bands show 95% CIs.

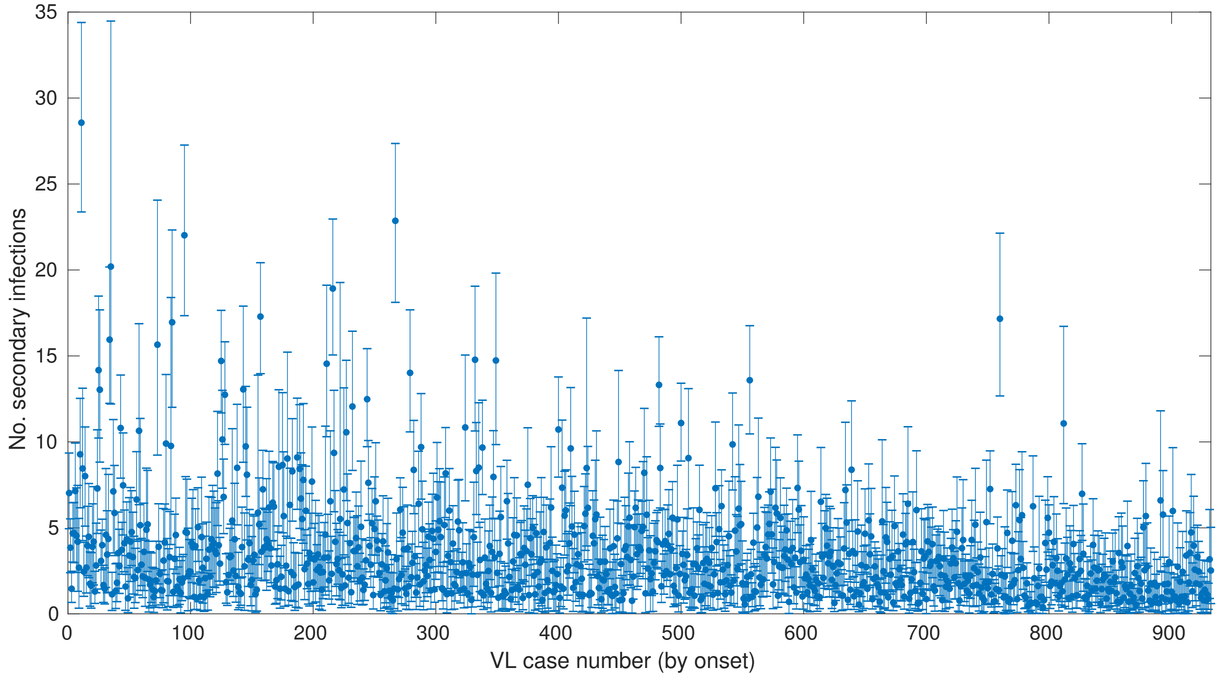

A

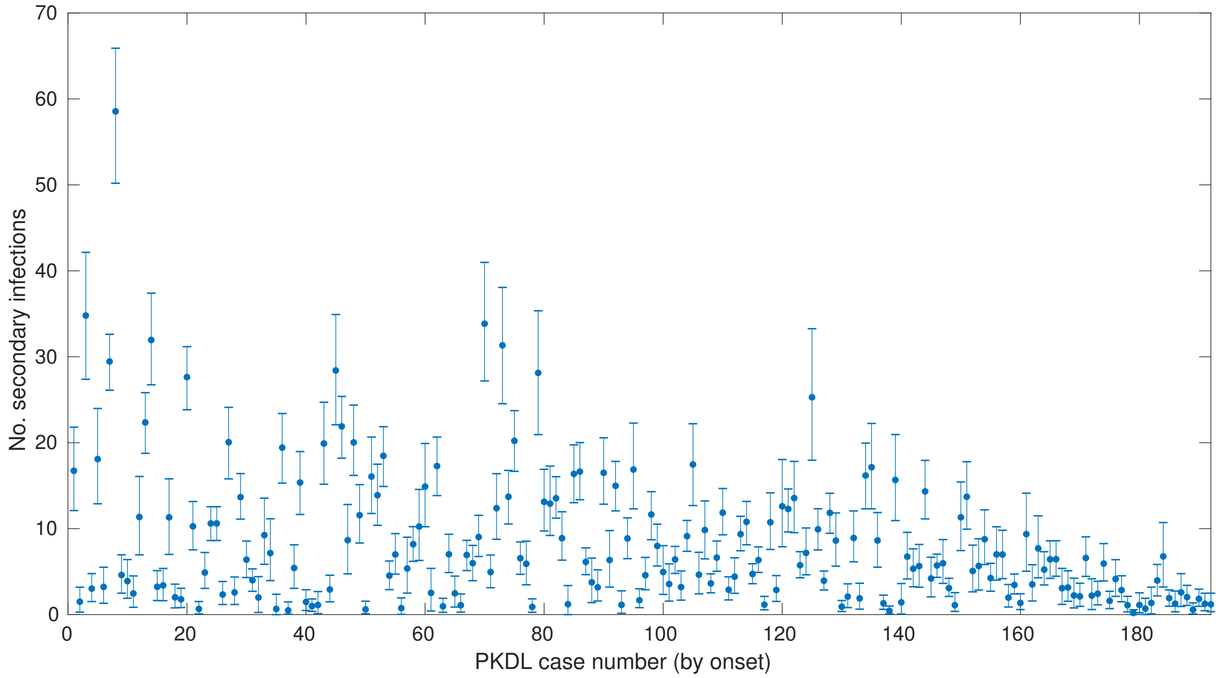

B

Fig. S22. Number of secondary infections generated by (A) each VL case and (B) each PKDL case. Dots show mean reproduction number, bars show 95% CI (2.5–97.5% quantile).

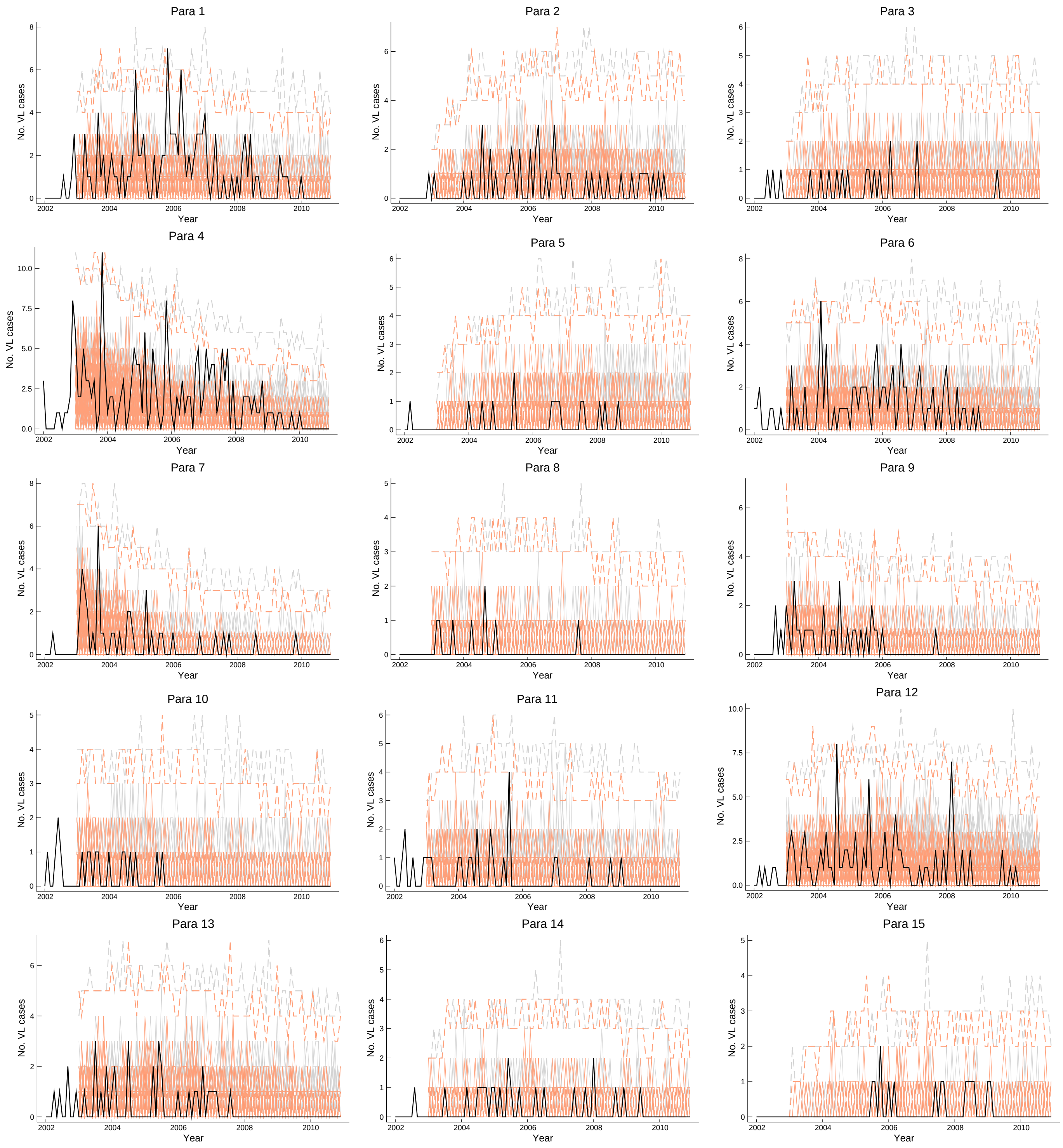
Para 1
Para 2
Para 3
Para 4
Para 5
Para 6
Para 7
Para 8
Para 9
Para 10
Para 11
Para 12
Para 13
Para 14
Para 15
No. VL cases
Year
2002
2004
2006
2008
2010

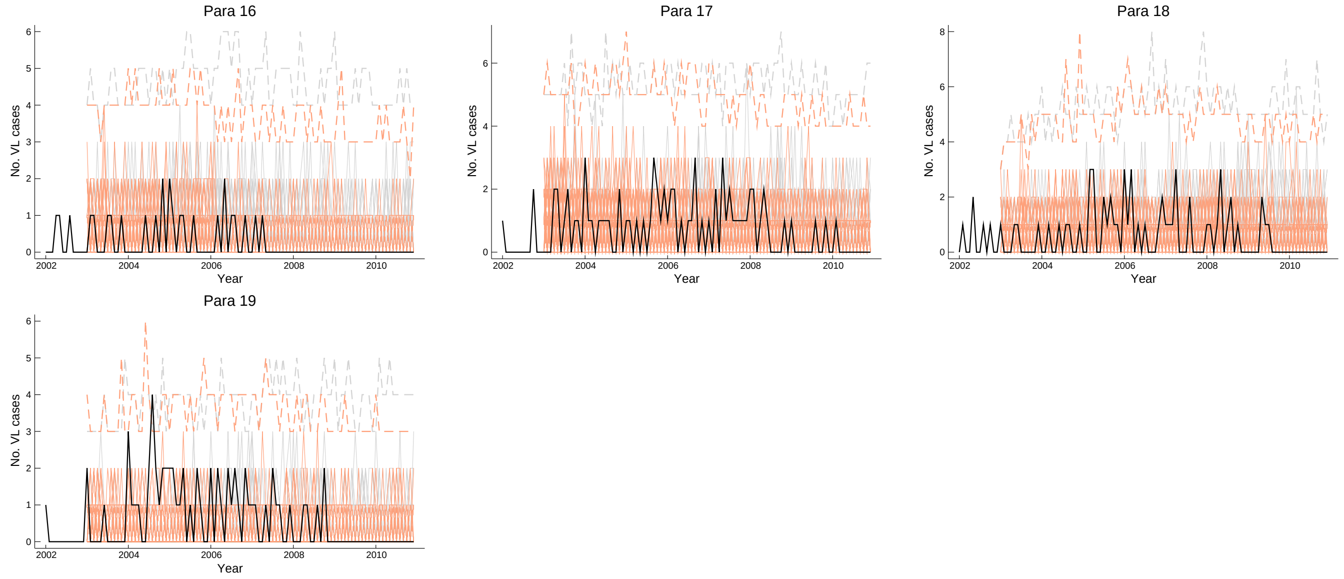

Fig. S23. Para-level epidemic curves simulated with the individual-level spatiotemporal transmission model starting from January 2003 (by which point all but one para had had at least 1 VL case since January 2002) under normal conditions (solid grey lines) (17% of VL cases develop PKDL and 0.3% of asymptomatic individuals develop PKDL) and with complete prevention of PKDL (solid pink lines). Solid coloured lines show 100 randomly chosen simulations from 10,000 simulations (100 samples of the model parameters and initial infection statuses from the posterior distribution each simulated 100 times), solid black lines show observed VL incidence, dashed lines show the minimums and maximums of all the simulations at each time point.

Table S7. Observed and simulated numbers of VL cases in each para for different PKDL interventions 2002–2010

Para	Observed VL cases	Simulated VL cases under different PKDL interventions (mean (95% CI))				
		Normal interventions	Complete prevention of PKDL	Difference	Mean PKDL duration halved	Difference
1	120	49 (19,86)	37 (15,68)	-12 (-54,29)	45 (18,79)	-4 (-49,40)
2	46	36 (9,71)	24 (6,53)	-12 (-52,26)	31 (8,66)	-5 (-48,38)
3	18	24 (7,52)	19 (5,41)	-6 (-37,22)	22 (6,48)	-2 (-34,29)
4	197	119 (81,160)	92 (62,128)	-27 (-75,21)	109 (74,147)	-11 (-61,40)
5	15	24 (6,50)	16 (3,38)	-8 (-38,19)	21 (5,46)	-3 (-34,28)
6	94	66 (33,101)	43 (20,74)	-23 (-65,19)	58 (29,91)	-8 (-53,37)
7	42	33 (18,50)	27 (15,42)	-6 (-26,13)	31 (17,47)	-3 (-23,17)
8	8	10 (2,26)	8 (2,22)	-1 (-19,14)	9 (2,25)	0 (-18,17)
9	33	24 (12,42)	20 (10,35)	-4 (-24,14)	23 (11,39)	-1 (-22,18)
10	17	25 (13,43)	17 (8,29)	-9 (-27,8)	22 (11,38)	-4 (-24,16)
11	28	36 (18,59)	22 (11,39)	-14 (-40,10)	31 (15,53)	-6 (-33,21)
12	102	91 (43,145)	70 (33,114)	-21 (-84,38)	84 (39,137)	-7 (-73,59)
13	36	44 (17,75)	33 (13,60)	-11 (-47,25)	40 (15,69)	-4 (-43,35)
14	23	14 (3,30)	11 (2,24)	-3 (-21,14)	13 (3,28)	-1 (-20,17)
15	15	5 (0,17)	5 (0,14)	-1 (-13,10)	5 (0,15)	0 (-13,11)
16	26	34 (15,58)	22 (9,40)	-13 (-39,13)	29 (12,52)	-5 (-34,24)
17	75	49 (22,82)	39 (18,66)	-10 (-48,25)	45 (21,77)	-3 (-43,37)
18	61	46 (18,86)	37 (15,69)	-9 (-50,30)	43 (17,80)	-3 (-47,40)
19	62	20 (6,42)	16 (5,34)	-4 (-29,18)	19 (6,39)	-2 (-28,23)
Total	1018	752 (594,926)	557 (425,701)	-194 (-362,-36)	679 (530,839)	-73 (-245,96)

Movie S1. Animation of the evolution of the most likely transmission tree in Fig. 3 in the main text over the course of the epidemic from January 2003 ($t = 13$) to December 2010 ($t = 108$). Dots show individuals coloured by their infection state (see key). Arrows show the most likely source of infection for each case over 1000 sampled transmission trees, coloured by type of infection source and shaded according to the proportion of trees in which that individual was the most likely infector (darker shading indicating a higher proportion).

Movie S2. Animation of the transmission tree shown in Fig. S14 from a single sample from the joint posterior distribution of model parameters and missing data. Dots, crosses and arrows as in Fig. S14.

References

1. LAC Chapman, et al., The role of case proximity in transmission of visceral leishmaniasis in a highly endemic village in Bangladesh. *PLoS Neglected Trop. Dis.* **12**, e0006453 (2018).
2. A Stauch, et al., Visceral Leishmaniasis in the Indian Subcontinent: Modelling Epidemiology and Control. *PLoS Neglected Trop. Dis.* **5**, 1–12 (2011).
3. EA Le Rutte, et al., Feasibility of eliminating visceral leishmaniasis from the Indian subcontinent: explorations with a set of deterministic age-structured transmission models. *Parasites & Vectors* **9**, 24 (2016).
4. EA Le Rutte, et al., Elimination of visceral leishmaniasis in the Indian subcontinent: a comparison of predictions from three transmission models. *Epidemics* **18**, 67–80 (2017).
5. C Bern, et al., Risk factors for kala-azar in Bangladesh. *Emerg. Infect. Dis.* **11**, 655–662 (2005).
6. S Rijal, et al., Increasing Failure of Miltefosine in the Treatment of Kala-azar in Nepal and the Potential Role of Parasite Drug Resistance, Reinfection, or Noncompliance. *Clin. Infect. Dis.* **56**, 1530–1538 (2013).
7. K Rai, et al., Single locus genotyping to track Leishmania donovani in the Indian subcontinent: Application in Nepal. *PLoS Neglected Trop. Dis.* **11**, e0005420 (2017).
8. C Bern, et al., Loss of leishmanin skin test antigen sensitivity and potency in a longitudinal study of visceral leishmaniasis in Bangladesh. *Am. J. Trop. Medicine Hyg.* **75**, 744–8 (2006).
9. LAC Chapman, et al., Age trends in asymptomatic and symptomatic Leishmania donovani infection in the Indian subcontinent: a review of data from diagnostic and epidemiological studies. *PLoS Neglected Trop. Dis.* **12**, e0006803 (2018).
10. D Dinesh, A Ranjan, A Palit, K Kishore, SK Kar, Seasonal and nocturnal landing/biting behaviour of Phlebotomus argentipes (Diptera: Psychodidae). *Annals Trop. Medicine & Parasitol.* **95**, 197–202 (2001).
11. K Ghosh, S Das, A Hati, Studies on seasonal man sandfly (phlebotomus argentipes) contact at night. *J. Indian Assoc. for Commun. Dis.* **5**, 14–18 (1982).
12. R Srinivasan, K Panicker, Biting rhythm & biting activity of phlebotomid sandflies. *Indian J. Med. Res.* **95**, 301–304 (1992).
13. S Shrestha, S Pant, Seasonal distribution of phlebotomine sand flies-vector of visceral leishmaniasis. *J. Nepal Med. Assoc.* **32**, 237–246 (1994).
14. AK Hati, et al., Phlebotomus Argentipes Annandale and Brunetti (Diptera) caught on man baits at night in a clean biotope. *Rec. Zool. Surv. India* **81**, 9–12 (1984).
15. DM Poché, Z Torres-Poché, R Garlapati, T Clarke, RM Poché, Short-term movement of Phlebotomus argentipes (Diptera: Psychodidae) in a visceral leishmaniasis-endemic village in Bihar, India. *Journal of Vector Ecology* **43**, 285–292 (2018).
16. R Mandal, P Das, V Kumar, S Kesari, Spatial Distribution of Phlebotomus argentipes (Diptera: Psychodidae) in Eastern India, a Case Study Evaluating Multispatial Resolution Remotely Sensed Environmental Evidence and Microclimatic Data. *J. Med. Entomol.* **54**, 844–853 (2017).
17. NS Singh, D Phillips-Singh, Relative abundance of Phlebotominae sandflies with emphasis on vectors of kala-azar. *Asian Pac. J. Trop. Medicine* **3**, 270–271 (2010).
18. S Sundar, Transmission dynamics of visceral leishmaniasis in India: role of asymptomatically infected individuals. American Society of Tropical Medicine and Hygiene: 68th Annual Meeting, Nov 20-24, 2019 (https://www.astmh.org/ASTMH/media/2019-Annual-Meeting/ASTMH-2019-Abstract-Book.pdf) (2019).
19. EA Le Rutte, et al., Policy recommendations from transmission modelling for the elimination of visceral leishmaniasis in the Indian subcontinent. *Clin. Infect. Dis.* **66**, S301–S308 (2018).
20. D Mondal, et al., Quantifying the infectiousness of post-kala-azar dermal leishmaniasis towards sandflies. *Clin. Infect. Dis.* **69**, 251–258 (2019).
21. S Islam, et al., Clinical and immunological aspects of post-kala-azar dermal leishmaniasis in Bangladesh. *Am. J. Trop. Medicine Hyg.* **89**, 345–353 (2013).
22. S Verma, et al., Quantification of parasite load in clinical samples of leishmaniasis patients: Il-10 level correlates with parasite load in visceral leishmaniasis. *PLoS ONE* **5** (2010).
23. M Sudarshan, JL Weirather, ME Wilson, S Sundar, Study of parasite kinetics with antileishmanial drugs using real-time quantitative PCR in Indian visceral leishmaniasis. *J. Antimicrob. Chemother.* **66**, 1751–1755 (2011).
24. K Rai, et al., Relapse after treatment with Miltefosine for visceral Leishmaniasis is associated with increased infectivity of the infecting Leishmania donovani strain. *mBio* **4**, 7–10 (2013).
25. S Moulik, et al., Monitoring of Parasite Kinetics in Indian Post–Kala-azar Dermal Leishmaniasis. *Clin. Infect. Dis.* **66**, 1–7 (2017).
26. LAC Chapman, et al., Quantification of the natural history of visceral leishmaniasis and consequences for control. *Parasites & Vectors* **8**, 521 (2015).
27. TP Dorlo, TA Eggelte, PJ de Vries, JH Beijnen, Characterization and identification of suspected counterfeit miltefosine capsules. *Analyst* **137**, 1265–1274 (2012).
28. CP Jewell, T Kypraios, RM Christley, GO Roberts, A novel approach to real-time risk prediction for emerging infectious diseases: A case study in Avian Influenza H5N1. *Prev. Vet. Medicine* **91**, 19–28 (2009).
29. T Kypraios, Ph.D. thesis (Lancaster University) (2007).
30. GJ Gibson, E Renshaw, Estimating parameters in stochastic compartmental models using markov chain methods. *Math.*

Medicine Biol. **15**, 19–40 (1998).
31. PD O'Neill, GO Roberts, Bayesian inference for partially observed stochastic epidemics. *J. Royal Stat. Soc. A* **162**, 121–129 (1999).
32. CP Jewell, T Kypraios, P Neal, GO Roberts, Bayesian Analysis for Emerging Infectious Diseases. *Bayesian Analysis* **4**, 465–496 (2009).
33. JL Zelner, BA Lopman, AJ Hall, S Ballesteros, BT Grenfell, Linking Time-Varying Symptomatology and Intensity of Infectiousness to Patterns of Norovirus Transmission. *PLoS ONE* **8**, 1–8 (2013).
34. GO Roberts, JS Rosenthal, Coupling and ergodicity of adaptive Markov chain Monte Carlo algorithms. *J. Appl. Probab.* **44**, 458–475 (2007).
35. H Haario, E Saksman, J Tamminen, An Adaptive Metropolis Algorithm. *Bernoulli* **7**, 223 (2001).
36. GO Roberts, A Gelman, WR Gilks, Weak convergence and optimal scaling of random walk Metropolis algorithms. *The Annals Appl. Probab.* **7**, 110–120 (1997).
37. GO Roberts, JS Rosenthal, Optimal scaling for various Metropolis-Hastings algorithms. *Stat. Sci.* **16**, 351–367 (2001).
38. GO Roberts, JS Rosenthal, Examples of Adaptive MCMC. *J. Comput. Graph. Stat.* **18**, 349–367 (2009).
39. DJ Spiegelhalter, NG Best, BP Carlin, A van der Linde, Bayesian Measures of Model Complexity and Fit. *J. Royal Stat. Soc. Ser. B (Statistical Methodol.* **64**, 583–639 (2002).
40. G Celeux, F Forbes, Deviance information criteria for missing data models. *Bayesian Analysis* **1**, 651–673 (2006).
41. DJ Spiegelhalter, NG Best, BP Carlin, AVD Linde, The deviance information criterion: 12 years on (with discussion). *J. Royal Stat. Soc. Ser. B* **64**, 485–493 (2014).
42. S Cauchemez, NM Ferguson, Methods to infer transmission risk factors in complex outbreak data. *J. Royal Soc. Interface* **9**, 456–469 (2012).
43. J Wallinga, P Teunis, Different epidemic curves for severe acute respiratory syndrome reveal similar impacts of control measures. *Am. J. Epidemiol.* **160**, 509–516 (2004).
44. PGT Walker, et al., A Bayesian Approach to Quantifying the Effects of Mass Poultry Vaccination upon the Spatial and Temporal Dynamics of H5N1 in Northern Vietnam. *PLoS Comput. Biol.* **6**, e1000683 (2010).
45. MJ Keeling, P Rohani, *Modeling infectious diseases in humans and animals.* (Princeton University Press), (2011).
46. P Touloupou, Ph.D. thesis (University of Warwick) (2016).
47. MATLAB 9.3.0 (R2017b). The MathWorks, Inc. (https://www.mathworks.co.uk/) (2017).
48. J Bezanson, A Edelman, S Karpinski, VB Shah, Julia: A fresh approach to numerical computing. *SIAM Rev.* **59**, 65–98 (2017).
49. Julia v1.0.5. The Julia Project (https://julialang.org) (2019).
50. T Yangzom, et al., Endemic transmission of visceral leishmaniasis in Bhutan. *Am. J. Trop. Medicine Hyg.* **87**, 1028–37 (2012).
51. B Ostyn, et al., Transmission of Leishmania donovani in the Hills of Eastern Nepal, an Outbreak Investigation in Okhaldhunga and Bhojpur Districts. *PLoS Neglected Trop. Dis.* **9**, e0003966 (2015).
52. S Hirve, et al., Transmission Dynamics of Visceral Leishmaniasis in the Indian Subcontinent – A Systematic Literature Review. *PLoS Neglected Trop. Dis.* **10**, e0004896 (2016).
53. K Gamado, G Marion, T Porphyre, Data-Driven Risk Assessment from Small Scale Epidemics: Estimation and Model Choice for Spatio-Temporal Data with Application to a Classical Swine Fever Outbreak. *Front. Vet. Sci.* **4**, 1–14 (2017).
